# AI-based differential diagnosis of dementia etiologies on multimodal data

**DOI:** 10.1038/s41591-024-03118-z

**Published:** 2024-07-04

**Authors:** Chonghua Xue, Sahana S. Kowshik, Diala Lteif, Shreyas Puducheri, Varuna H. Jasodanand, Olivia T. Zhou, Anika S. Walia, Osman B. Guney, J. Diana Zhang, Serena Poésy, Artem Kaliaev, V. Carlota Andreu-Arasa, Brigid C. Dwyer, Chad W. Farris, Honglin Hao, Sachin Kedar, Asim Z. Mian, Daniel L. Murman, Sarah A. O’Shea, Aaron B. Paul, Saurabh Rohatgi, Marie-Helene Saint-Hilaire, Emmett A. Sartor, Bindu N. Setty, Juan E. Small, Arun Swaminathan, Olga Taraschenko, Jing Yuan, Yan Zhou, Shuhan Zhu, Cody Karjadi, Ting Fang Alvin Ang, Sarah A. Bargal, Bryan A. Plummer, Kathleen L. Poston, Meysam Ahangaran, Rhoda Au, Vijaya B. Kolachalama

**Affiliations:** 1 Department of Medicine, Boston University Chobanian & Avedisian School of Medicine, Boston, MA USA; 2 Department of Electrical & Computer Engineering, Boston University, Boston, MA USA; 3 Faculty of Computing & Data Sciences, Boston University, Boston, MA USA; 4 Department of Computer Science, Boston University, Boston, MA USA; 5 School of Chemistry, University of New South Wales, Sydney, Australia; 6 Department of Radiology, Boston University Chobanian & Avedisian School of Medicine, Boston, MA USA; 7 Department of Neurology, Boston University Chobanian & Avedisian School of Medicine, Boston, MA USA; 8 Department of Neurology, Peking Union Medical College Hospital, Chinese Academy of Medical Sciences, Beijing, China; 9 Departments of Neurology & Ophthalmology, Emory University School of Medicine, Atlanta, GA USA; 10 Department of Neurological Sciences, University of Nebraska Medical Center, Omaha, NE USA; 11 Department of Neurology, Columbia University Irving Medical Center, New York, NY USA; 12 Department of Radiology, Massachusetts General Hospital, Boston, MA USA; 13 Department of Radiology, Lahey Hospital & Medical Center, Burlington, MA USA; 14 Department of Neurology, SSM Health, Madison, WI USA; 15 Department of Neurology, Brigham & Women’s Hospital, Boston, MA USA; 16 The Framingham Heart Study, Boston University Chobanian & Avedisian School of Medicine, Boston, MA USA; 17 Department of Anatomy and Neurobiology, Boston University Chobanian & Avedisian School of Medicine, Boston, MA USA; 18 Department of Computer Science, Georgetown University, Washington, DC USA; 19 Department of Neurology, Stanford University, Palo Alto, CA USA; 20 Boston University Alzheimer’s Disease Research Center, Boston, MA USA; 21 Department of Epidemiology, Boston University School of Public Health, Boston, MA USA

**Keywords:** Alzheimer's disease, Diagnostic markers

## Abstract

Differential diagnosis of dementia remains a challenge in neurology due to symptom overlap across etiologies, yet it is crucial for formulating early, personalized management strategies. Here, we present an artificial intelligence (AI) model that harnesses a broad array of data, including demographics, individual and family medical history, medication use, neuropsychological assessments, functional evaluations and multimodal neuroimaging, to identify the etiologies contributing to dementia in individuals. The study, drawing on 51,269 participants across 9 independent, geographically diverse datasets, facilitated the identification of 10 distinct dementia etiologies. It aligns diagnoses with similar management strategies, ensuring robust predictions even with incomplete data. Our model achieved a microaveraged area under the receiver operating characteristic curve (AUROC) of 0.94 in classifying individuals with normal cognition, mild cognitive impairment and dementia. Also, the microaveraged AUROC was 0.96 in differentiating the dementia etiologies. Our model demonstrated proficiency in addressing mixed dementia cases, with a mean AUROC of 0.78 for two co-occurring pathologies. In a randomly selected subset of 100 cases, the AUROC of neurologist assessments augmented by our AI model exceeded neurologist-only evaluations by 26.25%. Furthermore, our model predictions aligned with biomarker evidence and its associations with different proteinopathies were substantiated through postmortem findings. Our framework has the potential to be integrated as a screening tool for dementia in clinical settings and drug trials. Further prospective studies are needed to confirm its ability to improve patient care.

## Abstract (web summary)

Drawing on 51,269 participants across 9 independent, geographically diverse datasets, an AI model identifies the etiologies contributing to dementia in individuals, harnessing a broad array of data, including demographics, medical history, medication use, neuropsychological assessments, functional evaluations, and multimodal neuroimaging.

## Main

Dementia is one of the most pressing health challenges of our time. With nearly 10 million new cases reported annually, this syndrome, characterized by a progressive decline in cognitive function severe enough to impede daily life activities, continues to present considerable clinical and socioeconomic challenges. In 2017, the World Health Organization’s global action plan highlighted the need for prompt and precise diagnosis of dementia as a pivotal strategic objective in response to the growing number of dementia cases worldwide^1,2^. As such, diagnostic precision in the varied landscape of dementia remains a critical, yet unmet need, particularly as the global population ages and the demand for more accurate participant screening in drug trials increases^3^. This challenge primarily stems from the overlapping clinical presentation of different dementia types, which is further complicated by the heterogeneity in findings on magnetic resonance imaging (MRI) scans^4,5^. The necessity for improvements in the field becomes ever more pressing considering the projected shortage of specialists, including neurologists, neuropsychologists and geriatric care providers^6–8^, emphasizing the urgency to innovate and evolve our diagnostic tools.

Accurate differential diagnosis of dementia is pivotal for prescribing targeted therapeutic interventions, enhancing treatment efficacy and slowing symptom progression. Although Alzheimer’s disease (AD) is a leading cause, other forms such as vascular dementia (VD), Lewy body dementia (LBD) and frontotemporal dementia (FTD) are also prevalent^9–11^. These etiologies can often coexist, as marked by symptom overlap and variable symptom intensity, which further complicate the diagnostic process^12^. Importantly, diagnostic errors are prevalent among older adults, particularly those with comorbid conditions^13^. These misdiagnoses can translate into inappropriate medication use and adverse health outcomes^14^. For example, although patients with early-stage AD may be candidates for anti-amyloid therapies^15–17^, the coexistence of pathology from other etiologies, such as VD, can increase the risk of amyloid-related imaging abnormalities^18^. This risk highlights the critical need for accurately assessing the full spectrum of etiological factors contributing to dementia to inform appropriate therapeutic strategies and optimize patient care^19^.

The imperative for scalable diagnostic tools in AD and related dementias is becoming increasingly urgent, given the challenges in accessing gold-standard testing. Recent regulatory approvals have facilitated the transition of cerebrospinal fluid (CSF) and positron emission tomography (PET) biomarkers from research environments to clinical settings. Although promising, the clinical integration of accurate blood-based biomarkers remains an area of active research^20–22^. Despite these advancements, accessibility to these diagnostic tools is still constrained, not only in remote and economically developing regions but also in urban healthcare centers, as exemplified by prolonged waiting periods for specialist consultations^23^. This challenge is compounded by a global shortage of specialists, such as behavioral neurologists and neuropsychologists, leading to an overreliance on cognitive assessments that may not be culturally appropriate due to the lack of formal training programs in neuropsychology in many parts of the world^24,25^. Although conventional methods like clinical evaluations, neuropsychological testing and MRI remain central to antemortem differential dementia diagnosis, their effectiveness relies on a diminishing pool of specialist clinicians. This limitation underscores an urgent need for healthcare systems to evolve and adapt to the rapidly changing dynamics of dementia diagnosis and treatment.

Machine learning (ML) has the potential to enhance the accuracy and efficiency of dementia diagnosis^26–28^. Previous ML methods have largely focused on leveraging neuroimaging data to distinguish individuals with normal cognition (NC) from those with mild cognitive impairment (MCI) and dementia, with AD being the main etiology given its ubiquity in dementia diagnosis^29,30^. A few studies have attempted to discern neuroimaging signatures unique to AD by contrasting them with other dementia types^31–40^. However, this primary emphasis on AD can have limited practical implications given the prevalence and co-occurrence of other etiologies. In addition, a focus on imaging data alone can be insufficient in providing a holistic understanding of an individual’s neurological condition. Recently, we proposed a computational approach to stratify individuals based on cognitive status and discern likely AD cases from non-AD dementia types by incorporating imaging with non-imaging data such as demographics, medical histories and neuropsychological assessments^39^. These investigations have begun to illuminate the complex matrix of factors contributing to dementia. However, for ML models to be adopted into clinical practice, they must be able to accommodate the intricacies of mixed etiologies, as well as the inclusion or exclusion of different data modalities that may or may not be available. Therefore, the development of AI methodologies capable of harnessing multimodal data facilitates the accurate quantification of diverse dementia etiologies, irrespective of clinical resources, thereby aligning treatment strategies with individual patient profiles.

In this study, we propose a multimodal ML framework that harnesses a diverse array of data, including demographics, personal and family medical history, medication use, neuropsychological assessments, functional evaluations and multimodal neuroimaging, to perform differential dementia diagnosis. Our model, designed to mirror real-world scenarios, aligns diagnoses with similar management strategies and outputs probabilities for each etiology. This approach is intended to mimic clinical reasoning and aid practitioners in dementia screening and treatment planning. The model’s robustness is demonstrated through validation on independent, geographically diverse datasets. In comparative analyses, we found that AI-augmented clinician assessments achieved superior diagnostic accuracy compared to clinician-only assessments. By validating our model against gold-standard biomarker and postmortem data for different etiologies, we further emphasize our model’s ability to align with the pathophysiology underlying dementia. Our algorithmic framework has the potential to enhance dementia screening, but further studies are needed to evaluate its impact on healthcare outcomes.

## Results

**Table:** 

Glossary 1	
Acronym	Description
NC	Normal cognition
MCI	Mild cognitive impairment
DE	Dementia
AD	Alzheimer’s disease
LBD	Lewy body dementia, including dementia with Lewy bodies and Parkinson’s disease dementia
VD	Vascular dementia, vascular brain injury and vascular dementia, including stroke
PRD	Prion disease including Creutzfeldt-Jakob disease
FTD	Frontotemporal lobar degeneration and its variants, including primary progressive aphasia, corticobasal degeneration and progressive supranuclear palsy, and with or without amyotrophic lateral sclerosis
NPH	Normal pressure hydrocephalus
SEF	Systemic and environmental factors including infectious diseases (HIV included), metabolic, substance abuse / alcohol, medications, systemic disease and delirium
PSY	Psychiatric conditions including schizophrenia, depression, bipolar disorder, anxiety and posttraumatic stress disorder
TBI	Moderate/severe traumatic brain injury, repetitive head injury and chronic traumatic encephalopathy
ODE	Other dementia conditions, including neoplasms, Down syndrome, multiple systems atrophy, Huntington’s disease and seizures

**Fig. 1:**
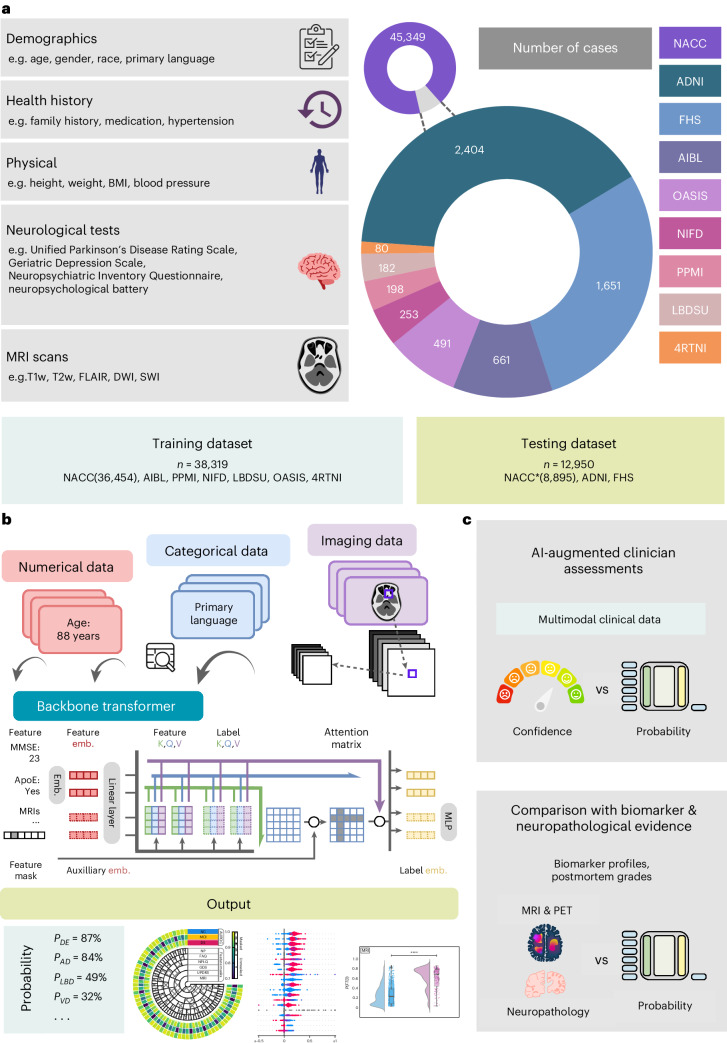
Data, model architecture and modeling strategy. **a**, Our model for differential dementia diagnosis was developed using diverse data modalities, including individual-level demographics, health history, neurological testing, physical/neurological exams and multisequence MRI scans. These data sources whenever available were aggregated from nine independent cohorts: 4RTNI, ADNI, AIBL, FHS, LBDSU, NACC, NIFD, OASIS and PPMI (Tables 1 and S1). For model training, we merged data from NACC, AIBL, PPMI, NIFD, LBDSU, OASIS and 4RTNI. We used a subset of the NACC dataset for internal testing. For external validation, we utilized the ADNI and FHS cohorts. **b**, A transformer served as the scaffold for the model. Each feature was processed into a fixed-length vector using a modality-specific embedding (emb.) strategy and fed into the transformer as input. A linear layer was used to connect the transformer with the output prediction layer. **c**, A subset of the NACC testing dataset was randomly chosen to conduct a comparative analysis between neurologists' performance augmented with the AI model and their performance without AI assistance. Similarly, we carried out comparative evaluations with practicing neuroradiologists, who were provided with a randomly selected sample of confirmed dementia cases from the NACC testing cohort, to assess the impact of AI augmentation on their diagnostic performance. For both these evaluations, the model and clinicians had access to the same set of multimodal data. Finally, we assessed the model’s predictions by comparing them with biomarker profiles and pathology grades available from the NACC, ADNI and FHS cohorts.

Leveraging the power of multimodal data obtained from various cohorts^41–49^ (Tables 1 and S1–S6), our model adopts a rigorous approach to differential dementia diagnosis (Fig. 1). It assigns individuals to one or more of thirteen diagnostic categories (Glossary 1), which were defined through consensus among a team of neurologists. This practical categorization is designed with clinical management pathways in mind, thereby echoing real-world scenarios. For instance, we grouped dementia with LBD and Parkinson’s disease (PD) dementia under the comprehensive category of LBD. This classification stems from an understanding that the care for these conditions often follows a similar path, typically overseen by a multidisciplinary team of movement disorder specialists. In the context of VD, we included persons who exhibited symptoms of a stroke, possible or probable VD or vascular brain injury. This design encompassed cases with symptomatic stroke, cystic infarct in cognitive networks, extensive white matter hyperintensity and/or executive dysfunction as the primary contributors to the observed cognitive impairment. The inclusion criteria were based on the expectation that such persons would typically receive care from clinicians specializing in stroke and vascular diseases. Likewise, we considered various psychiatric conditions, such as schizophrenia, depression, bipolar disorders, anxiety and posttraumatic stress disorder, under one category (PSY), acknowledging that their management predominantly falls within the expertise of psychiatric care providers. By aligning diagnostic categories with clinical care pathways, our model serves not only to classify an individual’s condition but also to direct appropriate management strategies.

**Table 1:** Study population

Dataset (group)	Age (y), mean ± s.d.	Male, *n* (%)	Education (y), mean ± s.d.	Race (White, Black, Asian, American Indian, Pacific, multirace), *n*	CDR, mean ± s.d.
**NACC**					
NC [n = 17,242]	71.25 ± 11.16	6,009, 34.85%	15.83 ± 2.98^	(13,266, 2541, 528, 109, 10, 575)^	0.05 ± 0.15
MCI [n = 7,582]	73.72 ± 9.81	3,615, 47.68%	15.16 ± 3.45^	(5,708, 1185, 231, 53, 5, 276)^	0.45 ± 0.18
AD [n = 16,131]	76.0 ± 10.31	7,234, 44.85%	14.52 ± 3.74^	(13,161, 1702, 354, 92, 10, 458)^	1.2 ± 0.73
LBD [n = 1,913]	75.01 ± 8.55	1,365, 71.35%	15.12 ± 3.63^	(1,659, 128, 39, 17, 0, 37)^	1.29 ± 0.78
VD [n = 1,919]	80.32 ± 8.76	947, 49.35%	14.15 ± 4.22^	(1,394, 332, 67, 2, 1, 68)^	1.22 ± 0.74
PRD [n = 114]	60.07 ± 10.36	62, 54.39%	14.8 ± 3.33^	(93, 5, 5, 0, 1, 1)^	1.95 ± 0.95
FTD [n = 2,898]	65.86 ± 9.36	1,603, 55.31%	15.45 ± 3.09^	(2,664, 69, 73, 4, 5, 39)^	1.2 ± 0.83
NPH [n = 138]	79.1 ± 9.24	69, 50.0%	15.0 ± 3.28^	(119, 10, 4, 0, 0, 4)^	1.18 ± 0.71
SEF [n = 808]	76.3 ± 11.15	413, 51.11%	14.6 ± 3.77^	(646, 95, 15, 5, 2, 31)^	1.11 ± 0.7
PSY [n = 2,700]	73.74 ± 10.78	1,102, 40.81%	14.13 ± 4.12^	(2,163, 238, 59, 14, 5, 87)^	1.1 ± 0.64
TBI [n = 265]	72.87 ± 11.23	192, 72.45%	14.42 ± 4.13^	(212, 27, 3, 2, 1, 11)^	1.11 ± 0.69
ODE [n = 1,234]	72.94 ± 12.14	654, 53.0%	14.5 ± 3.78^	(1,046, 93, 28, 5, 4, 36)^	1.2 ± 0.76
*P* value	< 1.0 × 10^−200^	<1.0 × 10^−200^	< 1.0 × 10^−200^	8.341 × 10^−145^	<1.0 × 10^−200^
**NIFD**					
NC [n = 124]	63.21 ± 7.27	56, 45.16%	17.48 ± 1.87^	(89, 0, 0, 0, 0, 3)^	0.03 ± 0.12^
FTD [n = 129]	63.66 ± 7.33	75, 58.14%	16.18 ± 3.29^	(109, 1, 1, 0, 0, 4)^	0.82 ± 0.54^
*P* value	6.266 × 10^−1^	5.246 × 10^−2^	2.606 × 10^−4^	6.531 × 10^−1^	4.333 × 10^−28^
**PPMI**					
NC [n = 171]	62.74 ± 10.12	109, 63.74%	15.82 ± 2.93	(163, 3, 2, 0, 0, 1)^	NA
MCI [n = 27]	68.04 ± 7.32	22, 81.48%	15.52 ± 3.08	(24, 1, 1, 0, 0, 1)	NA
*P* value	1.006 × 10^−2^	1.115 × 10^−1^	6.194 × 10^−1^	2.910 × 10^−1^	NA
**AIBL**					
NC [n = 480]	72.45 ± 6.22	203, 42.29%	NA	NA	0.03 ± 0.12
MCI [n = 102]	74.73 ± 7.11	53, 51.96%	NA	NA	0.47 ± 0.14
AD [n = 79]	73.34 ± 7.77	33, 41.77%	NA	NA	0.93 ± 0.54
*P* value	5.521 × 10^−3^	1.887 × 10^−1^	NA	NA	4.542 × 10^−158^
**OASIS**					
NC [n = 424]	71.34 ± 9.43	164, 38.68%	15.79 ± 2.62^	(53, 18, 1, 0, 0, 0)^	0.0 ± 0.02
MCI [n = 27]	75.04 ± 7.25	14, 51.85%	15.19 ± 2.76	(4, 1, 0, 0, 0, 0)^	0.52 ± 0.09
AD [n = 32]	77.44 ± 7.42	20, 62.5%	15.19 ± 2.8	(8, 1, 0, 0, 0, 0)^	0.86 ± 0.44
LBD [n = 4]	74.75 ± 5.67	4, 100.0%	16.0 ± 2.83	NA	1.0 ± 0.0
FTD [n = 4]	64.25 ± 8.61	3, 75.0%	16.5 ± 2.96	(4, 0, 0, 0, 0, 0)	1.25 ± 0.75
*P* value	7.789 × 10^−4^	3.239 × 10^−3^	5.507 × 10^−1^	8.735 × 10^−1^	2.855 × 10^−169^
**LBDSU**					
NC [n = 134]	68.77 ± 7.62	61, 45.52%	17.27 ± 2.47^	NA	NA
MCI [n = 35]	70.16 ± 8.41	26, 74.29%	16.6 ± 2.58	NA	NA
LBD [n = 13]	73.42 ± 7.81	8, 61.54%	16.77 ± 2.15	NA	NA
*P* value	1.033 × 10^−1^	7.863 × 10^−3^	3.243 × 10^−1^	NA	NA
**4RTNI**					
NC [n = 12]	68.08 ± 4.92	5, 41.67%	15.45 ± 2.57^	(12, 0, 0, 0, 0, 0)	0.0 ± 0.0
MCI [n = 31]	67.61 ± 7.0	11, 35.48%	16.68 ± 4.02	(25, 1, 2, 0, 1, 1)^	0.55 ± 0.15
FTD [n = 37]	69.14 ± 7.43	20, 54.05%	16.46 ± 4.21	(31, 1, 0, 0, 1, 2)^	1.27 ± 0.55
*P* value	6.691 × 10^−1^	2.992 × 10^−1^	6.843 × 10−1	7.620 × 10^−1^	5.700 × 10^−16^
**ADNI**					
NC [n = 868]	72.7 ± 6.57	383, 44.12%	16.51 ± 2.52	(730, 92, 28, 2, 0, 12)^	0.0 ± 0.04^
MCI [n = 1119]	72.77 ± 7.65	648, 57.91%	15.97 ± 2.75	(1,023, 56, 17, 2, 2, 13)^	0.5 ± 0.06
AD [n = 417]	74.99 ± 7.78	232, 55.64%	15.25 ± 2.92	(383, 20, 10, 0, 0, 4)	0.77 ± 0.27
*P* value	8.911 × 10^−8^	3.090 × 10-09	2.869 × 10^−14^	2.828 × 10^−5^	<1.0 × 10^−200^
**FHS**					*
NC [n = 394]	74.9 ± 10.22^	206, 52.28%	NA	(394, 0, 0, 0, 0, 0)	0.0 ± 0.0
MCI [n = 434]	79.92 ± 8.8^	203, 46.77%	NA	(434, 0, 0, 0, 0, 0)	0.49 ± 0.07
AD [n = 687]	82.99 ± 7.87^	211, 30.71%	NA	(687, 0, 0, 0, 0, 0)	2.04 ± 0.88
LBD [n = 73]	79.34 ± 9.37^	46, 63.01%	NA	(73, 0, 0, 0, 0, 0)	1.84 ± 0.84
VD [n = 113]	81.74 ± 7.3^	48, 42.48%	NA	(113, 0, 0, 0, 0, 0)	1.85 ± 0.8
FTD [n = 8]	85.67 ± 5.91^	4, 50.0%	NA	(8, 0, 0, 0, 0, 0)	2.0 ± 0.87
*P* value	1.316 × 10^−31^	7.905 × 10^−14^	NA	1.0	<1.0 × 10^−200^

Nine independent datasets were used for this study, including ADNI, NACC, NIFD, PPMI, OASIS, LBDSU, 4RTNI and FHS. Data from NACC, NIFD, PPMI, OASIS, LBDSU and 4RTNI were used for model training. Data from ADNI, FHS and a held-out set from NACC were used for model testing. The *P* value for each dataset indicates the statistical significance of intergroup differences per column. We used one-way analysis of variance (ANOVA) and two-sided *χ*^2^ tests for continuous and categorical variables, respectively. Please refer to Glossary 1 for more information on the acronyms. NA, not available. Due to the absence of CDR scores in the FHS dataset, we used the following definition: 0.0, NC; 0.5, cognitive impairment; 1.0, mild dementia; 2.0, moderate dementia; 3.0, severe dementia. The symbol ^ indicates that data was not available for some subjects.

### Model performance on NC, MCI and dementia

**Fig. 2:**
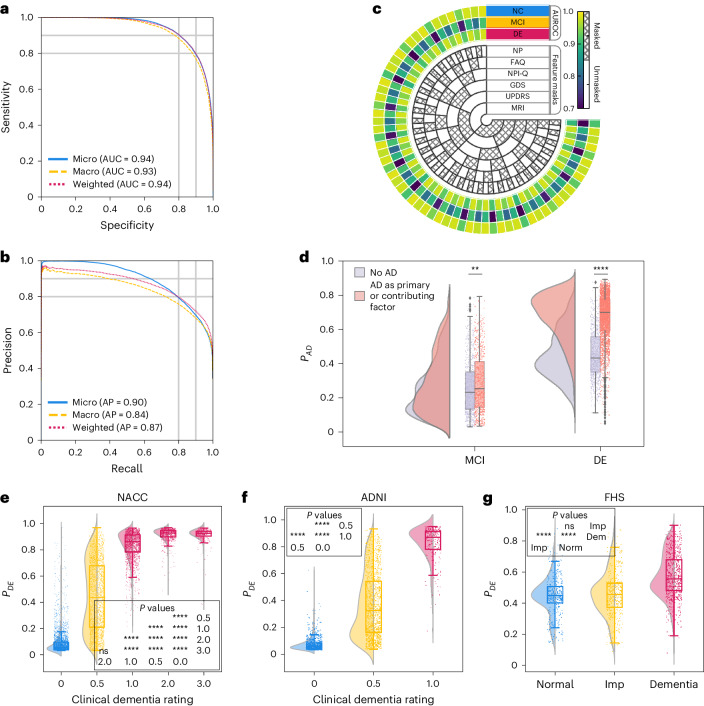
Model performance on individuals along the cognitive spectrum. **a**,**b**, ROC and PR curves, with their respective microaverage, macroaverage and weighted-average calculations based on the labels for NC, MCI and dementia. These averaging techniques consolidated the model’s performance across the spectrum of cognitive states. Cases from the NACC testing, along with all the cases from ADNI and FHS cohorts, were used. **c**, Diagram indicating varied levels of model performance in the presence of missing data. The inner concentric circles represent various scenarios in which particular test information was either omitted (masked) or included (unmasked). The three outer concentric rings depict the model’s performance as measured by the AUROC for the NC, MCI and dementia labels. **d**, Raincloud plots are used to demonstrate the model’s predicted AD probabilities for individuals with MCI and dementia in the NACC cohort. Two-sample two-sided unadjusted Kolmogorov-Smirnov (KS) test for goodness of fit was used to compare the cases where AD was a factor in cognitive impairment to those with non-AD etiologies in MCI (*n* = 1,486, KS = 0.09, *P* = 4.29 × 10^−3^) and dementia groups (*n* = 4,085, KS = 0.57, *P* < 1 × 10^−200^). **e**–**g**, Raincloud plots with violin and box diagrams are shown to denote the distribution of CDR scores (*x* axis) versus model-predicted probability of dementia (*y* axis), on the NACC, ADNI and FHS cohorts, respectively. We performed the Kruskal-Wallis *H*-test for independent samples in NACC (*n* = 8,895, *H* = 6,921.71, *P* < 1 × 10^−200^), ADNI (*n* = 2,400, *H* = 1,518.79, *P* < 1 × 10^−200^) and FHS (*n* = 1,651, *H* = 292.04, *P* = 3.84 × 10^−64^). These were followed by post-hoc Dunn’s testing with Bonferroni correction for multiple comparisons, and detailed statistical results are provided in Table S10. For **d**–**g**, each boxplot includes a box presenting the median value and interquartile range (IQR), with whiskers extending from the box to the maxima and minima no further than a distance of 1.5 times the IQR. Significance levels are denoted as ns (not significant) for *P* ≥ 0.05; *P < 0.05, **P < 0.01, ***P < 0.001, ****P < 0.0001. In **g**, ‘Normal’ indicates cognitively normal individuals, ‘Imp’ indicates those with cognitive impairment and ‘Dem’ indicates persons with mild, moderate and severe dementia.

We first sought to evaluate the performance of the model on test cases comprising individuals along the cognitive spectrum of NC, MCI and dementia. The receiver operating characteristic (ROC) and precision-recall (PR) curves reflected strong model performance across different averaging methods (Fig. 2a,b). In the test set, comprising the NACC data unused in training, the Alzheimer’s Disease Neuroimaging Initiative (ADNI) and the Framingham Heart Study (FHS) data, our model demonstrated robust classification abilities for NC, MCI and dementia, achieving a microaveraged area under the ROC curve (AUROC) of 0.94 and a microaveraged area under the PR curve (AUPR) of 0.90. Additionally, the macroaveraged metrics showed an AUROC of 0.93 and an AUPR value of 0.84. The weighted-average AUROC and AUPR values further demonstrated the model’s efficacy, standing at 0.94 and 0.87, respectively. Also, model performance across different age, gender and race subgroups was consistent for NC, MCI and dementia predictions. Microaveraged AUC exceeded 0.88 and microaveraged AUPR exceeded 0.82 across the different subgroups. Additional model performance metrics across the test cohorts and various demographic subgroups are provided in Table S7 and Figs. S1, S3 and S5, respectively. We also evaluated our model’s effectiveness by benchmarking it against a baseline ML algorithm, CatBoost^50^, using identical case sets. This comparison was executed over two feature subsets, revealing that our model and CatBoost exhibited similar performances on the NACC dataset. Conversely, on the ADNI and FHS datasets, our model surpassed CatBoost, achieving higher AUROC and AUPR scores across all diagnostic categories with improvements ranging from 0.02 to 0.21 for AUROC and 0.03 to 0.17 for AUPR, as detailed in Table S8. This comparison highlights the improved generalizability of our model over traditional ML approaches in diagnostic tasks.

**Extended Data Fig. 1:**
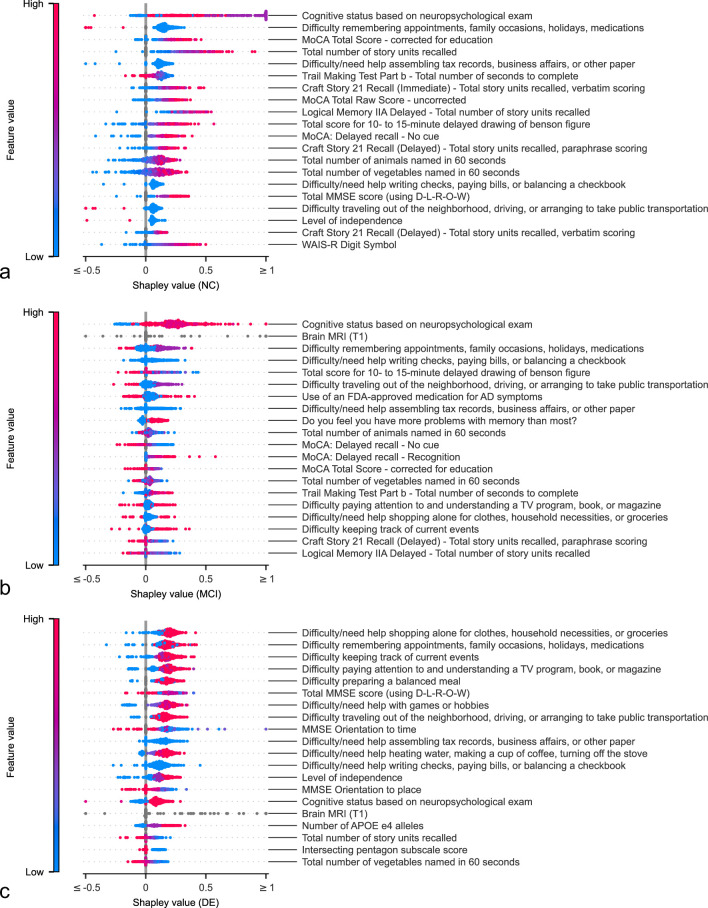
Shapley analysis on cases from the NACC test set comprising individuals along the cognitive spectrum. The figure presents the top twenty contributing features for the model’s positive predictions of **a**, NC, **b**, MCI, and **c**, DE labels, ranked by their mean Shapley values. These values, representing the average contribution of each feature to the model’s decision, guide the ranking from the highest to the lowest impact. For each diagnostic group, a subset of *n* = 500 cases with the most available features were selected for analysis.

Shapley analysis^51^ was used on the NACC test set to determine which features most influenced the model’s diagnostic decisions (Extended Data Fig. 1). For NC predictions, key features included cognitive status based on the neuropsychological exam, higher scores on the Montreal Cognitive Assessment (MoCA) and better performance on memory tasks. For MCI predictions, similar memory-related features were found to be important in addition to functional impairment and the T_1_-weighted (T1w) MRI. Finally, for dementia predictions, the most influential features related to functional impairment, lower Mini-Mental State Examination (MMSE) orientation to time and place subscores and the presence of APOE4 alleles. Overall, Shapley values offered insight into how each feature contributed to the model’s predictions, which is crucial for understanding and improving the model’s interpretability and accuracy.

### Model performance on incomplete data

To evaluate the model’s resilience to incomplete data, we artificially introduced varying levels of data missingness in the NACC cohort and assessed the impact on its predictive performance by selectively removing portions of the data to simulate different constraints. As depicted in the chord diagram (Fig. 2c), even when confronted with missing features, whether it be MRIs, the Unified Parkinson’s Disease Rating Scale, the Geriatric Depression Scale (GDS), the Neuropsychiatric Inventory Questionnaire, the Functional Activities Questionnaire (FAQ) NP tests or other parameters, our model consistently produced reliable scores. This reinforces not only its predictive stability but also its potential applicability in various clinical scenarios where complete datasets are generally unattainable. Examples of this are found in our results on ADNI and FHS, which we used as external testing datasets (Tables S4 and S5). The ADNI cohort exhibited approximately 69% missing data compared to NACC, yet model predictions achieved a weighted-average AUROC of 0.91 and AUPR of 0.86 for NC, MCI and dementia categories. Similarly, with 94% fewer features than NACC, the model’s performance on FHS data also resulted in weighted-average AUROC and AUPR scores of 0.68 and 0.53 for NC, MCI and dementia categories, respectively.

### Model alignment with prodromal AD

We sought to assess our model’s ability to distinguish MCI individuals based on whether AD was the etiological factor for their cognitive impairment by comparing the predicted probabilities of AD (*P*(*AD*)) between MCI cases with and without AD. For comparison, we also evaluated the model’s ability to differentiate individuals with dementia based on AD’s role in their cognitive impairment. Although our model was primarily trained to identify AD dementia rather than its prodromal stages, it consistently attributed higher *P*(*AD*) to MCI cases associated with AD compared to those arising from other causes, as evidenced in Fig. 2d and Table S9. In DE cases, the model generally assigned higher *P*(*AD*) to those where AD was the primary etiology. This pattern reinforces the model’s utility in early disease detection and in supporting clinicians to make informed decisions based on the specific etiology of cognitive impairment. Our observations advocate for a preemptive intervention approach in managing the AD continuum, underlining the model’s clinical significance.

### Model alignment with CDR scores

We conducted a comparison between the model’s predicted DE probability scores, *P*(*DE*), and the Clinical Dementia Ratings (CDR) scores available for all participants in the NACC testing and ADNI cohorts (Fig. 2e,f and Table S10). Despite not incorporating CDR as input during model training, our predictions exhibited a strong correlation with CDR scores. In our analysis of the NACC dataset, we observed that *P*(*DE*) progressively increased with higher CDR scores, with statistically significant differences manifest across the spectrum of cognitive impairment (*P* < 0.0001). However, this pattern did not hold between CDR scores of 2.0 and 3.0, where no significant statistical difference was discerned. In the ADNI dataset, we found a statistically significant demarcation (*P* < 0.0001) in *P*(*DE*) between the baseline CDR rating and higher gradations. This finding points to the model’s sensitivity to incremental impairment in clinical dementia assessments. In the FHS dataset (Fig. 2g), which substitutes a consensus panel’s diagnostic categorization (normal, impaired, and dementia) for CDR scores, a marked statistical significance (*P* < 0.0001) was evident in *P*(*DE*) across these diagnostic strata, with the exception of normal versus impaired. This finding indicates a challenge for the model in distinguishing the early stages of cognitive decline when relying on a limited set of features. Such limitations are likely due to the community-based nature of the FHS cohort and the specificities of consensus panel ratings at FHS (Table S4). Collectively, these findings illuminate the model’s robust capacity to delineate differential cognitive states, showcasing its potential as a tool for identifying levels of cognitive impairment across datasets.

### Evaluation of single and co-occurring dementias

**Fig. 3:**
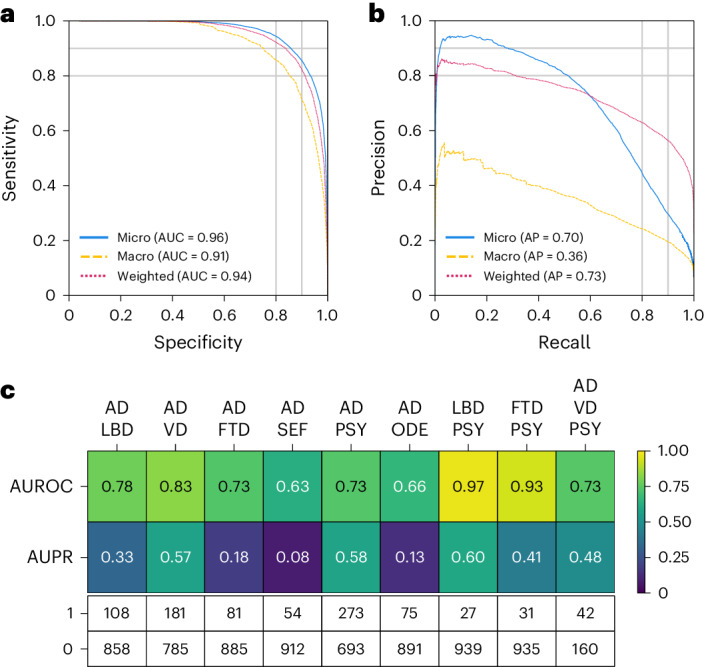
Model assessment on single and co-occurring dementias. **a**,**b**, ROC and PR curves are provided, using microaverage, macroaverage and weighted-average methods across all the dementia diagnostic labels. These averages were computed to synthesize the performance metrics across all dementia etiologies. Only cases from the NACC testing were used. **c**, Heatmaps are used to depict the model’s performance on co-occurring dementias. We considered all combinations where two or more etiologies co-occurred from the NACC testing cohort, provided there were at least 25 positive samples. This ensured that the maximum variance of the AUROC calculation over all possible continuous distributions was upper bounded by 0.01. The first row shows the AUROC values, and the second row shows the AUPR values. The table also displays the sample sizes for each case, with 1 representing a positive case and 0 indicating a negative sample. Only cases from the NACC testing were used.

We evaluated our model’s diagnostic ability across ten distinct dementia etiologies. The ROC and PR curves in (Fig. 3a,b) reflect strong model performance on the model’s overall assessment of identifying dementia etiologies across different averaging methods, attaining microaveraged AUROC and AUPR values of 0.96 and 0.70, respectively. In macroaveraged terms, the AUROC and AUPR stood at 0.91 and 0.36. Moreover, the weighted-average values for AUROC and AUPR were 0.94 and 0.73, respectively. The model’s performance, characterized by high microaveraged and weighted-average AUROC and AUPR scores, underscores its diagnostic accuracy across a broad spectrum of dementia etiologies. Although the lower macroaverage AUPR scores indicate that our model may perform better on certain diagnoses relative to others, the weighted-average scores, adjusting for the prevalence of each dementia type, support the model’s effectiveness in a real-world setting, where some dementia types are more common than others. The model exhibited stable performance across various demographic subgroups (that is, age, gender and race) with a microaveraged AUC consistently exceeding 0.94, and microaveraged AP exceeding 0.66. Additional model performance metrics across demographic subgroups are provided in Figs. S2, S4 and S6.

**Extended Data Fig. 2:**
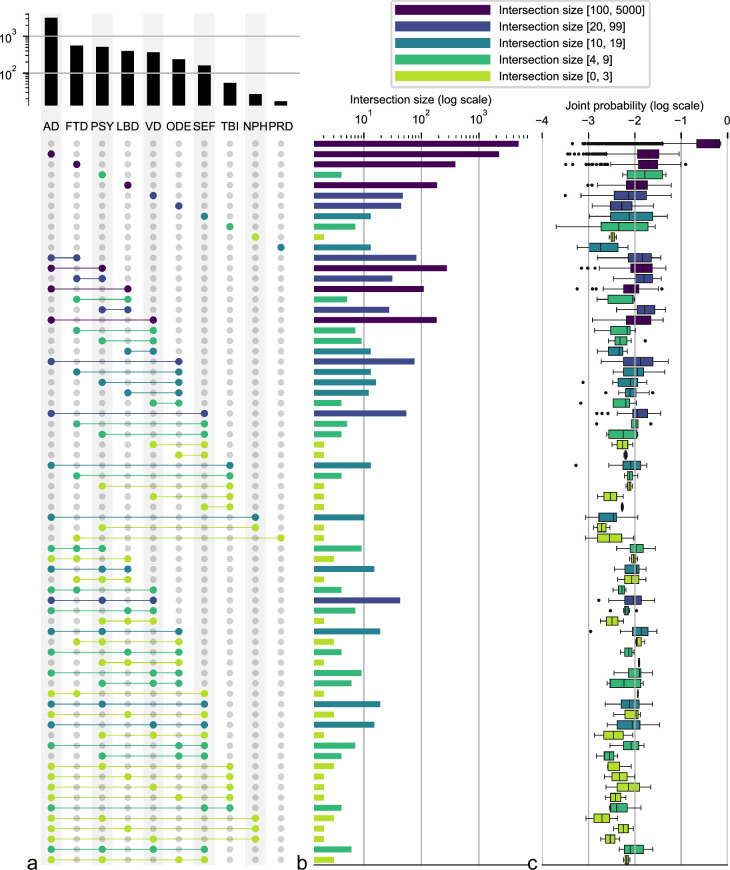
UpSet plot depicting the distribution and model-predicted probabilities of the etiological categories in NACC testing. **a**, Single and co-occurring diagnostic categories are enumerated, offering a tally of each condition’s frequency within the dataset. **b**, A logarithmic scale is used to delineate the overlap among these categories, shedding light on their relative commonality and the extent of their coexistence. This method grants a refined perspective on the prevalence of comorbid conditions. **c**, Boxplots delineating the spread and central tendency of the model’s predicted probabilities for each combination of diagnostic categories. The legend in the upper right interprets the sizes within **b** and **c**, providing a reference for the logarithmic data representation. All boxplots include a box presenting the median value and interquartile range (IQR), with whiskers extending from the box to the maxima and minima no further than a distance of 1.5 times the IQR.

To further assess the model performance on co-occurring dementias, we adopted a maximum variance threshold of 0.01 for AUROC calculations^52^. This selection aimed to balance the sensitivity and specificity of the model, enabling it to discern subtle diagnostic differences. This resulted in a minimum positive sample size of 25. In instances where two dementias co-occurred (Fig. 3c), the model’s AUROC scores varied from 0.63 to 0.97, reflecting a spectrum of diagnostic accuracy, with the LBD and PSY combination achieving the highest AUROC. AUPR scores ranged from 0.08 to 0.60, again with the conjunction of LBD and PSY recording the highest AUPR value. In the case of AD occurring with two other etiologies (VD and PSY), the AUROC score was 0.73 and the AUPR was 0.48. Although our model demonstrated robust diagnostic discrimination, as evidenced by high AUROC values, the variability in AUPR scores may reflect challenges in consistently identifying less prevalent or more complex dementia etiologies within the dataset. Importantly, a similar pattern was found in subsequent analyses of expert neurologists’ performance for conditions such as SEF and TBI (Tables S14 and S15). Additional performance metrics and visualizations that illustrate our model’s ability to assess single and co-occurring dementias are presented in the Supplement (Table S7 and Extended Data Fig. 2).

### Model validation with biomarkers

**Fig. 4:**
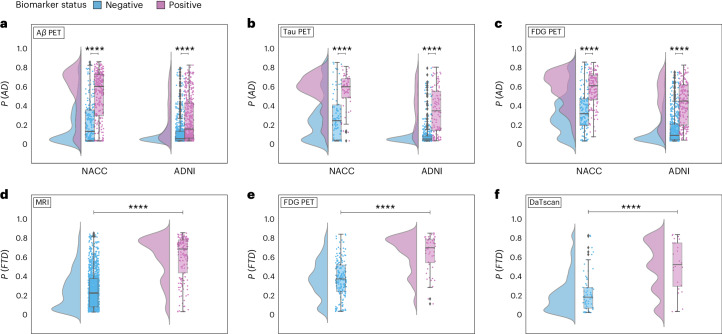
Biomarker-level validation. Raincloud plots representing model probabilities for dementia etiologies across their respective biomarker-negative (blue) and positive groups (pink). **a**, Model-predicted probabilities for AD, *P*(*AD*), were analyzed in relation to amyloid *β* (A*β*) positivity status using a one-sided Mann-Whitney *U* test for the NACC cohort (*n* = 440, *U* = 10,303.50, *P* = 2.04 × 10^−25^) and a one-sided t-test for ADNI (*n* = 1,108, *t* = −12.06, *P* = 9.74 × 10^−31^). **b**, Differences in *P*(*AD*) between tau PET negative and positive biomarker groups were analyzed using the one-sided Mann-Whitney *U* tests for NACC (*n* = 132, *U* = 935.50, *P* = 6.48 × 10^−8^) and ADNI (*n* = 475, *U* = 5,857.50, *P* = 4.10 × 10^−27^). **c**, Similar analyses were run to differentiate *P*(*AD*) between fluorodeoxyglucose (FDG) PET biomarker groups in NACC (*n* = 261, *U* = 3,730.00, *P* = 3.00 × 10^−15^), and ADNI (*n* = 760, *U* = 14,924.00, *P* = 5.66 × 10^−43^). **d, e**, In the NACC cohort, model-predicted probabilities for frontotemporal lobar degeneration, *P*(*FTD*), were assessed across MRI (*n* = 1,494, 30,935.50, *P* = 1.52 × 10^−51^) and FDG PET biomarker groups (*n* = 233, *U* = 1,599.50, *P* = 2.08 × 10^−13^) using a one-sided Mann-Whitney *U* test. **f**, In NACC, LBD probabilities, *P*(*LBD*), were analyzed between DaTscan negative and positive groups using a one-sided Mann-Whitney *U* test (*n* = 91, *U* = 318.50, *P* = 6.26 × 10^−6^). All boxplots presented include a box presenting the median value and IQR, with whiskers extending from the box to the maxima and minima no further than a distance of 1.5 times the IQR. In all plots, ****P < 0.0001, and results were not corrected for multiple comparisons.

Model-predicted probabilities for AD, FTD and LBD were aligned with the presence of respective biomarkers, as demonstrated in the raincloud plots in Fig. 4 and Table S11. For AD, *P*(*AD*) correlated with A*β*, tau and FDG PET biomarkers across the NACC and ADNI cohorts, indicating statistically significant differences between biomarker-negative and positive groups (*P* < 0.0001). Notably, *P*(*AD*) was consistently higher in A*β*, tau, and FDG PET positive groups, demonstrating that our framework’s diagnostic process aligns well with the current amyloid, tau, and neurodegeneration (ATN) criteria for AD diagnosis^53^. Within the NACC cohort, FTD probabilities, *P*(*FTD*), were significantly associated with MRI and FDG PET biomarkers, with the biomarker positive groups having higher *P*(*FTD*). This result corroborates the capability of our model to detect FTD in alignment with observed patterns of frontotemporal hypometabolism and atrophy^54^. Finally, LBD probabilities, *P*(*LBD*), also displayed a clear differentiation when analyzed in relation to dopamine transporter scan (DaTscan) evidence for LBD^55^, with the DaTscan-positive group exhibiting higher probabilities of LBD. Taken together, these findings validate the model’s effectiveness in capturing the pathophysiological underpinnings of prevalent dementia types in addition to the clinical syndrome, offering etiology-specific probability scores that closely match respective biomarker profiles. This alignment not only substantiates the model’s predictive validity but also highlights its relevance to contemporary clinical practice as its mechanism for differential diagnosis of dementia reflects established biomarker criteria.

### Model validation with neuropathological evidence

**Extended Data Fig. 3:**
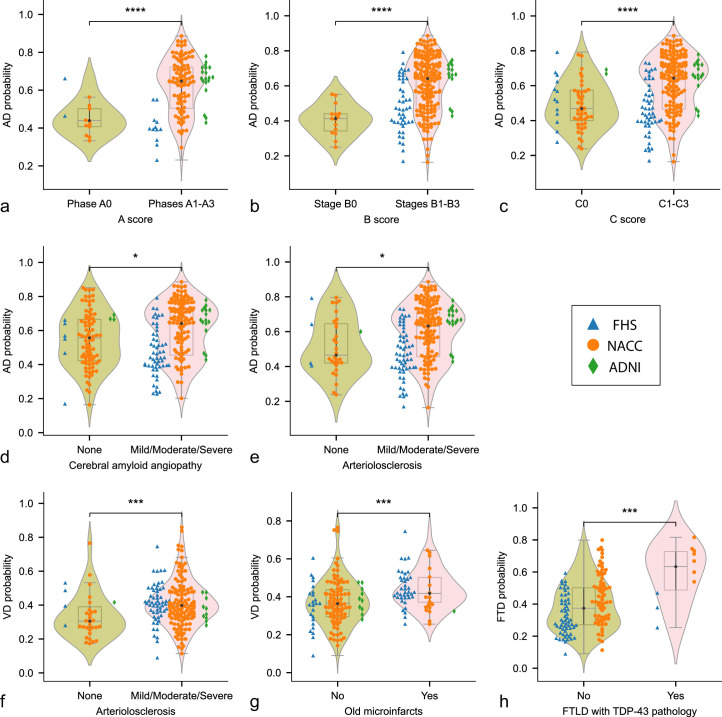
Neuropathological validation. Array of violin plots with integrated boxplots, delineating the model-predicted probabilities for different neuropathological grades across AD, VD and FTD etiologies. A one-sided Mann-Whitney U test was performed on data from FHS, NACC and ADNI, each denoted by unique markers. AD probabilities, *P(AD)*, were compared against three key AD pathological markers with progressive stages: **a**, Thal phases of A*β* plaques (*N* = 135, *U* = 282.5, *p* = 7.11*e* − 05), **b**, Braak stages of neurofibrillary degeneration (*N* = 249, *U* = 571.5, *p* = 6.07*e* − 06), and **c**, Consortium to Establish a Registry for Alzheimer’s Disease density scores of neocortical neuritic plaques (*N* = 278, *U* = 3916.5, *p* = 1.73*e* − 06). We further evaluated *P(AD)* against **d**, cerebral amyloid angiopathy (*N* = 274, *U* = 6938.5, *p* = 0.01) and **e**, arteriolosclerosis (*N* = 238, *U* = 2607.0, *p* = 0.01), both of which are common pathological findings in AD confirmed postmortem cases. Significant differences were also observed in model predicted probabilities for VD between cases with and without **f**, arteriolosclerosis (*N* = 230, *U* = 2085.5, *p* = 0.0002) and **g**, old microinfarcts (*N* = 178, *U* = 2289.5, *p* = 0.0001). **h**, Finally, model predicted probabilities for FTD differed significantly between cases with and without TDP-43 pathology (*N* = 136, *U* = 252.0, *p* = 0.0008). Table S13 also details these statistical results. No correction for multiple comparisons was performed and significance levels are illustrated as: * for *p* < 0.05; ** for *p* < 0.01; *** for *p* < 0.001; and **** for *p* < 0.0001. Each boxplot includes a box presenting the median value and interquartile range (IQR), with whiskers extending from the box to the maxima and minima no further than a distance of 1.5 times the IQR.

In cases with postmortem data (Table S12), we validated our model’s etiology-specific probability scores against neuropathological markers of common dementia types (Extended Data Fig. 3 and Table S13). The composite violin and boxplots indicate that, with increasing pathological severity, there is a corresponding elevation in the model-predicted probabilities of the etiology. The first three plots (Extended Data Fig. 3a–c) compare AD probabilities against three key AD pathological markers with progressive stages: Thal phases of A*β* plaques, Braak stages of neurofibrillary degeneration, and Consortium to Establish a Registry for Alzheimer’s Disease (CERAD) density scores of neocortical neuritic plaques, denoted by A1-A3, B1-B3 and C1-C3, respectively. Each demonstrated an upward shift in the median probability of AD and an expansion of the IQR as the stages advanced, with statistical significance (*p* < 0.0001 for Thal, Braak and CERAD stages, respectively). We further evaluated our model’s predicted probabilities against cerebral amyloid angiopathy (CAA) and arteriolosclerosis, both of which are common pathological findings in AD confirmed postmortem cases. Similarly, we observed that our model predicted significantly higher AD probabilities in individuals with mild, moderate, or severe CAA relative to those without CAA (*P* < 0.05) (Extended Data Fig. 3d), and in individuals with arteriolosclerosis (*P* < 0.05) (Extended Data Fig. 3e), underscoring the role of vascular factors in AD progression. Collectively, these plots illustrate a clear trend where advancing stages of AD-related pathology are associated with increased *P*(*AD*). Finally, significant differences were observed in *P*(*VD*) and *P*(*FTD*) based on their respective pathological markers; *P*(*VD*) varied between cases with and without arteriolosclerosis (*P* < 0.001) as well as old microinfarcts (*P* < 0.001), and *P*(*FTD*) differed significantly between cases with and without TDP-43 pathology (*P* < 0.001) (Extended Data Figs. 3f–h). The results are consistent with the well-documented association between cerebrovascular pathologies and the incidence of VD. Additionally, the clear linkage between TDP-43 protein aggregation and its prevalence in FTD is reinforced by our data^56,57^. Overall, these findings highlight the capability of our AI-driven framework to align model-generated probability scores with a range of neuropathological states beyond AD, supporting its potential utility in the evaluation of broader neurodegenerative diseases.

### AI-augmented clinician assessments

**Extended Data Fig. 4:**
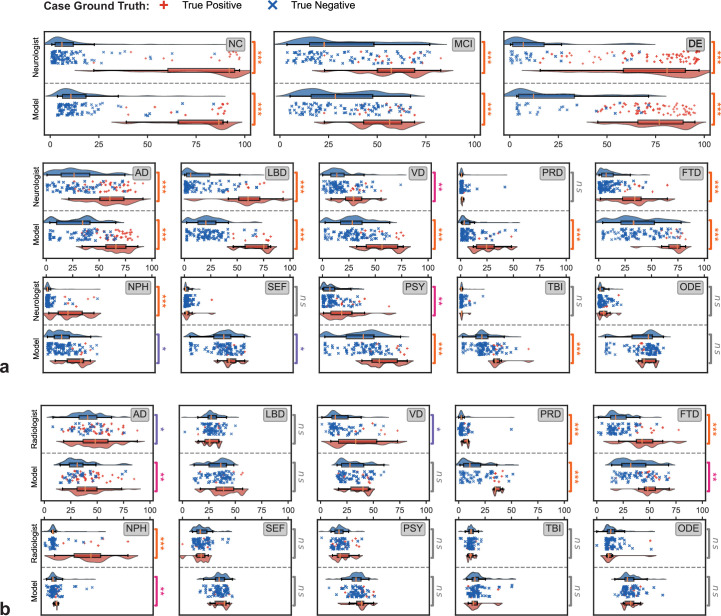
Head to head comparison between model and clinicians. Comparison between model-predicted probability scores and the assessments provided by practicing clinicians is shown. **a**, For the analysis, neurologists (*n* = 12) were given 100 randomly selected cases encompassing individual-level demographics, health history, neurological tests, physical as well as neurological examinations, and multisequence MRI scans. The neurologists were then tasked with assigning confidence scores for NC, MCI, DE, and the 10 dementia etiologies: AD, LBD, VD, PRD, FTD, NPH, SEF, PSY, TBI, and ODE (see Glossary 1). Neurologists’ confidence scores were averaged to produce a single consensus confidence score for each case. In the visual representation, the boxplot in blue indicates the distribution of confidence scores for true negative cases, while the boxplot in red signifies true positive cases. The symbol ‘+’ represents true positive cases, and ‘x’ denotes true negative cases. Significance levels are denoted as: ns (not significant) for *p*≥0.05; * for *p* < 0.05; ** for *p* < 0.01; *** for *p* < 0.001; and **** for *p* < 0.0001. These levels were determined using pairwise comparisons via the unadjusted two-sided Brunner-Munzel test, for which detailed pvalues and statistics can be found in Table S17. **b**, Similarly, in a separate analysis, radiologists (*n* = 7) were given 70 randomly selected cases with a confirmed dementia diagnosis encompassing individual-level demographics and multisequence MRI scans. The radiologists were tasked with assigning confidence scores for the 10 dementia etiologies. Similar to that of **a**, the visual representation consists of boxplots and scatterplots that represent the distribution of model and radiologists’ consensus confidence scores for true negative and true positive cases. Unadjusted two-sided Brunner-Munzel statistical test results are shown as pairwise annotations of ns, *, **, ***, or ****, and more detailed statistics and pvalues can be found in Table S18. Each boxplot presented includes a box presenting the median value and interquartile range (IQR), with whiskers extending from the box to the maxima and minima no further than a distance of 1.5 times the IQR.

**Extended Data Fig. 5:**
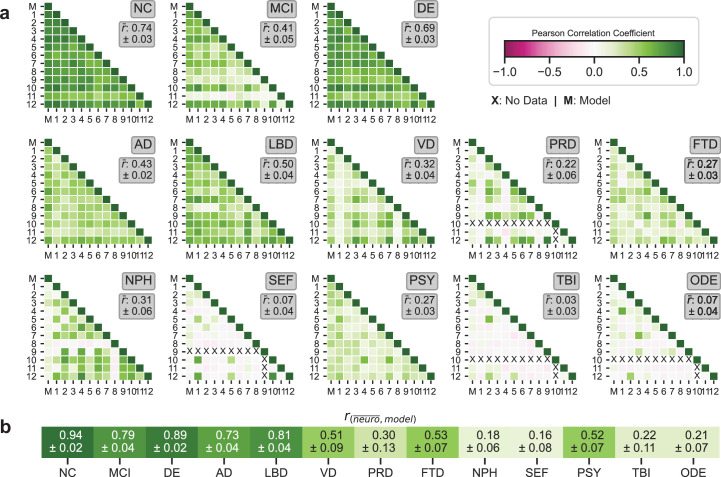
Neurologist and model interrater agreement. **a**, The figure presents the Pearson correlation coefficient across different diagnostic categories, comparing assessments from the neurologists (*n* = 12) and the model, marked as ‘M’. Each diagnostic category from NC to ODE includes a matrix reflecting correlation coefficient values between individual neurologists and the model. Shades of green signify positive correlation, indicating agreement between the model and neurologists, whereas magenta shades suggest negative correlations, indicating potential discrepancies in assessments. The mean pairwise Pearson correlation coefficient for each etiology is presented along with a 95% confidence interval. The symbol ‘X’ denotes rater pairs where the Pearson correlation was not calculable, due to one or both raters giving label-specific confidence scores with no variance. **b**, The heatmap shows the mean Pearson correlation coefficients between model probabilities and neurologist confidence scores for each label, along with its 95% confidence interval. The correlation coefficient and its confidence interval for each etiology were estimated with a non-parametric bootstrapping approach.

We aimed to assess whether our AI framework can compare to and enhance differential diagnosis of dementia performed by expert clinicians. To this end, we compared our model predicted probabilities with clinicians’ diagnoses, which were made in the form of confidence scores (0 to 100 scale). Neurologists reviewed 100 randomly selected cases, including various dementia subtypes, with comprehensive data including demographics, medical history, neuropsychological tests, and multisequence MRI scans. We observed that, in instances where the diagnosis was confirmed (true positives), the neurologists’ confidence scores across NC, MCI, dementia, AD, LBD, VD, FTD, NPH and PSY were higher in comparison to cases deemed non-diagnostic (true negatives) (*P* < 0.01) (Extended Data Fig. 4a and Table S17). In contrast, for the same 100 cases, our model’s predicted probabilities on true positive cases for all categories other than ODE were higher than the predicted probabilities for true negative cases (*P* < 0.01), indicating an enhanced ability for our model to detect true positives across more conditions (Extended Data Fig. 4a and Table S17). We then analyzed pairwise Pearson correlation coefficients to assess interrater agreement for each diagnostic category, both among neurologists’ confidence scores, and between the neurologists’ confidence scores and our model’s predicted probabilities (Extended Data Fig. 5a). Among clinicians’ assessments, we found the most robust, consistent associations within the NC and dementia groups, followed by modest associations between assessments of MCI, AD, LBD, VD, FTD and PSY. In contrast, PRD, NPH, SEF, TBI and ODE demonstrated the least consistency between neurologists’ assessments. This analysis shed light on dementia types that are relatively more challenging to diagnose, as evidenced by the variability in diagnostic confidence among expert clinicians. When comparing neurologists’ confidence scores with our model’s predicted probabilities, we found that the assessments provided by our model were generally consistent with those provided by the neurologists for NC, MCI, dementia, AD and LBD, as indicated by Pearson correlation coefficients that exceeded 0.7 (Extended Data Fig. 5b). Associations were modest for VD, FTD, PSY, where mean Pearson correlation coefficients were approximately 0.5, whereas associations were less consistent for PRD, NPH, SEF, TBI and ODE. The lower correlations observed here reflect the complex nature of these conditions, compounded by a lack of necessary features to tease out their unique signatures.

**Fig. 5:**
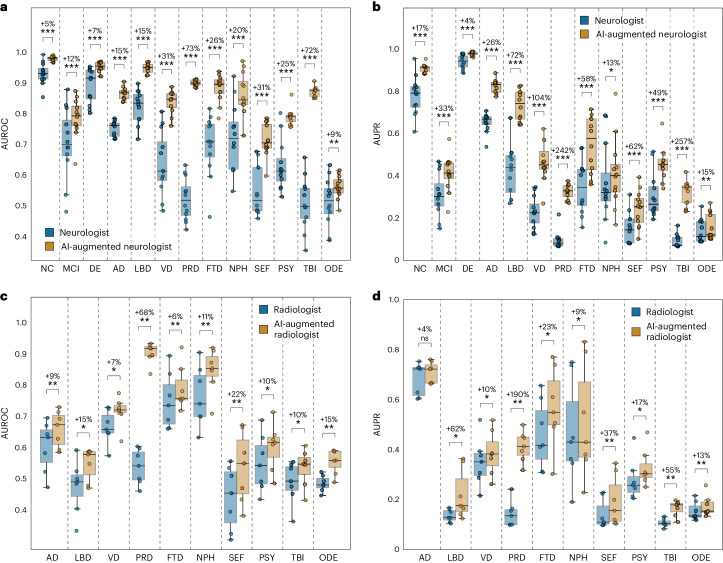
AI-augmented clinician assessments. Comparison between the performance of the assessments provided by practicing clinicians versus model-assisted clinicians is shown. **a**,**b**, For the analysis, neurologists (*n* = 12) were given 100 randomly selected cases encompassing individual-level demographics, health history, neurological tests, physical as well as neurological examinations, and multisequence MRI scans. The neurologists were then tasked with assigning confidence scores for NC, MCI, dementia and the 10 dementia etiologies: AD, LBD, VD, PRD, FTD, NPH, SEF, PSY, TBI and ODE (Glossary 1). The boxplots show AUROC in **a** and AUPR in **b** for individual neurologist and model-assisted neurologist performance (defined as the mean between model and neurologist confidence scores). Pairwise statistical comparisons were conducted using the one-tailed Wilcoxon signed-rank test without corrections made for multiple comparisons, with significance levels denoted as: ns (not significant) for *P* ≥ 0.05; *P < 0.05, **P < 0.01, ***P < 0.001 ****P < 0.0001. Detailed statistics and *P* values can be found in Table S14. The percent increase in mean performance for each etiology is also presented above each statistical annotation. **c**,**d**, Similarly, in a separate analysis, radiologists (*n* = 7) were given 70 randomly selected cases with a confirmed dementia diagnosis encompassing individual-level demographics and multisequence MRI scans. The radiologists were tasked with assigning confidence scores for the 10 dementia etiologies, and the boxplots show AUROC in **c** and AUPR in **d** for the individual radiologist and model-assisted radiologist performance for the 10 etiologies. Statistical annotations and percent increase in mean performance with respect to each etiology are shown in a similar fashion, with significance levels corresponding to the results of unadjusted one-tailed Wilcoxon signed-rank tests denoted as *, **, *** and ****. Detailed statistics and *P* values can be found in Table S15. Each boxplot includes a box presenting the median value and IQR, with whiskers extending from the box to the maxima and minima no further than a distance of 1.5 times the IQR.

To determine whether our model could augment the assessments provided by neurologists, we computed AI-assisted neurologist confidence scores, which was defined as the mean of the neurologists’ confidence scores and our model’s predicted probabilities. We then compared the diagnostic performance of individual neurologist assessments with that of AI-augmented neurologist assessments (Fig. 5a,b and Tables S14 and S15). We consistently found notable increases in AUROC and AUPR for all etiologies (*P* < 0.05). There was a mean percent increase in AUROC of 26.25% and a mean percent increase in AUPR of 73.23% across all categories. The greatest improvement in diagnostic performance was for PRD and TBI, where there was a percent increase in mean AUROC of 73% and 72%, respectively, and a percent increase in mean AUPR of 242% and 257%, respectively. In a separate assessment, neuroradiologists evaluated a randomly selected set of 70 clinically diagnosed dementia cases and were provided with multisequence MRIs, as well as demographic information. For these 70 cases, we found that our model was able to provide higher confidence scores for true positive cases (*P* < 0.01) across 4 of the 10 dementia etiologies (Extended Data Fig. 4b and Table S18). We also assessed the diagnostic performance of radiologists and AI-augmented radiologists, which was defined as the mean of the radiologists’ confidence scores and our model’s probabilities (Fig. 5c,d and Tables S14 and S15). Across various dementia etiologies, we observed an average increase of 16.19% in AUROC and 41.79% in AUPR. A significant enhancement in AUROC (*P* < 0.05) was noted across all etiologies other than TBI and ODE, with PRD showing the highest mean AUROC improvement of 69%. AUPR also displayed improvements across all etiologies, most markedly in PRD, where the mean AUPR surged by 200%.

## Discussion

We present an AI model designed for differential dementia diagnosis by processing a range of multimodal data. Unlike our previous work^39,58^, our model addresses the clinical challenge of distinguishing between various dementia etiologies, including but not limited to AD, VD and LBD. Such differentiation is crucial for the precise identification of the multifactorial nature of dementia, which is linked to the optimization of personalized therapeutic interventions and patient management strategies. The model’s robustness was established through its training and validation across a diverse set of independent cohorts. Additionally, our model predictions on various etiologies were corroborated by their validation on cases for which biomarker and postmortem data were available. In a randomly selected subset of cases, our model’s predictions, when combined with neurologist assessments, outperformed the assessments conducted by neurologists alone. These results underscore our model’s potential in enhancing the efficacy of diagnosing dementia-related disorders.

Our model is designed to address the complex nature of mixed dementias by providing probability scores for each contributing etiology. This approach is important as it enables clinicians to systematically prioritize possible drivers of cognitive impairment based on available data. The model effectively captures the multifactorial and overlapping characteristics of various dementia types, offering a clear framework to guide clinical decision-making. For example, misdiagnoses in the initial stages of dementia are frequent, often due to symptom misattribution to psychiatric disorders, a situation further complicated by the presence of multiple co-pathologies^59,60^. Although such misdiagnoses could also be present in the training data, our validated model can act as a tool to help standardize practice, potentially reducing variability in clinical assessments. Specifically, LBD has historically been difficult to diagnose as early symptoms often resemble those of AD and PSY. The co-occurrence of LBD and AD further complicates diagnosis and tends to be missed entirely until postmortem evaluation^61^. Our model demonstrated notable performance, particularly in identifying the AD and LBD combination, highlighting its capability to detect mixed dementias that are commonly recognized only through postmortem analysis^4,62,63^. This capability is crucial, given that a considerable portion of dementia cases are linked to modifiable risk factors^64^. The insights provided by our model could therefore inform early intervention strategies, potentially altering the disease course and enhancing patient outcomes. Notably, our model represents a step forward in the field by tackling the detection of mixed dementias, thereby offering a valuable tool for refining diagnostic accuracy in clinical practice.

Powered by a transformer architecture as the backbone, the utility of our modeling framework is founded on its robust processing of diverse input types and its adept handling of incomplete datasets through random feature masking. These properties are essential for clinicians requiring immediate and accurate diagnostic information in environments with variable data availability. For example, when a general practitioner records clinical observations and cognitive test results for an elderly person with possible cognitive decline, our model can calculate a probability score indicative of MCI or dementia. This function facilitates early medical intervention and more informed decisions regarding specialist referrals. At a specialized memory clinic, the addition of extensive neuroimaging data and in-depth neuropsychological battery to the model may increase the precision of the diagnosis, which, in turn, enhances the formulation of individual management strategies with a revised probability score. Such capacity to tailor its output to the scope of input data exemplifies our modeling framework’s role in different healthcare settings, including those where swift and resource-efficient diagnosis is paramount. The generation of specific, quantifiable probability scores by the model augments its utility, establishing it as a useful component in the healthcare delivery process. Displaying diagnostic accuracy using varied training data, ranging from demographic information to clinical signs, neuroimaging findings and neurological test results, the model’s versatility facilitates its adaptation to varied clinical operations without necessitating a fundamental overhaul of existing workflows. To further increase the robustness of our results and test the efficacy of the tool for dementia care, prospective studies and clinical trials are necessary. These steps will help validate the model’s potential and ensure it meets the needs of general practitioners and specialists across healthcare settings. Consequently, our model can foster a seamless transition across the different levels of dementia care, enabling general practitioners to perform preliminary cognitive screenings and specialists to conduct thorough examinations. Its inclusive functionality assures an accessible and comprehensive tool ensuring fail-safe operation in early detection, continuous monitoring and the fine-tuning of differential diagnoses, thereby elevating the standard of dementia care.

Although our study has the potential to advance the field of differential dementia diagnosis, it does have some limitations. Our model was developed and validated on 9 distinct cohorts, but its full generalizability across diverse populations and clinical settings remains to be determined as the dataset comprised a predominantly White population. Although our model is adept at handling missing data, the current results suggest that its performance may vary when applied to cohorts beyond NACC, such as ADNI and FHS, highlighting the need for further research to enhance its generalizability across diverse populations. Moving forward, we see potential in evaluating the model’s efficacy across the care continuum, encompassing primary care facilities, geriatric and general neurology practices, family medicine, and specialized clinics in tertiary medical centers. Furthermore, AI models like ours possess the capability to enhance patient screening procedures for clinical trial recruitment^65^. Our study’s datasets primarily consist of AD cases, and although AD is the most common type of dementia, this could potentially skew our model towards improved recognition of this specific subtype, introducing a bias. Although we incorporated various dementia etiologies, the imbalanced representation might affect the model’s generalizability and sensitivity towards less frequent types. It is important to note that, beyond data imbalance, certain conditions were inherently more challenging to assess given the available feature set, as exemplified by the lower performance of expert neurologists in diagnosing conditions such as SEF and TBI. This challenge is compounded by the fact that annotations used for model training can be uncertain or inconsistent as diagnostic decisions can vary among clinicians due to subjective interpretations of symptoms and variability in available information. Our training data might reflect these uncertainties, potentially affecting the model’s accuracy. However, the use of AI models in this context also presents an opportunity. By systematically analyzing large datasets, AI can help identify patterns that may be less apparent in individual cases, which can reduce variability in clinical assessments. Models trained on uncertain annotations can also be refined and improved over time as more accurate and comprehensive data become available. This iterative learning process can enhance the model’s reliability and utility in diagnosing complex conditions. Additionally, we chose to amalgamate mild, moderate, and severe dementia cases into a single category. We acknowledge that this categorization method might not completely reflect the nuanced individual staging practiced in specific healthcare settings, where varying degrees of dementia severity carry distinct implications for treatment and management strategies. Our focus was primarily on differential diagnosis rather than disease staging, which motivated this decision. Future enhancements to our model could potentially include disease staging as an additional dimension, thereby augmenting its granularity and relevance. Finally, our study does not fully address the considerable heterogeneity inherent in AD, which is characterized by diverse clinical presentations and pathological features^66,67^. Future studies are needed to rigorously evaluate AD heterogeneity by conducting stratified analyses based on specific clinical and pathological subtypes to understand how the model performs across different AD variants.

The evidence collected from this study signals a convergence between advanced computational methods and the task of differential dementia diagnosis, crucial for scenarios with scarce resources and the complex challenge of mixed dementia, a condition frequently encountered yet diagnostically complex. Our model efficiently integrates multimodal data, showing strong performance across diverse settings. Future validations, such as large-scale prospective cohort studies and multi-center clinical trials, encompassing a wider demographic and geographical expanse, will be pivotal to substantiate the model’s robustness and enhance its diagnostic utility in dementia care. Additionally, longitudinal studies tracking patient outcomes and comparative effectiveness research against current standard practices are essential to confirm the clinical usefulness of our tool. Our pragmatic investigation accentuates the potential of neural networks to refine the granularity of diagnostic evaluations in neurocognitive disorders.

## Methods

### Study population

We collected demographics, personal and family history, laboratory results, findings from the physical/neurological exams, medications, neuropsychological tests, and functional assessments as well as multisequence magnetic resonance imaging (MRI) scans from 9 distinct cohorts, totaling 51,269 participants. All participants or their designated informants provided written informed consents. All protocols received approval from the respective institutional ethical review boards of each cohort. There were 19,849 participants with NC, 9,357 participants with MCI and 22,063 participants with dementia. We further identified 10 primary and contributing causes of dementia: 17,346 participants with AD; 2,003 participants with dementia with LBD and PD (LBD); 2,032 participants with vascular brain injury or VD including stroke (VD); 114 participants with Prion disease including Creutzfeldt-Jakob disease (PRD); 3,076 participants with frontotemporal lobar degeneration (FTD) and its variants, which includes corticobasal degeneration (CBD) and progressive supranuclear palsy (PSP), and with or without amyotrophic lateral sclerosis (FTD); 138 participants with normal pressure hydrocephalus (NPH); 808 participants with dementia due to infections, metabolic disorders, substance abuse (including alcohol, medications), delirium and systemic disease, a category termed as systemic and external factors (SEF); 2,700 participants with psychiatric diseases, including schizophrenia, depression, bipolar disorder, anxiety and posttraumatic stress disorder (PSY); 265 participants with dementia due to traumatic brain injury (TBI); and 1,234 participants with dementia due to other causes, which include neoplasms, multiple systems atrophy, essential tremor, Huntington’s disease, Down syndrome and seizures (ODE).

The cohorts include the National Alzheimer’s Coordinating Center (NACC) dataset (*n* = 45,349)^41^, the ADNI dataset (*n* = 2,404)^48^, the FTD neuroimaging initiative (NIFD) dataset (*n* = 253)^46^, the Parkinson’s Progression Marker Initiative (PPMI) dataset (*n* = 198)^45^, the Australian Imaging, Biomarker and Lifestyle Flagship Study of Ageing (AIBL) dataset (*n* = 661)^43^, the Open Access Series of Imaging Studies-3 (OASIS) dataset (*n* = 491)^42^, the 4 Repeat Tauopathy Neuroimaging Initiative (4RTNI) dataset (*n* = 80)^44^ and three in-house datasets maintained by the Lewy Body Dementia Center for Excellence at Stanford University (LBDSU) (*n* = 182)^47^ and the FHS (*n* = 1,651)^49^. Since its inception in 1948, FHS has been dedicated to identifying factors contributing to cardiovascular disease, monitoring multiple generations from Framingham, Massachusetts. Over time, the study has pinpointed major cardiovascular disease risk factors and explored their effects while also investigating risk factors for conditions like dementia and analyzing the relationship between physical traits and genetics. Additional details on the study population are presented in Tables 1 and S1.

### Inclusion and exclusion criterion

Individuals from each cohort were eligible for study inclusion if they were diagnosed with NC, MCI or dementia. We used the NACC dataset^41^, which is based on the Uniform Data Set (UDS) 3.0 dictionary^68^, as the baseline for our study. To ensure data consistency, we organized the data from the other cohorts according to the UDS dictionary. For individuals from the NACC cohort who had multiple clinical visits, we initially prioritized the visits at which the person received the diagnostic label of dementia. We then selected the visit with the most data features available prioritizing the availability of neuroimaging information. If multiple visits met all the above criteria, we chose the most recent visit among them. This approach maximized the sample sizes of dementia cases and ensured that each individual had the latest record included in the study while maximizing the utilization of available neuroimaging and non-imaging data. We included participants from the 4RTNI dataset^44^ with FTD-related disorders like PSP or CBS. For other cohorts (NIFD^46^, PPMI^45^, LBDSU^47^, AIBL^43^, ADNI^48^ and OASIS^42^), participants were included if they had at least one MRI scan within 6 months of an officially documented diagnosis. From the FHS^49^, we used data from the Original Cohort (Gen 1) enrolled in 1948 and the Offspring Cohort (Gen 2) enrolled in 1971. For these participants, we selected available data including demographics, history, clinical exam scores, neuropsychological test scores and MRI within 6 months of the date of diagnosis. We did not exclude cases based on the absence of features (including imaging) or diagnostic labels. Instead, we used our innovative model training approach to address missing features or labels (see below).

### Data processing and training strategy

Various non-imaging features (*n* = 391) corresponding to subject demographics, medical history, laboratory results, medications, neuropsychological tests and functional assessments were included in our study. We combined data from 4RTNI, AIBL, LBDSU, NACC, NIFD, OASIS and PPMI to train the model. We used a portion of the NACC dataset for internal testing, whereas the ADNI and FHS cohorts served for external validation (Tables 1 and S1–S5). We used a series of steps such as standardizing the data across all cohorts and formatting the features into numerical or categorical variables before using them for model training. We used stratified sampling at the person-level to create the training, validation and testing splits. As we pooled the data from multiple cohorts, we encountered challenges related to missing features and labels. To address these issues and enhance the robustness of our model against data unavailability, we incorporated several strategies such as random feature masking and masking of missing labels (see below).

### MRI processing

Our investigation harnessed the potential of multisequence magnetic resonance imaging (MRI) volumetric scans sourced from diverse cohorts (Table S6). Most of these scans encompassed T1-weighted (T1w), T_2_-weighted (T2w), diffusion-weighted imaging (DWI), susceptibility-weighted imaging (SWI) and fluid-attenuated inversion recovery (FLAIR) sequences. The collected imaging data were stored in the NIFTI file format, categorized by participant and the date of their visit. The MRI scans underwent a series of pre-processing steps involving skull stripping, linear registration to the MNI space and intensity normalization. Skull stripping was performed using SynthStrip^69^, a computational tool designed for extracting brain voxels from various image types. Then, the MRI scans were registered using FSL’s ‘flirt’ tool for linear registration of whole brain images^70^, based on the MNI152 atlas^71^. Before linear registration to the MNI space, we used the ‘fslorient2std’ function within FSL to standardize the orientation across all scans to match the MNI template’s axis order. As a result, the registered scans followed the dimensions of the MNI152 template, which are 182 × 218 × 182. Finally, all MRI scans underwent intensity normalization to the range [0,1] to increase the homogeneity of the data. To ensure the purity of the dataset, we excluded calibration, localizer and 2D scans from the downloaded data before initiating model training. Consequently, as our DWI sequences were acquired in 2D, they were not considered for model training.

### Backbone architecture

Our modeling framework harnesses the power of the transformer architecture to interpret and process a vast array of diagnostic parameters, including person-level demographics, medical history, neuroimaging, functional assessments and neuropsychological test scores. Each of these distinct features is initially transformed into a fixed-length vector using a modality-specific strategy, forming the initial layer of input for the transformer model. Following this, the transformer acts to aggregate these vector inputs, decoding them into a series of predictions. A distinguishing strength of this framework lies in its integration of the transformer’s masking mechanism^72,73^, strategically deployed to emulate missing features. This capability enhances the model’s robustness and predictive power, allowing it to adeptly handle real-world scenarios characterized by incomplete data.

### Multimodal data embeddings

Transformers use a uniform representation for all input tokens, typically in the form of fixed-length vectors. However, the inherent complexity of medical data, with its variety of modalities, poses a challenge to this requirement. Therefore, medical data needs to be adapted into a unified embedding that our transformer model can process. The data we accessed fall into three primary categories: numerical data, categorical data and imaging data. Each category requires a specific method of embedding. Numerical data typically encompass those data types where values are defined in an ordinal manner that holds distinct real-world implications. For instance, chronological age fits into this category, as it serves as an indicator of the aging process. To project numerical data into the input space of the transformer, we used a single linear layer to ensure appropriate preservation of the structure inherent to the original data space. Categorical data encompass those inputs that can be divided into distinct categories yet lack any implicit order or priority. An example of this is gender, which can be categorized as ‘male’ or ‘female’. We used a lookup table to translate categorical inputs into corresponding embeddings. It is noteworthy that this approach is akin to a linear transformation when the data is one-hot vectorized but is computationally efficient, particularly when dealing with a vast number of categories. Imaging data, which includes MRI scans in medical applications, can be seen as a special case of numerical data. However, due to their high dimensionality and complexity, it is difficult to compress raw imaging data into a lower-dimensionality vector using a linear transformation while still retaining essential information. We leveraged the advanced capabilities of modern deep learning architectures to extract meaningful imaging embeddings (see below). Once these embeddings were generated, they were treated as numerical data, undergoing linear projection into vectors of suitable length, thus enabling their integration with other inputs to the transformer.

### Imaging feature extraction

**Extended Data Fig. 6:**
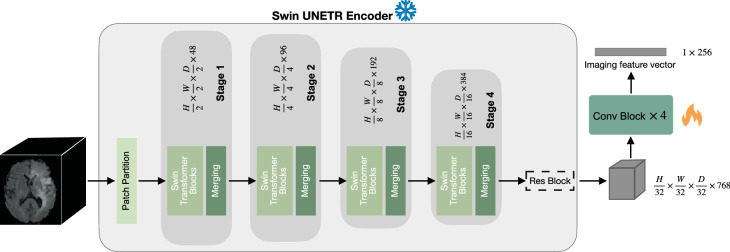
Image feature extraction. The Swin UNETR encoder, utilizing pre-trained weights, was leveraged to extract image embeddings from multi-sequence MRI scans into a latent space representation. Subsequently, these embeddings underwent a series of downsampling convolutional operations to achieve a condensed token dimension of 1 × 256. This dimensional reduction facilitated a consistent input format for both imaging and non-imaging data into the backbone transformer. Within this architecture, the Swin UNETR encoder’s weights remained static (frozen), ensuring the integrity of the pre-trained features, while the downsampling blocks were subject to optimization during the training phase, allowing for adaptive learning of the imaging feature vector.

We harnessed the Swin UNETR (Extended Data Fig. 6)^74,75^, a three-dimensional (3D) transformer-based architecture, to extract embeddings from a multitude of brain MRI scans, encompassing various sequences including T1w, T2w, SWI and FLAIR imaging sequences. The Swin UNETR model consists of a Swin Transformer encoder, designed to operate on 3D patches, seamlessly connected to a convolutional neural network-based decoder through multi-resolution skip connections. Commencing with an input volume $X\in {{\mathbb{R}}}^{H\times W\times D}$, the encoder segmented *X* into a sequence of 3D tokens with dimensions $\frac{H}{{H}^{{\prime} }}\times \frac{W}{{W}^{{\prime} }}\times \frac{D}{{D}^{{\prime} }}$, and projected them into a *C*-dimensional space via an embedding layer. It employed a patch size of 2 × 2 × 2 with a feature dimension of 2 × 2 × 2 × 1 and an embedding space dimension of *C* = 48. The Swin UNETR encoder was subsequently interconnected with a convolutional neural network-based decoder at various resolutions through skip connections, collectively forming a ‘U-shaped’ network. This decoder amalgamated the encoder’s outputs at different resolutions, conducted upsampling via deconvolutions, ultimately generating a reconstruction of the initial input volume. The pre-trained weights were the product of self-supervised pre-training of the Swin UNETR encoder, primarily conducted on 3D volumes encompassing the chest, abdomen and head/neck^74,75^.

The process of obtaining imaging embeddings began with several transformations applied to the MRI scans. These transformations included resampling the scans to standardized pixel dimensions, foreground cropping, and spatial resizing, resulting in the creation of subvolumes with dimensions of 128 × 128 × 128. Subsequently, these subvolumes were input into the Swin UNETR model, which in turn extracted encoder outputs sized at 768 × 4 × 4 × 4. These extracted embeddings underwent downsampling via a learnable embedding module, consisting of four convolutional blocks, to align with the input token size of the downstream transformer. As a result, the MRI scans were effectively embedded into one-dimensional vectors, each of size 256. These vectors were then combined with non-imaging features and directed into the downstream transformer for further processing. The entire process used a dataset comprising 8,155 MRI volumes, which were allocated for model training, validation and testing (Table S6).

### Random feature masking

To enhance the robustness of the backbone transformer in handling data incompleteness, we leveraged the masking mechanism^72,73^ to emulate arbitrary missing features during training. The masking mechanism, when paired with the attention mechanism, effectively halts the information flow from a given set of input tokens, ensuring that certain features are concealed during prediction. A practical challenge arises when considering the potential combinations of input features, which increase exponentially. With hundreds of features in play, capturing every potential combination is intractable. Inspired by the definition of Shapley values, we deployed an efficient strategy for feature dropout. Given a sample with a feature set *S*, *S* is randomly permuted as *σ*; simultaneously, an integer *i* is selected independently from the range $\left[1,| S| \right]$. Subsequent to this, the features *σ*_*i*+1_, *σ*_*i*+2_, …, *σ*_∣*S*∣_ are masked out from the backbone transformer. It is noteworthy that the dropout process was applied afresh across different training batches or epochs to ensure that the model gets exposed to a diverse array of missing information even within a single sample.

### Handling missing labels

The backbone transformer was trained by amalgamating data from multiple different cohorts, each focused on distinct etiologies, which introduced the challenge of missing labels in the dataset. While most conventional approaches involve discarding records with incomplete output labels during training, we chose a more inclusive strategy to maximize the utility of the available data. Our approach framed the task as a multilabel classification problem, introducing thirteen separate binary heads, one for each target label. With this design, for every training sample, we generated a binary mask indicating the absence of each label. We then masked the loss associated with samples lacking specific labels before backpropagation. This method ensured optimal utilization of the dataset, irrespective of label availability. The primary advantage of this approach lies in its adaptability. By implementing this label-masking strategy, our model can be evaluated against datasets with varying degrees of label availability, granting us the flexibility to address a wide spectrum of real-world scenarios.

### Loss function

Our backbone model was trained by minimizing the loss function (${{{\mathcal{L}}}}$) composed of two loss terms: ‘focal loss (FL)’^76^ (${{{{\mathcal{L}}}}}_{{{{\rm{FL}}}}}$) and ‘ranking loss (RL)’ (${{{{\mathcal{L}}}}}_{{{{\rm{RL}}}}}$), along with the standard L2 regularization term. FL is a variant of standard cross-entropy loss that addresses the issue of class imbalance; it assigns low weight to easy (well-classified) instances and employs a balance parameter. This loss function was used for each of the diagnostic categories (a total of 13; Glossary 1). Therefore, our ${{{{\mathcal{L}}}}}_{{{{\rm{FL}}}}}$ term was:

$${{{{\mathcal{L}}}}}_{{{{\rm{FL}}}}}=\frac{1}{N}\sum\limits_{k=1}^{N}\sum\limits_{i=1}^{13}-{y}_{k,i}{\alpha }_{i}{(1-{p}_{k,i})}^{\gamma }\log ({p}_{k,i})-(1-{y}_{k,i})(1-{\alpha }_{i}){({p}_{k,i})}^{\gamma }\log (1-{p}_{k,i}),$$

where *N* was the batch size (that is, *N* = 128), and other parameters and variables were as defined. The focusing parameter *γ* was set to 2, which had been reported to work well in most of the experiments in the original paper^76^. Moreover, *α*_*i*_ ∈ [0, 1] was the balancing parameter that influenced the weights of positive and negative instances. It was set as the square of the complement of the fraction of samples labeled as 1, varying for each *i* due to the differing level of class imbalance across diagnostic categories (Table 1). The FL term did not take inter-class relationships into account. To address these relationships in our overall loss function, we also incorporated the RL term that induced loss if the sigmoid outputs for diagnostic categories labeled as 0 were not lower than those labeled as 1 by a predefined margin of *ϵ*, for any training sample *k*. We defined the RL term for any pair of diagnostic categories *i* and *j*, as follows:

$${{{{\mathcal{L}}}}}_{{{{\rm{RL}}}}}^{(i,\,j)}({{{{\bf{p}}}}}_{k},{{{{\bf{y}}}}}_{k})=\max (0,(\,{p}_{k,i}-{p}_{k,\,j})(\,{y}_{k,\,j}-{y}_{k,i})+\epsilon ),$$

Overall, the RL term was:

$${{{{\mathcal{L}}}}}_{{{{\rm{RL}}}}}=\frac{1}{N}\sum\limits_{k=1}^{N}\sum\limits_{i=1}^{13}\sum\limits_{j=i+1}^{13}{{{{\mathcal{L}}}}}_{{{{\rm{RL}}}}}^{(i,\,j)}({{{{\bf{p}}}}}_{k},{{{{\bf{y}}}}}_{k}).$$

Combining all terms, our overall loss function (${{{\mathcal{L}}}}$) was:

$${{{\mathcal{L}}}}={{{{\mathcal{L}}}}}_{{{{\rm{FL}}}}}+\lambda {{{{\mathcal{L}}}}}_{{{{\rm{RL}}}}}+\beta \parallel {{{\bf{w}}}}{\parallel }^{2},$$

where *λ* and *β* were the weights that controlled the importance of ${{{{\mathcal{L}}}}}_{{{{\rm{RL}}}}}$ and the L2 regularization terms, respectively. The training was done using the mini-batch strategy with the AdamW optimizer^77^, an improved version of the Adam optimizer^78^, with a learning rate of 0.001 for a total of 256 epochs. Additionally, we utilized a cosine learning rate scheduler with warm restarts^79^, initiating the first restart after 64 epochs and extending the restart period by a factor of 2 for each subsequent restart. The values of *ϵ*, *λ*, and *β* were determined to be *ϵ* = 0.25, *λ* = 0.005, and *β* = 0.0005, respectively, based on an evaluation of the overall model performance on the validation set. During training, the model performance was evaluated on the validation set at the end of each epoch, and the model with the highest performance was selected. To demonstrate the effectiveness of the focal loss in compensating for the high class imbalance, the performance of our baseline model was compared against that of a model trained without the focal loss term across all the 13 diagnostic categories (Table S16).

### Interpretability analysis

The primary goal of interpretability analysis is to demystify ML models by providing clear insights into how various features influence predictions. Central to this field lies the Shapley value^51^, originally a game theory concept, now repurposed to evaluate feature significance in ML models. In this context, each instance is considered a unique ‘game’, where features act as players contributing to the outcome. The model’s output is analogous to the game’s payoff, with the Shapley value quantifying each feature’s contribution towards this outcome. However, calculating Shapley values for all possible feature combinations is often computationally infeasible due to the sheer number of features. To overcome this, we applied permutation sampling to approximate Shapley values^80^, which simplifies computations while maintaining accuracy in estimating feature contributions. We performed Shapley analysis on the NC, MCI and dementia predictions within the NACC test set. We first identified cases for which the model yielded logit values greater than 0. We then selected a subset of 500 cases with the most features available per diagnostic group. Features were subsequently ranked based on their mean Shapley values. To account for data missingness, features that were absent for a case were assigned a zero Shapley value, ensuring their influence was accurately represented. The resulting distribution of Shapley values across features provided insight into their relative importance, with higher values indicating more influence.

### Traditional ML models

To assess our model’s ability to classify NC, MCI and dementia cases, we compared its performance with that of the CatBoost model, a tree-based classification framework^39,50^. Given the variable availability of features across the test cohorts (Tables S2, S4 and S5), we divided the data into two feature subsets. This stratification enabled a comparison with CatBoost, offering insights into our model’s performance using a range of parameters. The first feature subset consisted of variables common across all cohorts, including demographics, MMSE and Boston Naming Test scores. The second subset expanded on this by incorporating additional neuropsychological measures found in the NACC and ADNI cohorts, such as trail making tests A and B, logical memory IIA delayed recall, MoCA scores, and digit span forward and backward tests. We trained separate CatBoost models for each feature set but applied our model to both subsets without retraining, allowing for a consistent evaluation across different feature configurations.

### Biomarker validation

The predicted probabilities of the model for various etiologies were cross-validated with established gold-standard biomarkers pertinent to each respective etiology. Both the NACC and ADNI test cohorts were used in AD biomarker analyses, whereas only NACC testing data were used for FTD and LBD analyses due to biomarker availability. In the NACC dataset, binary UDS variables were used to define positivity for amyloid *β* (A*β*), tau and fluorodeoxyglucose F18 (FDG) PET biomarkers for AD due to varying PET processing methods across centers. Binary UDS variables were also used to define FDG and MRI evidence for FTD, and DaTscan as evidence for LBD. In ADNI, the University of California, Berkeley (UCB) A*β* PET processing pipeline yields Freesurfer-defined cortical summary and reference regions, as well as centiloids (CL). A cutoff value of 20 CL was chosen to define positivity^81^. For tau, the UCB processing pipeline yields standardized uptake value ratios (SUVr) in Freesurfer-defined regions. A meta-temporal region of interest was constructed following established standards^82^. A Gaussian mixture model with two components identified 1.74 SUVr as the optimal threshold to separate the two distributions, where values greater than 1.74 indicated tau PET positivity. Finally, the UCB FDG PET processing pipeline yields a meta-region of interest, on which a Gaussian mixture model with two components identified 1.21 SUVr as the best threshold, with values smaller than 1.21 indicating positivity for neurodegeneration. Information regarding the PET processing protocols can be found in the summaries of UCB amyloid, tau, and FDG PET methods available on the LONI Image Data Archive website^83^.

### Neuropathologic validation

The model’s predictive capacity for various dementia etiologies was substantiated through alignment with neuropathological evaluations sourced from the NACC, FHS and ADNI cohorts (Table S12). We included participants who conformed to the study’s inclusion criteria, had a diagnosis close to 3 years before death, and for whom neuropathological data were available. Standardization of data was conducted in accordance with the Neuropathology Data Form Version 10 protocols from the National Institute on Aging^84^. We pinpointed neuropathological indicators that influence the pathological signature of some dementia etiologies, such as arteriolosclerosis, the presence of neurofibrillary tangles and amyloid plaques, and CAA. These indicators were chosen to reflect the complex pathological terrain that defines each form of dementia. To examine the Thal phase for amyloid plaques (A score), subjects were categorized into two groups: one encompassing Phase 0, indicative of no amyloid plaque presence, and a composite group merging Phases 1-5, reflecting varying degrees of amyloid pathology. The model’s predictive performance was then compared across these groupings. For the Braak stage of neurofibrillary degeneration (B score), we consolidated stages I-VI into a single collective, representing the presence of AD-type neurofibrillary pathology, whereas stage 0 was designated for cases devoid of AD-type neurofibrillary degeneration. With respect to the density of neocortical neuritic plaques, assessed by the (CERAD or C score), individuals without neuritic plaques constituted one group, whereas those with any manifestation of neuritic plaques (sparse, moderate or frequent (C1–C3)) were aggregated into a separate group for comparative analysis of the model’s predictive outcomes. To evaluate model alignment with the severity of CAA, subjects were classified into two groups, one representing the absence of CAA and another encapsulating all stages of CAA severity, ranging from mild to severe. We also evaluated the presence of arteriolosclerosis, underscoring the role of vascular pathology in the progression of AD by decreasing cerebral blood flow and impairing A*β* clearance. Furthermore, to evaluate the model’s concordance with non-AD pathologies, we analyzed the association between the model-generated probabilities of VD with the presence of old microinfarcts and arteriolosclerosis, and FTD with the presence of TDP-43 pathology.

### AI-augmented clinician assessments

We aimed to ascertain if our model could bolster the diagnostic prowess of clinicians specializing in dementia care and diagnosis. To this end, a group of 12 neurologists and 7 neuroradiologists were invited to participate in diagnostic tasks on a subset of NACC cases (see ‘Data processing and training strategy’). Neurologists were presented with 100 cases, which included 15 cases each of NC and MCI, and 7 cases for each of the dementia etiologies. The data encompassed person-level demographics, medical history, social history, neuropsychological tests, functional assessments, and multisequence MRI scans where possible (that is, T1w, T2w, FLAIR, DWI and SWI sequences). They were asked to provide their diagnostic impressions, as well as a confidence score ranging from 0 to 100 for the diagnosis of each of the 13 labels. These confidence scores quantitatively reflect the clinician’s certainty in their diagnosis, with higher scores indicating greater certainty. This scoring system facilitated a quantitative comparison between the clinicians’ diagnostic certainty and the predictive probabilities generated by our model. Similarly, neuroradiologists were provided with the same multisequence MRI scans, along with information on age, gender, race, and education status from 70 clinically diagnosed dementia cases. They were also tasked with providing diagnostic impressions, as well as confidence scores concerning the origin of dementia (Glossary 1). To evaluate the potential enhancement of clinical judgments by our model, we calculated AI-augmented confidence scores by averaging the clinicians’ confidence scores with our model’s predicted probabilities. We then assessed the diagnostic accuracy of the clinicians’ original and AI-augmented confidence scores using AUROC and AUPR metrics. The specifics of the case samples and questionnaires provided to the neurologists and neuroradiologists are detailed below.

### Neurologist approach to the ratings

#### Neurologist 1

The clinical data were reviewed initially, taking note of potential contributors such as extreme age or education (for example, age > 90 years, education less than 9 grades), primary language and language of cognitive testing. Pertinent factors like a history of transient ischemic attack or stroke, PD diagnosis and/or PD medication usage, known genetic mutations, closed head injury, alcohol or substance use disorders, chronic psychiatric symptoms/disorders and APOE genotype were assessed. Next, the current level of functional abilities was evaluated from the provided initial description (for example, independent living, requiring assistance with some or all activities) and FAQ responses. FAQ scores of 9 or higher typically indicated limitations with instrumental activities of daily living, supporting a dementia diagnosis. FAQ scores ranging from 4 to 8 would align with MCI if cognitive test scores indicated cognitive decline. Subsequently, cognitive test scores were reviewed, with focus on age, education, and gender-adjusted Z scores. For those with NC, no Z scores deviated by 1 standard deviation below the mean (that is, no score of −1.0 or worse). Persons with MCI would exhibit at least one Z score of −1.5 or worse (for example, −1.75) or two scores of −1.0 in the same cognitive domain. Persons with dementia would typically present with two or more scores at −2.0 or worse. Interpretation for patients with very low education or non-native language cognitive testing was approached cautiously. Following this, brain MRIs (T1w images) were reviewed for signs of atrophy, the pattern of atrophy, and cerebrovascular disease. When available, DWI was used to identify a diffusion restriction pattern commonly seen in prion diseases. Functional abilities and cognitive test scores were used to classify persons as normal, MCI, or dementia. For persons between categories, a continuum scale was employed. For instance, a score of 80 for MCI and 20 for dementia would indicate an 80% likelihood of classification as MCI and a 20% likelihood of classification as dementia. For individuals with MCI or dementia, the most likely diagnostic category or categories were selected. In cases of mixed dementia or unclear causation, multiple diagnostic categories were chosen, with their scores summing to 100. Each category’s score reflected the estimated contribution and, for mixed dementias, the extent of their contribution. For example, a score of 70 for AD, 20 for LBD and 10 for VD would signify an estimated 70% contribution from AD, 20% from LBD and 10% from cerebrovascular disease.

#### Neurologist 2

The evaluation of case reports began with a comprehensive analysis of demographics, available medical history, APOE4 status, structured family history and an assessment of the patient’s level of functional independence. Subsequently, a thorough examination of corresponding clinical scales and neuropsychological test results was conducted. Careful observations were made regarding the subject’s educational background, the presence of visual or hearing impairments, and whether the tests were conducted in the subject’s native language. Following this, the synthesis of clinical data allowed for the prediction of the presence of MCI, dementia, or cognitive states falling below the MCI threshold, often referred to as ‘normal’ cognition. These predictions were quantified, with the most probable diagnosis assigned a rating exceeding 50%, whereas the others received lower ratings, reflecting the confidence in the diagnosis. Subsequently, the MRI sequences were examined alongside the case report to identify factors contributing to the patient’s clinical condition. Distinctly, findings such as medial temporal atrophy and parietal atrophy were prominently associated with AD, whereas the presence of flair hyperintensity and focal encephalomalacia without an alternative cause was considered indicative of vascular burden and/or dementia, especially when accompanied by deep and/or brainstem microhemorrhages. Brainstem atrophy was frequently observed in cases suggestive of potential stroke or Lewy body conditions, and the use of DWI sequences allowed for the potential identification of conditions like prion disease and epilepsy-related disorders. In assessing the clinical significance of these contributors, the most plausible factors were rated highest, whereas other contributors received lower but still considerable ratings, typically exceeding 50%. However, distinguishing psychiatric features stemming from a neurodegenerative process from those arising as independent comorbid issues occasionally posed a challenge. Importantly, observed vascular burden in imaging, even when it didn’t independently warrant a dementia diagnosis, was consistently acknowledged under the vascular category, often rated highly due to the confidence in its clinical significance.

#### Neurologist 3

In the approach to differential diagnosis for dementia, a detailed case overview encompassed a wide spectrum of clinical information including demographics, vitals and comprehensive personal and medical histories, alongside results from systematic physical, neurological, psychiatric and neurocognitive evaluations. Cognitive function was assessed using clinician impressions from neuropsychiatric evaluations and standardized testing with MMSE or MoCA, facilitating the distinction among NC, MCI and dementia. Functional assessments provided insights into the impact of neurological disorders on daily living activities. Specific scales and questionnaires, such as the Hachinski Ischemic Score, evaluations for PSP, and CBS, the Unified Parkinson’s Disease Rating Scale and the Neuropsychiatric Inventory Questionnaire, were instrumental in identifying localized or generalized neurological deficits, signs and symptoms of PD and related conditions, and characteristic features of LBD, such as visual hallucinations. The presence of typical symptoms for disorders like NPH also contributed to fine-tuning the differential diagnosis. The Geriatric Depression Scale was used to discern if primary psychiatric disorders might mimic dementia presentations. An extensive review of neurocognitive testing data aided in differentiating AD from other cognitive disorders. Detailed MRI analyses, revealing anomalies such as cortical atrophy, ischemic changes and ventriculomegaly, further refined the diagnostic process.

#### Neurologist 4

The patient’s cognitive status, ranging from NC to MCI or dementia, was primarily determined based on neuropsychiatric test results and the functional assessment scale. Special consideration was given to patients with Parkinson’s syndrome, as their movement disorders could impact functional assessment scores. When neuropsychiatric testing clearly indicated dementia, diagnosis was straightforward. However, cases teetering on the borderline between MCI and AD required a closer examination, where functional assessment scores, medical history, and physical examination findings were collectively considered, factoring in the influence of motor disorders on the assessment. This process involved adjusting the probability estimate based on clinical judgment. Regarding etiological diagnosis, a comprehensive evaluation was carried out, taking into account both medical history and imaging data. Cases presenting with Parkinson’s symptoms led to differential diagnoses that included PD dementia, dementia with Lewy bodies, CBD, PSP and others. In instances where imaging revealed markers of cerebral small vessel disease, the possibility of VD was explored. Notably, when prominent mental symptoms were coupled with atrophy in one side of the frontal and temporal lobes, consideration was given to frontotemporal degeneration. Infectious, metabolic, traumatic, and hereditary causes were also taken into account, guided by the relevant medical history. The adjustment of probability in these cases was guided by personal judgment.

#### Neurologist 5

The assessment combined insights from clinical and medication history, specific neurological examinations and neuropsychological test scores. Initially, attention was given to basic demographic data, such as age and the subject’s living situation. Subsequently, a comprehensive evaluation of medical and social history was conducted, considering potential dementia risk factors and relevant habits. The presence or absence of APOE alleles was noted. Medication history was scrutinized, particularly medications associated with vascular comorbidities like antihypertensives and anticoagulants, indicative of vascular disease risk. The presence of antidepressants was acknowledged, considering potential psychiatric conditions linked to cognitive decline. During the review of neurological examinations, focus was placed on gaze, tremor, parkinsonism and gait assessment. Neuropsychological examination scores were analyzed, first taking note of the number of abnormal tests. MoCA scores were used when available, alongside other tests like WMS. Language assessment, often relying on Animals and Digit span backwards, played a crucial role. Z scores and absolute scores were considered for test abnormality determination. Cognitive decline characterized by language and memory loss pointed to AD. The presence of hallucinations and parkinsonism suggested LBD, or if PD was advanced, it pointed to PD dementia. Executive dysfunction and disinhibition were signs of FTD. Hydrocephalus-associated urinary symptoms and specific findings hinted at NPH. MCI was identified through mildly abnormal tests and preserved daily activities. MRIs were considered, yet clinical synopsis took precedence when imaging findings did not align with the clinical scenario. In offering a final diagnosis, a single label was assigned in cases of diagnosis confidence, whereas multiple labels were used if overlapping symptoms or psychiatric comorbidities/alcoholism could obscure the presentation. In such scenarios, several labels were assigned with varying confidence levels. For instance, in equivocal cases of dementia and MCI, ratings were employed to determine the likelihood of each diagnosis. If both MCI and dementia were considered, dropdowns for each dementia subtype were used to indicate the more probable dementia type. When distinguishing between dementia and psychiatric conditions or acute encephalopathy proved challenging, all relevant options were marked alongside dementia.

#### Neurologist 6

In assessing clinical cases for dementia, the process began with a comprehensive review of key demographic and historical data, encompassing details like age, gender, educational background, family history, and existing medical comorbidities, to provide context for interpreting the cognitive presentation. The clinical records were systematically examined, with a specific focus on the critical domains relevant to diagnosing dementia syndromes. Key tools for initial assessment, such as the MMSE and the MoCA scores, provided an initial screening of the severity and pattern of cognitive impairment. Very low scores indicated advanced dementia, whereas higher scores within the mild impairment range prompted a more detailed review of neuropsychological test data. This battery of neurocognitive tests revealed the specific profile of cognitive deficits within domains such as memory, language, executive function, and visuospatial abilities, each of which hinted at potential etiologies. A fundamental component of the diagnostic process involved evaluating for any concurrent neurological signs, which entailed a meticulous examination of physical findings, with a particular focus on motor exam results, including assessments for rigidity, tremors, and gait disorders often associated with Parkinsonian disorders. Additionally, the Hachinski Ischemic Scale score was considered for insights into potential vascular contributions. Furthermore, it was imperative to observe the individual’s functional status and any neuropsychiatric symptoms, as they bore diagnostic and prognostic significance. The clinician had to ascertain whether the deficits impeded daily activities. Behavioral manifestations such as depression, hallucinations, delusions and agitation could provide critical distinctions between various dementia types. Once these key components were systematically reviewed, the clinician synthesized the data to formulate a comprehensive differential diagnosis. Cognitive testing profiles, behavioral presentation, family history, age of onset, and the presence of neurological signs were all weighed and considered in a holistic manner. Common differentials in dementia assessment included AD, vascular cognitive impairment, dementia with Lewy bodies, PD dementia and FTD. Lastly, the MRI results were scrutinized for any uncommon findings that could either support or contradict the differential diagnosis. This involved assessing major structural abnormalities or alterations, such as hydrocephalus or severe atrophy, which could provide further backing for the final diagnosis.

#### Neurologist 7

The interpretation method followed a structured approach. Initially, cognitive impairment severity (NC, MCI or dementia) was determined by assessing Functional Assessment Scale Score, independence level and neuropsychiatric testing. This assessment incorporated past medical history to exclude other potential causes of functional limitations. Etiology assessment comprised several considerations. VD was diagnosed when factors such as stroke history, cerebrovascular disease risk factors, focal neurological deficits, Hachinski infarction score, and specific MRI findings indicating infarctions, white matter hyperintensities, and perivascular spaces were present. Parkinsonism, as evaluated by the Unified Parkinson’s Disease Rating Scale, prompted investigation for LBD, NPH, VD, FTD and variants. LBD was considered for cases with visual hallucinations, Parkinsonism, cognitive impairment, and unremarkable MRI findings, whereas NPH diagnosis hinged on ventricular dilation and radiological features. FTD identification relied on executive function deficits, abnormal behavior, language impairment, and MRI-documented frontal/temporal lobe atrophy. Mental illness was contemplated for individuals with relevant medical history and substantial neuropsychiatric inventory and GDS symptoms. Prion disease recognition was based on distinctive MRI patterns. Conditions like infectious, metabolic, substance abuse, delirium, and psychiatric disorders were considered through medical history, coupled with the absence of specific MRI abnormalities. Lastly, multiple system atrophy was diagnosed in cases displaying Parkinson’s symptoms, defecation issues, ataxia and cerebellar atrophy on MRI, whereas TBI diagnosis was associated with head trauma history, cognitive decline, localized lesions, and secondary atrophy.

#### Neurologist 8

The evaluation process initiated with a comprehensive assessment of patient demographics, medical/family history, and risk factors. Cardiovascular and cerebrovascular risk factors were scrutinized due to their potential contribution to VD and vascular parkinsonism. Special attention was given to assessing activities of daily living (ADLs), which served as a crucial factor in distinguishing dementia from MCI. APOE status played a pivotal role in gauging the likelihood of AD. The presence of APOE4 heightened the risk of AD, particularly in early onset cases, whereas APOE2 could potentially serve as a protective factor. Psychiatric history was examined to identify behavioral changes and assess whether conditions like depression or anxiety contributed to cognitive symptoms. The GDS helped differentiate between pseudodementia/depression and other psychiatric illnesses affecting cognitive function. This information was crucial in pinpointing specific cognitive disorders (for example, PD dementia, behavioral variant FTD, impulse control disorders in the context of dopamine agonists). A meticulous examination of clinical findings focused on gait, tremor, and bradykinesia. The presence of rest tremor, bradykinesia, or rigidity prompted consideration of parkinsonism, or other forms of parkinsonism such as dementia with Lewy bodies (DLB), PSP or FTD. Comprehensive neuropsychological battery results were analyzed to discern patterns of cognitive impairment, differentiating between executive function deficits and memory impairments. Deviations in tasks such as Trails suggested executive dysfunction, potentially indicating subcortical dementia like DLB, PDD, VD or vascular parkinsonism. Poor performance on WAIS-R or WAIS-III indicated memory impairment, typically associated with cortical dementias like AD. Imaging studies were instrumental in the evaluation. Patterns like diffuse or parietal atrophy suggested AD, whereas frontal-temporal atrophy indicated FTD. The presence of widespread white matter disease (WMD) burden aligned with VD or vascular parkinsonism. Specific assessments included the evaluation of the swallow tail sign, associated with PD, and midbrain atrophy, assessed through sagittal images using the midbrain-to-pons ratio (midbrain area/pontine area). Regarding the rating system, no cases received a perfect score of 100, as most presented with mixed pathologies, combining features such as amyloid beta AD changes and alpha-synuclein aggregates with parkinsonism or alpha-synuclein alongside evidence of tauopathy in PD-PSP variants. Ratings between 50% and 80% indicated varying degrees of likelihood for a specific pathology, with ratings above 80% signifying a stronger likelihood of the disease or pathology being present.

#### Neurologist 9

The assessment began with a thorough review of the individual’s medical history, with a focus on identifying major diagnoses that could impact cognition. This included conditions like TBI, psychiatric disorders, stroke-related issues, and APOE status. Subsequently, the individual’s medication history was analyzed, considering potential biases introduced by medications commonly used for AD or PD, which might have implied a higher likelihood of these conditions. Functional status assessment followed, encompassing ADLs and instrumental activities of daily living (iADL), providing insights into the individual’s everyday capabilities. A comprehensive physical examination was conducted, emphasizing the identification of notable abnormalities that could offer insights into cognitive status. Psychiatric and cognitive testing scales were administered, and the results were carefully analyzed for consistency and coherence. These results were also cross-referenced with the person’s reported functional status. In cases of discrepancy, consideration was given to underlying mood or psychiatric disorders that may have influenced information accuracy. Chronology of symptoms, often absent from person-level histories, was evaluated with a particular focus on the Neuropsychiatric Inventory Questionnaire, which inquired about symptoms experienced within the last 30 days. During the review of imaging studies, the gathered information was taken into account. Attention was paid to imaging findings that may have indicated AD or vascular disease. Unusual symptoms in the person-level history, such as new motor problems or agitation, prompted consideration of rare conditions like FTD, Huntington’s disease, or Creutzfeldt-Jakob disease. Subsequently, a detailed review of the imaging data was conducted to identify specific features that could be indicative of these particular disorders. Lastly, the interpretation of cognitive testing scale results was influenced by the individual’s functional status. This guided the determination of whether the person exhibited signs of dementia or MCI or fell within the spectrum of normal cognitive function. The aim was to construct a comprehensive assessment of the individual’s cognitive state, accounting for these factors.

#### Neurologist 10

The determination of cognitive status, including NC, MCI or dementia, relied primarily on neuropsychiatric test outcomes and the functional assessment scale. Notably, when individuals exhibited Parkinsonism, functional abilities were often influenced by motor impairments, making neuropsychiatric test results more influential than the Functional Activities Questionnaire (FAQ). Given the absence of distinct cutoff points for these categories, adjustments to the probability assessment were made based on individual judgment. Regarding the etiological diagnosis, a comprehensive evaluation incorporated all available clinical information and imaging data. For instance, cases presenting with Parkinsonism prompted a focused differential diagnosis that considered conditions like DLB, characterized by symptoms such as parkinsonism, dementia and hallucinations. Others included PD dementia (PDD), typically occurring after a prolonged history of PD, vascular injuries with attention to severe small vessel disease, especially within the basal ganglia, and NPH, identified by enlarged brain ventricles. Conditions such as CBD and PSP, though less common, required the presence of more typical symptoms like apraxia in CBD or abnormal vertical eye movement in PSP for diagnosis. For individuals diagnosed with MCI or dementia but without Parkinsonism, the differential diagnosis primarily encompassed AD, FTD and vascular injuries. FTD, for example, might exhibit pronounced non-memory impairments, along with psychiatric and behavioral symptoms, and asymmetrical brain atrophy in frontal and/or temporal lobes. Additionally, vascular injuries played a substantial role in cognitive impairment and sometimes coexisted with AD pathology. In these instances, probability assessments were adjusted based on clinical judgment. For the remaining etiologies, establishing a diagnosis necessitated a detailed clinical history.

#### Neurologist 11

The evaluation process initiated with an assessment of the provided case profiles, encompassing baseline information like age, education, language, and required assistance. Supplementary data, including genetic test results such as APOE4 status, medication records, and relevant details, were also considered. Subsequently, various cognitive and physical examinations, along with associated indices, were reviewed to detect neurocognitive dysfunction. From these comprehensive case profiles, preliminary hypotheses were formulated to guide the diagnostic process, ultimately leading to specific diagnoses or a set of potential options. A meticulous evaluation of imaging studies for each case followed, examining different sequences and views for signs of cerebral atrophy or structural changes, including WMD. These imaging findings were correlated with case profile hypotheses to generate a list of probable diagnoses. Probability ratings were assigned to these diagnoses, reflecting the likelihood of their presence. The rating process initially involved determining whether cases met criteria for NC, MCI or dementia. In ambiguous cases distinguishing between dementia and MCI, probability ratings were provided for both, especially when the differentiation between MCI and mild dementia was uncertain based on testing outcomes. Subsequently, probable contributing factors to the diagnoses were identified by selecting the types of dementia most likely present. Many cases presented with multiple potential contributing causes, often including VD alongside AD. Quantifying the likelihood of each diagnosis involved assigning scores of 70 or higher to those with a high probability, regardless of an individual factor’s relatively low contribution to their dementia. Higher scores indicated a greater likelihood of that diagnosis being the primary cause. Causes with similar probabilities scores did not reflect an equal degree of causality to the individual’s condition but merely reflected an equal probability of occurrence. Scores ranging from 20 to 30 suggested the presence of dementia, though with a minor role in the clinical presentation. Scores below 10 indicated a very low probability, implying little to no significance.

#### Neurologist 12

While reviewing clinical data in conjunction with MRI scans, a notable absence was observed regarding information on symptom onset and progression. This critical aspect of history-taking has the potential to offer valuable insights into the diagnosis, as the pace of progression varies among different forms of dementia. For diagnostic purposes, reliance was placed on MMSE scores, employing a cutoff of 24 to diagnose dementia. Functional capacity assessments assisted in distinguishing between MCI and dementia. Psychiatric questionnaires proved useful in orienting toward specific diagnoses, such as Parkinson’s dementia, DLB or infectious causes. The evaluation of depression’s role in cognition was challenging, but the Geriatric Depression Scale provided some guidance. In cases of uncertainty, the MRI findings played a pivotal role. For instance, clear frontotemporal atrophy with behavioral disturbances and language involvement suggested FTD, whereas temporal lobe atrophy leaned more toward AD. In cases of DLB or Parkinson’s dementia, clinical presentation bore more weight when MRI results were unremarkable. Moderate to severe white matter abnormalities pointed to VD. In most cases, a shortlist of potential diagnoses was compiled before reviewing the MRI. However, there were instances where MRI results were conclusive and prompted a change in the diagnosis. For example, one case indicated possible Creutzfeldt-Jakob disease due to hallucinations and corresponding MRI findings. In another, an MRI revealed encephalomalacia with ventricular enlargement following a head injury. A young case with a cavum septum pellucidum was attributed to chronic traumatic encephalopathy. Lastly, global atrophy in an individual with a history of alcohol abuse and seizures pointed to alcoholic dementia. Providing a percentage of certainty for each diagnosis proved beneficial, as many cases presented mixed pathology, especially in Parkinson’s dementia, where vascular disease often contributed to the clinical picture.

### Neuroradiologist approach to the ratings

#### Neuroradiologist 1

The evaluation of MRI scans initiated with a global perspective to exclude multiple infarcts and identify notable brain atrophy patterns. The presence and severity of white matter lesions, chronic infarcts and microhemorrhages were recorded. Subsequent assessment focused primarily on volume loss, particularly emphasizing hemispheric asymmetry. The initial evaluation determined whether dominant frontal and anterior temporal or parietal and medial temporal volume loss was evident. A more detailed sub-analysis of each region was conducted, focusing on grading severity and documenting regional and focal volume loss in real time. The lobar volume loss evaluation was done systematically, starting with the frontal lobes, including attention to asymmetry when present. Sub-analyses of specific regions within the frontal lobes were conducted, such as the anterior insula, cingulate gyrus, precentral gyrus, and caudate nucleus. Evaluation of temporal lobe volume loss was also carried out, distinguishing mesial and non-mesial temporal lobe atrophy. Subanalyses of hippocampal, amygdala and parahippocampal atrophy were included, with special attention to anterior, lateral, and posterior temporal lobe atrophy, including fusiform, middle, and inferior temporal gyrus volume. The assessment for atrophy was extended to parietal and occipital lobe, documenting brainstem and cerebellar atrophy. When appraising ventricular size, a comparison was made relative to sulcal size. Findings favoring an AD pattern included the presence of predominant parietal and medial temporal lobe atrophy, or less frontal lobe involvement than parietal and temporal lobes. Deviations from the AD pattern, such as predominant frontal, anterior temporal, or occipital involvement, enlarged ventricles, or multiple infarcts, supported non-AD dementia patterns, including those indicative of LBD, VD, prion disease, FTD and its variants, NPH, TBI, psychiatric diagnoses and/or other conditions. A rating scale from 0 to 100 was used to assess the likelihood of various diagnostic considerations. A rating of 0 was selected when no evidence supported a particular diagnosis, whereas a rating of 100 indicated the imaging strongly suggested that entity. Ratings of 50 were assigned when imaging findings were equally likely to represent the entity in question.

#### Neuroradiologist 2

The approach to rating the cases followed a systematic checklist, starting with an assessment of the entire brain, then moving through various lobes: frontal, temporal, parietal, occipital and the brainstem. Within this framework, the aim was to determine the possible causes of dementia based on imaging findings. Initially, features indicative of NPH were sought. These features typically stood out from other conditions and included disproportionate ventricular enlargement, an acute callosal angle at the posterior commissure level, sulcal crowding near the vertex, and Sylvian fissure enlargement. Next, the focus shifted to assessing the overall burden of WMD, characterized by T2 FLAIR hyperintensities. Examination was carried out in regions with encephalomalacia or gliosis, which might signify prior infarcts, helping establish a potential vascular component to dementia, either as the sole cause or a contributing factor alongside other processes. Further examination was directed toward atrophy patterns, aiming to identify specific neurodegenerative processes. Disproportionate atrophy in the medial, basal, and lateral temporal lobes and the medial parietal lobes suggested AD. Relative preservation of medial temporal lobe structures hinted at dementia with Lewy bodies or PD dementia, although the absence of clinical history posed challenges for this diagnosis, as clinical features and typical MRI findings of medial temporal lobe preservation are valuable in a clinical setting. For FTD and its variants, the search was for frontal and/or temporal atrophy, predominately left posterior perisylvian or parietal atrophy, anterior temporal atrophy, predominant left posterior fronto-insular atrophy, midbrain atrophy relative to the pons (‘hummingbird’ sign), concavity of the dorsolateral midbrain, thinning of the tectal plate, or T2 hyperintense rim along the putamen with patchy or confluent T2 FLAIR hyperintensity in the rolandic subcortical white matter. In the quest for Prion disease indicators, examination included cortical/gyriform diffusion hyperintensity, often accompanied by thalamic and basal ganglia diffusion hyperintensity. Also explored were signs of encephalomalacia and gliosis typical of prior TBI.

#### Neuroradiologist 3

During case reviews, emphasis was placed on patient age and MRI findings as essential factors guiding the diagnostic process. Age served as a key determinant, informing the assessment of volume loss, particularly relevant in cases of AD and frontotemporal lobar degeneration (FTD). Each MRI sequence contributed uniquely to diagnostic considerations: T1w images held importance in gauging volume loss, discerning distinctive patterns within the hippocampus, temporal lobes, and parietal lobes for AD, and focusing on volume loss within the frontal and temporal lobes for FTD. In the assessment for NPH, attention was drawn to ventriculomegaly and its proportionality to volume loss. T1w images were also instrumental in identifying cerebellar atrophy, indicative of conditions like alcoholism or phenytoin use for seizures. Diffusion-weighted images played a critical role in detecting signs of Creutzfeldt-Jakob disease, characterized by hyperintensity in regions such as the insula, cingulate gyrus, frontal gyri, medial thalami, and possibly the basal ganglia. This sequence was also valuable for identifying infarcts. T2/FLAIR and other T2w images were essential for assessing small vessel disease burden, aiding in the evaluation of VD. They were also instrumental in detecting potential evidence of infectious, inflammatory, metabolic, or drug-related hyperintensity. The susceptibility-weighted images were used to assess for microhemorrhages, which could be associated with AD or Lewy body disease. Psychiatric diseases were typically exempt from numerical ratings as their diagnosis could not usually be ascertained through imaging. Ratings spanned from 70 to 90 in cases where a single diagnosis was highly confident. In scenarios where multiple potential diagnoses were considered, ratings ranged from 40 to 70 for each disease state, reflecting the estimated likelihood of each condition.

#### Neuroradiologist 4

Each case was approached by first reviewing the demographic information; however, as the project progressed, the demographic data became less informative, and by the midpoint of the project, demographics were reviewed only as a later step. The images were assessed using the SLICER software. The T2w and FLAIR sequences were carefully evaluated to gauge the extent of small vessel disease and infarcts, serving as indicators of potential vascular causes of cognitive impairment. These sequences also proved valuable for the exclusion of infectious, inflammatory, or toxic causes. The DWI sequence was employed to identify acute infarcts and to investigate neurodegenerative conditions such as Creutzfeldt-Jakob disease or fatal familial insomnia. Susceptibility-weighted images were analyzed to identify microhemorrhages, assess their extent and location, and rule out other potential causes of cognitive decline. However, the most pivotal sequences were the volumetric sequences acquired in all three anatomical planes. They were instrumental in assessing global or lobar-specific volume loss. Specific regions of interest included the hippocampal volume assessed through coronal sequences to rule out AD, the precuneus evaluated via sagittal sequences, and the parietal lobes examined in axial sequences. If frontal lobe volume loss was evident, then the temporal lobes were assessed for signs of FTD. Cerebellar volume loss or infratentorial volume loss led to considerations of alcohol abuse or phenytoin use, or cerebellar ataxias, whereas brainstem involvement indicated potential multisystem atrophy. Disproportionate ventricular dilatation raised suspicions of NPH. The rating scale used was comprehensive, and in cases where complete information was lacking, the diagnosis was assigned to the best of the ability. A diagnosis was rated as 100 when highly confident, and as 50 when uncertainty existed. Additionally, some cases were assigned a probability score between 50 and 100 when confident in excluding other potential causes, based on the imaging data.

#### Neuroradiologist 5

The approach to MR exams began with an evaluation of axial T2/FLAIR images, if available. If multiple regions of gliosis were observed alongside areas of encephalomalacia, resulting from prior infarctions in multiple vascular territories, consideration was given to the possibility of multi-infarct dementia. Moreover, when encephalomalacia and gliosis predominantly affected the temporal lobes, cerebral autosomal dominant arteriopathy with subcortical infarcts and leukoencephalopathy became a potential inclusion in the diagnostic considerations. Following the FLAIR sequence, assessment of diffusion-weighted images, if accessible, primarily served to rule out more acute conditions like Creutzfeldt-Jakob disease, herpes encephalitis, or other forms of encephalitis. Subsequently, T1w images were reviewed, preferably in 3D format, to examine ventricle and sulci dimensions. The presence of ventriculomegaly and sulcal crowding at the vertex prompted consideration of NPH as a potential diagnosis. Additionally, gyri were evaluated to identify areas exhibiting volume loss. T2w images were especially helpful in this regard, as they enhanced the visibility of CSF and accentuated regions of atrophy. Once the order of diagnostic differentials was established, a diagnostic rating was assigned. In this rating system, a score of 100 indicated absolute certainty, an exceedingly rare occurrence in radiology. Conversely, a score of less than 20 signified extreme unlikelihood, 25 denoted unlikeliness, 50 implied the possibility of the diagnosis, whereas a range of 50 to 75 indicated a probable diagnosis. Finally, a score exceeding 75 suggested a high likelihood of the diagnosis being accurate.

#### Neuroradiologist 6

The review process began with an examination of the provided individual-level demographics for each case. Subsequently, all images provided for each case underwent analysis using the SLICER software. T2/FLAIR sequence was the basis for assessing small vessel changes, subacute to chronic infarcts, encephalomalacia from TBI, and any areas displaying signal abnormalities indicative of potential alternative causes, such as neurodegenerative, infectious-inflammatory, or toxic-metabolic etiologies. T2/FLAIR sequence was also employed to investigate seizure-related changes. T2w images played a key role in evaluating ventricular size, examining the posterior fossa for small infarcts, and observing major intracranial arterial flow voids. Diffusion-weighted images were used to identify acute infarcts and regions with reduced diffusivity, potentially linked to other neurodegenerative, infectious-inflammatory, toxic-metabolic conditions, or seizure-related changes. Susceptibility-weighted images were utilized to detect areas featuring parenchymal microhemorrhage or calcification. Lastly, high-resolution T1w images were employed to analyze regional volume loss patterns suggestive of specific neurodegenerative processes. The evaluation process included the completion of the online ADRD radiologist task survey. During the assessment of sections regarding regional predominate atrophy, the high-resolution T1w images were revisited to ensure response accuracy. In the final section, person-level demographics and imaging findings were synthesized to arrive at the best-guess probability for each diagnosis. The rating scale corresponded to the likelihood of the best-guess diagnosis. For instance, if there was high confidence that a case represented a particular diagnosis, it was assigned a score of 100, with a score of 0 given to all other diagnoses. In cases of diagnostic uncertainty, where the estimated probability was 50%, a score of 50 was assigned.

#### Neuroradiologist 7

Brain volume loss was assessed based on age-appropriate norms, with T1 and T2/FLAIR sequences aiding in the evaluation of volume loss within each lobe. These sequences were particularly useful for assessing CSF presence near the convexity. Brainstem volume loss was primarily evaluated through mid-sagittal and axial images, which allowed for the examination of the pontine belly and cerebral peduncle size, respectively. Coronal images provided insights into hippocampal volume, determined by the prominence of the temporal horns of the lateral ventricle. Sagittal images were used to assess cerebellar volume loss. FLAIR sequences played a crucial role in detecting encephalomalacia, gliosis, infarcts and white matter changes. Distinct patterns were observed in various dementia types, such as parieto-temporal volume loss favoring AD. Extensive white matter changes with or without microhemorrhages in individuals over 60 years pointed to VD. White matter changes in younger individuals raised consideration of alternative causes like infections or metabolic factors. Alcohol use often correlated with cerebellar volume loss. Traumatic brain injury was suspected in cases with FLAIR signal changes and peripheral volume loss in the anterior temporal and inferior frontal lobes, with or without susceptibility, along with corpus callosum and brainstem findings, suggestive of diffuse axonal injury. Frontal and temporal lobe volume loss indicated FTD. The ‘hummingbird’ sign on sagittal images led to consideration of PSP, particularly when combined with brainstem volume loss. Asymmetric ventricular prominence relative to cortical volume loss hinted at NPH, with the corpus callosal angle measured on coronal images to confirm the diagnosis. Although no specific findings were linked to psychiatric disorders, the presence of a cavum septum pellucidum was weakly correlated. Multiple findings in a case, such as global volume loss, extensive white matter changes and microhemorrhages, leaned toward VD over AD due to the subjective nature of volume loss assessment. A higher rating was assigned to the diagnosis with more MRI findings supporting it, though no case received a perfect score of 100, with ratings exceeding 80 indicating a dominant diagnosis.

### Statistical analysis

We used one-way analysis of variance and the two-sided *χ*^2^ test for continuous and categorical variables, respectively to assess the overall differences in the population characteristics between the diagnostic groups across the study cohorts. We used the two-sample two-sided KS test for goodness of fit to compare model-predicted AD probabilities, *P*(*AD*), between MCI cases with an etiological diagnosis of AD and MCI cases without one. We applied the Kruskal-Wallis H-test for independent samples and subsequently conducted post-hoc Dunn’s testing with Bonferroni correction to evaluate the relationship between CDR scores and the model-predicted probabilities. In order to assess whether the model’s predicted probabilities for AD, FTD and LBD were higher for their respective biomarker positive cases compared to biomarker-negative ones, a one-sided Mann-Whitney U test was conducted. ADNI’s A*β* groups did not significantly deviate from normality and were therefore compared using the one-sided independent samples t-test. We applied the one-sided Mann-Whitney U test between neuropathologic scores and the model-predicted probabilities. To compare model predictions with expert-driven assessments, we used the Brunner-Munzel test to identify statistically significant increases in the mean disease probability scores between the levels of scoring categories. The Brunner-Munzel test was also used to compare the expert and model confidence scores for the true negative and true positive cases for each etiology. To evaluate the interrater reliability of label-specific confidence scores, we performed pairwise Pearson correlation analyses between clinicians’ scores and those generated by the model^85^. We calculated the average correlation coefficient across pairs and determined its 95% confidence interval. In addition, we estimated the mean Pearson correlation coefficient between the confidence score of neurologists and the model’s score for each diagnostic label using a bootstrapping approach. Pairwise statistical comparisons of AI-augmented clinician diagnostic performance (AUROC and AUPR) and clinicians only diagnostic performance were performed with the one-sided Wilcoxon signed-rank test. In all analyses, we opted for non-parametric tests when the Shapiro-Wilk test indicated significant deviations from normality. All statistical analyses were conducted at a significance level of 0.05.

### Performance metrics

We generated ROC and PR curves from predictions on both the NACC test data and other datasets. From each ROC and PR curve, we further derived the area under the curve values (AUC and AUPR, respectively). Further, we computed micro-, macro- and weighted-average AUC and AUPR values. Of note, the microaverage approach consolidates true positives, true negatives, false positives, and false negatives from all classes into a unified curve, providing a global performance metric. In contrast, the macroaverage calculates individual ROC/PR curves for each class before computing their unweighted mean, disregarding potential class imbalances. The weighted-average, whereas similar in approach to macroaveraging, assigns a weight to each class’s ROC/PR curve proportionate to its representation in the dataset, thereby acknowledging class prevalence. We also evaluated the model’s accuracy, sensitivity, specificity and Matthews correlation coefficient, with the latter being a balanced measure of quality for classes of varying sizes in a binary classifier. Performance metrics were initially calculated for the entire testing cohort, followed by a stratified analysis based on age, gender and race subgroups.

### Computational hardware and software

All MRI and non-imaging data were processed on a workstation equipped with an Intel i9 14-core 3.3 GHz processor and 4 NVIDIA RTX 2080Ti GPUs. Our software development utilized Python (version 3.11.7) and the models were developed using PyTorch (version 2.1.0). We used several other Python libraries to support data analysis, including pandas (version 1.5.3), scipy (version 1.10.1), tensorboardX (version 2.6.2), torchvision (version 0.15), and scikit-learn (version 1.2.2). Training the model on a single Quadro RTX8000 GPU on a shared computing cluster had an average runtime of 7 minutes per epoch, whereas the inference task took less than a minute per instance. All clinicians reviewed MRIs using 3D Slicer (version 4.10.2) and logged their findings in REDCap (version 11.1.3). Figures were prepared using Canva and Adobe Illustrator.

### Reporting summary

Further information on research design is available in the Nature Portfolio Reporting Summary linked to this article.

## Online content

Any methods, additional references, Nature Portfolio reporting summaries, source data, extended data, supplementary information, acknowledgements, peer review information; details of author contributions and competing interests; and statements of data and code availability are available at 10.1038/s41591-024-03118-z.

## Supplementary information

Supplementary Information Supplementary Tables 1–18, Supplementary Figures 1–6, Neurologists’ Approach to the Ratings, Neuroradiologists’ Approach to the Ratings.

Reporting Summary

## Data Availability

Data from ADNI, AIBL, NIFD, PPMI and 4RTNI can be downloaded from the LONI website at https://ida.loni.usc.edu. The ADNI Tau PET data used for biomarker validation in Fig. 4 correspond to the November 2021 version, and the amyloid PET data correspond to the June 2023 version. NACC and OASIS data can be downloaded at https://naccdata.org and https://sites.wustl.edu/oasisbrains/, respectively. Data from FHS (https://www.framinghamheartstudy.org/fhs-for-researchers/data-available-overview/) can be obtained by contacting fhs@bu.edu and conditions for access include the successful completion of all steps outlined at https://www.framinghamheartstudy.org/fhs-for-researchers/, as well as approval from the FHS Research Committee. LBDSU data can be requested by contacting the Stanford Alzheimer’s Disease Research Center at adrcstanford@stanford.edu and is subject to institutional approval. We used the Montreal Neuroimaging Institute MNI152 template for image processing purposes, and the template can be downloaded at http://www.bic.mni.mcgill.ca/ServicesAtlases/ICBM152NLin2009. All data used in this study should be available free of charge upon request from the specific cohorts.

## Code availability

Python scripts as well as help files along with information on the study population are made available on GitHub (https://github.com/vkola-lab/nmed2024).
