# Supplementary material for: AI-based differential diagnosis of dementia etiologies on multimodal data
Source: Nat Med. 2024 Jul 4;30(10):2977–89. doi: 10.1038/s41591-024-03118-z (PMC11485262; doi:10.1038/s41591-024-03118-z)
Supplement: Supplementary file 1 — Supplementary Tables 1–18, Supplementary Figures 1–6, Neurologists’ Approach to the Ratings, Neuroradiologists’ Approach to the Ratings. [file 41591_2024_3118_MOESM1_ESM.pdf]

nature medicine

Supplementary information

https://doi.org/10.1038/s41591-024-03118-z

AI-based differential diagnosis of dementia etiologies on multimodal data

In the format provided by the authors and unedited

**Dataset (group)**	**MMSE mean ± std**	**MOCA mean ± std**	**APOE N, % positive**
NACC			
NC [n = 17242]	28.76 ± 1.57ˆ	26.28 ± 2.7ˆ	3852, 30.07%ˆ
MCI [n = 7582]	26.94 ± 2.59ˆ	22.61 ± 3.53ˆ	1913, 38.2%ˆ
AD [n = 16131]	18.94 ± 6.79ˆ	15.15 ± 5.78ˆ	6840, 56.05%ˆ
LBD [n = 1913]	19.7 ± 7.07ˆ	16.13 ± 5.95ˆ	600, 44.02%ˆ
VD [n = 1919]	19.56 ± 6.85ˆ	16.19 ± 5.6ˆ	577, 42.06%ˆ
PRD [n = 114]	13.37 ± 10.03ˆ	16.18 ± 7.6ˆ	6, 23.08%ˆ
FTD [n = 2898]	19.09 ± 8.13ˆ	17.06 ± 6.66ˆ	740, 31.97%ˆ
NPH [n = 138]	20.54 ± 6.46ˆ	17.38 ± 5.1ˆ	38, 42.7%ˆ
SEF [n = 808]	20.63 ± 6.33ˆ	17.21 ± 5.46ˆ	225, 39.4%ˆ
PSY [n = 2700]	20.33 ± 6.52ˆ	16.15 ± 5.83ˆ	911, 46.13%ˆ
TBI [n = 265]	21.07 ± 6.29ˆ	17.24 ± 6.82ˆ	74, 39.15%ˆ
ODE [n = 1234]	20.32 ± 6.99ˆ	17.11 ± 5.89ˆ	403, 42.38%ˆ
p-value	<1.0e-200	<1.0e-200	<1.0e-200
NIFD			
NC [n = 124]	29.35 ± 0.76	27.58 ± 1.53ˆ	N.A.
FTD [n = 129]	24.75 ± 4.54ˆ	19.69 ± 5.72ˆ	N.A.
p-value	1.961e-23	2.645e-16	N.A.
PPMI			
NC [n = 171]	N.A.	27.51 ± 2.37ˆ	N.A.
MCI [n = 27]	N.A.	24.69 ± 3.27ˆ	N.A.
p-value	N.A.	3.004e-07	N.A.
AIBL			
NC [n = 480]	28.7 ± 1.24	N.A.	12, 2.5%
MCI [n = 102]	27.1 ± 2.08	N.A.	12, 11.76%
AD [n = 79]	20.42 ± 5.46	N.A.	14, 17.72%
p-value	4.585e-121	N.A.	8.951e-09
OASIS			
NC [n = 424]	28.99 ± 1.25ˆ	N.A.	140, 33.02%
MCI [n = 27]	28.15 ± 1.67	N.A.	11, 40.74%
AD [n = 32]	23.91 ± 5.05	N.A.	20, 62.5%
LBD [n = 4]	25.5 ± 2.69	N.A.	2, 50.0%
FTD [n = 4]	18.33 ± 8.26ˆ	N.A.	4, 100.0%
p-value	4.439e-50	N.A.	4.510e-05
LBDSU			
NC [n = 134]	N.A.	27.43 ± 2.23ˆ	N.A.
MCI [n = 35]	N.A.	24.0 ± 3.14	N.A.
LBD [n = 13]	N.A.	16.69 ± 4.75	N.A.
p-value	N.A.	2.231e-30	N.A.
4RTNI			
NC [n = 12]	27.2 ± 2.4ˆ	24.2 ± 2.44ˆ	N.A.
MCI [n = 31]	26.1 ± 3.95ˆ	21.19 ± 4.83ˆ	N.A.
FTD [n = 37]	21.49 ± 7.2ˆ	17.14 ± 7.4ˆ	N.A.
p-value	1.657e-03	3.605e-03	N.A.
ADNI			
NC [n = 868]	29.09 ± 1.12	25.97 ± 2.65ˆ	138, 29.61%ˆ
MCI [n = 1119]	27.64 ± 1.85	23.07 ± 3.25ˆ	438, 47.2%ˆ
AD [n = 417]	23.19 ± 2.23	16.93 ± 4.53ˆ	229, 64.33%ˆ
p-value	<1.0e-200	3.984e-199	3.117e-22
FHS			
NC [n = 394]	26.05 ± 3.36ˆ	N.A.	N.A.
MCI [n = 434]	25.2 ± 3.4ˆ	N.A.	N.A.
AD [n = 687]	21.63 ± 5.08ˆ	N.A.	N.A.
LBD [n = 73]	22.91 ± 4.16ˆ	N.A.	N.A.
VD [n = 113]	22.41 ± 5.44ˆ	N.A.	N.A.
FTD [n = 8]	20.33 ± 3.4ˆ	N.A.	N.A.
p-value	3.132e-26	N.A.	N.A.

Table S1: **Study population.** Nine independent datasets were used for this study, including ADNI, NACC, NIFD, PPMI, OASIS, LBDSU, 4RTNI, and FHS. Data from NACC, NIFD, PPMI, OASIS, LBDSU, and 4RTNI were used for model training. Data from ADNI, FHS, and a held-out set from NACC were used for model testing. The p-value for each dataset indicates the statistical significance of inter-group differences per column. We used one-way ANOVA and two-sided χ^2 tests for continuous and categorical variables, respectively. Please refer to Glossary 1 for more information on the acronyms. N.A. denotes not available. The symbol ˆ indicates that data was not available for some subjects.

Cohort	Features
NACC	• Primary reason for visit<br>• Principal referral source<br>• subject's month of birth<br>• subject's year of birth<br>• Hispanic/Latino ethnicity<br>• Hispanic origins<br>• Race<br>• Second race<br>• Third race<br>• Primary language<br>• Years of education<br>• Marital status<br>• Living situation<br>• Level of independence<br>• Type of residence<br>• Is the subject left- or right-handed?<br>• Subject's age at visit<br>• Derived NIH race definitions<br>• Indicator of first-degree family member with cognitive impairment<br>• Indicator of mother with cognitive impairment<br>• Indicator of father with cognitive impairment<br>• In this family, is there evidence of a dominantly inherited AD mutation?<br>• In this family, is there evidence for an AD mutation (from a list of specific mutations)?<br>• Source of evidence for AD mutation<br>• In this family, is there evidence for an FTLD mutation?<br>• In this family, is there evidence for an FTLD mutation (from a list of specific mutations)?<br>• Source of evidence for FTLD mutation<br>• In this family, is there evidence for a mutation other than an AD or FTLD mutation?<br>• Source of evidence for other mutation<br>• Smoked cigarettes in last 30 days<br>• Smoked more than 100 cigarettes in life<br>• Total years smoked cigarettes<br>• Average number of packs smoked per day<br>• If the subject quit smoking, the age at which he/she last smoked (i.e., quit)<br>• In the past three months, has the subject consumed any alcohol?<br>• During the past three months, how often did the subject have at least one drink of any alcoholic beverage such as wine, beer, malt liquor, or spirits?<br>• Heart attack/cardiac arrest<br>• More than one heart attack/cardiac arrest?<br>• Year of most recent heart attack<br>• Atrial fibrillation<br>• Angioplasty/endarterectomy/stent<br>• Cardiac bypass procedure<br>• Pacemaker and/or defibrillator<br>• Pacemaker<br>• Congestive heart failure<br>• Angina<br>• Heart valve replacement or repair<br>• Other cardiovascular disease<br>• Stroke<br>• More than one stroke reported as of the Initial Visit<br>• Most recently reported year of stroke as of the Initial Visit<br>• Transient ischemic attack (TIA)<br>• More than one TIA reported as of the Initial Visit<br>• Most recently reported year of TIA as of the Initial Visit<br>• Parkinson's disease (PD)<br>• Year of PD diagnosis<br>• Other Parkinsonian disorder<br>• Year of Parkinsonian disorder diagnosis<br>• Seizures<br>• Traumatic brain injury (TBI)<br>• Traumatic brain injury (TBI) with brief loss of consciousness<br>• brain trauma - brief unconsciousness<br>• TBI with extended loss of consciousness - 5 minutes or longer<br>• brain trauma - extended unconsciousness<br>• TBI without loss of consciousness - as might result from military detonations or sports injury<br>• brain trauma - chronic deficit<br>• Year of most recent TBI<br>• Other neurological condition<br>• Diabetes<br>• If Recent/active or Remote/inactive diabetes, which type?<br>• Hypertension<br>• Hypercholesterolemia<br>• Vitamin B12 deficiency<br>• Thyroid disease<br>• Arthritis<br>• Type of Arthritis<br>• Arthritis, region affected - spine<br>• Region affected - unknown<br>• Incontinence - urinary<br>• Incontinence - bowel<br>• Sleep apnea history reported at Initial Visit<br>• REM sleep behavior disorder (RBD) history reported at Initial Visit<br>• Hyposomnia/insomnia history reported at Initial Visit<br>• Other sleep disorder history reported at Initial Visit<br>• Alcohol abuse - clinically significant occurring over a 12-month period manifested in one of the following areas: work, driving, legal, or social<br>• Other abused substances - clinically significant impairment occurring over a 12-month period manifested in one of the following areas: work, driving, legal, or social<br>• Post-traumatic stress disorder (PTSD)<br>• bipolar disorder<br>• Schizophrenia<br>• Active depression in the last two years<br>• Depression episodes more than two years ago<br>• Anxiety<br>• Obsessive-compulsive disorder (OCD)<br>• Developmental neuropsychiatric disorders (e.g., autism spectrum disorder [ASD], attention-deficit hyperactivity disorder [ADHD], dyslexia)<br>• Other psychiatric disorder<br>• History of traumatic brain injury (TBI)<br>• Subject's sex<br>• Arthritis, region affected - upper extremity<br>• Arthritis, region affected - lower extremity<br>• NPI-Q co-participant<br>• Delusions severity<br>• Hallucinations severity<br>• Agitation or aggression severity<br>• Depression or dysphoria severity

Table S2

Cohort	Features
NACC	• Elation or euphoria severity<br>• Apathy or indifference severity<br>• Disinhibition severity<br>• Irritability or lability severity<br>• Motor disturbance severity<br>• Nighttime behaviors severity<br>• Appetite and eating severity<br>• Anxiety severity<br>• Number of APOE e4 alleles<br>• Were there abnormal neurological exam findings?<br>• Are focal deficits present indicative of central nervous system disorder?<br>• Is gait disorder present indicative of central nervous system disorder?<br>• Are there eye movement abnormalities present indicative of central nervous system disorder?<br>• Parkinsonian signs<br>• Resting tremor - left arm<br>• Resting tremor - right arm<br>• Slowing of fine motor movements - left side<br>• Slowing of fine motor movements - right side<br>• Rigidity - left arm<br>• Rigidity - right arm<br>• bradykinesia<br>• Parkinsonian gait disorder<br>• Postural instability<br>• Neurological sign considered by the examiner to be most likely consistent with cerebrovascular disease<br>• Cortical cognitive deficit (e.g., aphasia, apraxia, neglect)<br>• Focal or other neurological findings consistent with SIVD (subcortical ischemic vascular dementia)<br>• Motor (may include weakness of combination of face, arm, and leg; reflex changes, etc.) - left side<br>• Motor (may include weakness of combination of face, arm, and leg; reflex changes, etc.) - right side<br>• Cortical visual field loss - left side<br>• Cortical visual field loss - right side<br>• Somatosensory loss - left side<br>• Somatosensory loss - right side<br>• Higher cortical visual problem suggesting posterior cortical atrophy (e.g., prosopagnosia, simultagnosia, Balint's syndrome) or apraxia of gaze<br>• Findings suggestive of progressive supranuclear palsy (PSP), corticobasal syndrome (CBS), or other related disorders<br>• Eye movement changes consistent with PSP<br>• Dysarthria consistent with PSP<br>• Axial rigidity consistent with PSP<br>• Gait disorder consistent with PSP<br>• Apraxia of speech<br>• Apraxia consistent with CBS - left side<br>• Apraxia consistent with CBS - right side<br>• Cortical sensory deficits consistent with CBS - left side<br>• Cortical sensory deficits consistent with CBS - right side<br>• Ataxia consistent with CBS - left side<br>• Ataxia consistent with CBS - right side<br>• Alien limb consistent with CBS - left side<br>• Alien limb consistent with CBS - right side<br>• Dystonia consistent with CBS, PSP, or related disorder - left side<br>• Dystonia consistent with CBS, PSP, or related disorder - right side<br>• Myoclonus consistent with CBS - left side<br>• Myoclonus consistent with CBS - right side<br>• Findings suggesting ALS (e.g., muscle wasting, fasciculations, upper motor and/or lower motor neuron signs)<br>• Normal pressure hydrocephalus - gait apraxia<br>• Other findings (e.g., cerebella ataxia, chorea, myoclonus)<br>• Were all findings unremarkable?<br>• Was any part of the MMSE completed?<br>• Administration of the MMSE was:<br>• Language of MMSE administration<br>• Subject was unable to complete one or more sections due to visual impairment<br>• Subject was unable to complete one or more sections due to hearing impairment<br>• Orientation subscale score - Time<br>• Orientation subscale score - Place<br>• Intersecting pentagon subscale score<br>• Total MMSE score (using D-L-R-O-W)<br>• The remainder of the battery was administered:<br>• Language of test administration<br>• If this test has been administered to the subject within the past 3 months, specify the date previously administered (month)<br>• If this test has been administered to the subject within the past 3 months, specify the date previously administered (day)<br>• If this test has been administered to the subject within the past 3 months, specify the date previously administered (year)<br>• Total score from the previous test administration<br>• Total number of story units recalled from this current test administration<br>• Logical Memory IIA - Delayed - Total number of story units recalled<br>• Logical Memory IIA - Delayed - Time elapsed since Logical Memory IA - Immediate<br>• Total score for copy of Benson figure<br>• Total score for 10- to 15-minute delayed drawing of Benson figure<br>• Recognized original stimulus from among four options<br>• Digit span forward trials correct<br>• Digit span forward length<br>• Digit span backward trials correct<br>• Digit span backward length<br>• Animals - Total number of animals named in 60 seconds<br>• Vegetable - Total number of vegetables named in 60 seconds<br>• Trail Making Test Part A - Total number of seconds to complete<br>• Part A - Number of commission errors<br>• Part A - Number of correct lines<br>• Trail Making Test Part B - Total number of seconds to complete<br>• Part B - Number of commission errors<br>• Part B - Number of correct lines<br>• WAIS-R Digit Symbol<br>• Boston Naming Test (30) - Total score<br>• Number of correct F-words generated in 1 minute<br>• Number of F-words repeated in 1 minute<br>• Number of non-F-words and rule violation errors in 1 minute<br>• Number of correct L-words generated in 1 minute<br>• Number of L-words repeated in 1 minute<br>• Number of non-L-words and rule violation errors in 1 minute<br>• Total number of correct F-words and L-words<br>• Total number of F-word and L-word repetition errors<br>• Total number of non-F/L-words and rule violation errors

Table S2

Cohort	Features
NACC	• Per clinician, based on the neuropsychological examination, the subject's cognitive status is deemed<br>• Modality of communication used to administer neuropsychological battery<br>• Was any part of MoCA administered?<br>• If no part of MoCA administered, reason code<br>• Where was MoCA administered?<br>• Language of MoCA administration<br>• Subject was unable to complete one or more sections due to visual impairment<br>• Subject was unable to complete one or more sections due to hearing impairment<br>• MoCA Total Raw Score - uncorrected<br>• MoCA Total Score - corrected for education<br>• MoCA: Visuospatial/executive - Trails<br>• MoCA: Visuospatial/executive - Cube<br>• MoCA: Visuospatial/executive - Clock contour<br>• MoCA: Visuospatial/executive - Clock numbers<br>• MoCA: Visuospatial/executive - Clock hands<br>• MoCA: Language - Naming<br>• MoCA: Memory - Registration (two trials)<br>• MoCA: Attention - Digits<br>• MoCA: Attention - Letter A<br>• MoCA: Attention - Serial 7s<br>• MoCA: Language - Repetition<br>• MoCA: Language - Fluency<br>• MoCA: Abstraction<br>• MoCA: Delayed recall - No cue<br>• MoCA: Delayed recall - Category cue<br>• MoCA: Delayed recall - Recognition<br>• MoCA: Orientation - Date<br>• MoCA: Orientation - Month<br>• MoCA: Orientation - Year<br>• MoCA: Orientation - Day<br>• MoCA: Orientation - Place<br>• MoCA: Orientation - City<br>• Craft Story 21 Recall (Immediate) - Total story units recalled, verbatim scoring<br>• Craft Story 21 Recall (Immediate) - Total story units recalled, paraphrase scoring<br>• Number Span Test: Forward - Number of correct trials<br>• Number Span Test: Forward - Longest span forward<br>• Number Span Test: backward - Number of correct trials<br>• Number Span Test: backward - Longest span backward<br>• Craft Story 21 Recall (Delayed) - Total story units recalled, verbatim scoring<br>• Craft Story 21 Recall (Delayed) - Total story units recalled, paraphrase scoring<br>• Craft Story 21 Recall (Delayed) - Delay time<br>• Craft Story 21 Recall (Delayed) - Cue (boy) needed<br>• Multilingual Naming Test (MINT) - Total score<br>• Multilingual Naming Test (MINT) - Total correct without semantic cue<br>• Multilingual Naming Test (MINT) - Semantic cues: Number given<br>• Multilingual Naming Test (MINT) - Semantic cues: Number correct with cue<br>• Multilingual Naming Test (MINT) - Phonemic cues: Number given<br>• Multilingual Naming Test (MINT) - Phonemic cues: Number correct with cue<br>• MoCA blind Total raw score - uncorrected<br>• MoCA-blind Total Score - corrected for education<br>• Rey Auditory Verbal Learning: Trial 1 total recall<br>• Rey Auditory Verbal Learning: Trial 1 intrusions<br>• Rey Auditory Verbal Learning: Trial 2 total recall<br>• Rey Auditory Verbal Learning: Trial 2 intrusions<br>• Rey Auditory Verbal Learning: Trial 3 total recall<br>• Rey Auditory Verbal Learning: Trial 3 intrusions<br>• Rey Auditory Verbal Learning: Trial 4 total recall<br>• Rey Auditory Verbal Learning: Trial 4 intrusions<br>• Rey Auditory Verbal Learning: Trial 5 total recall<br>• Rey Auditory Verbal Learning: Trial 5 intrusions<br>• Rey Auditory Verbal Learning: Trial 6 total recall<br>• Rey Auditory Verbal Learning: Trial 6 intrusions<br>• Oral Trail Making Test - Part A: Total number of seconds to complete<br>• Oral Trail Making Test - Part A: Number of commission errors<br>• Oral Trail Making Test - Part A: Number of correct lines<br>• Oral Trail Making Test Part B: Total number of seconds to complete<br>• Oral Trail Making Test Part B: Number of commission errors<br>• Oral Trail Making Test Part B: Number of correct lines<br>• Rey Auditory Verbal Learning: total delayed recall<br>• Rey Auditory Verbal Learning: delayed intrusions<br>• Rey Auditory Verbal Learning: recognition total correct<br>• Rey Auditory Verbal Learning: recognition total false positives<br>• Verbal naming test: total correct without a cue<br>• Verbal naming test: total correct with a phonemic cue<br>• How valid do you think the participant's responses are?<br>• What makes this participant's responses less valid? Hearing impairment<br>• What makes this participant's responses less valid? Distractions<br>• What makes this participant's responses less valid? Interruptions<br>• What makes this participant's responses less valid? Lack of effort or disinterest<br>• What makes this participant's responses less valid? Fatigue<br>• What makes this participant's responses less valid? Emotional issues<br>• What makes this participant's responses less valid? Unapproved assistance<br>• What makes this participant's responses less valid? Other<br>• In the past four weeks, did the subject have any difficulty or need help with: Writing checks, paying bills, or balancing a checkbook<br>• In the past four weeks, did the subject have any difficulty or need help with: Assembling tax records, business affairs, or other paper<br>• In the past four weeks, did the subject have any difficulty or need help with: Shopping alone for clothes, household necessities, or groceries<br>• In the past four weeks, did the subject have any difficulty or need help with: Playing a game of skill such as bridge or chess, working on a hobby<br>• In the past four weeks, did the subject have any difficulty or need help with: Heating water, making a cup of coffee, turning off the stove<br>• In the past four weeks, did the subject have any difficulty or need help with: Preparing a balanced meal<br>• In the past four weeks, did the subject have any difficulty or need help with: Keeping track of current events

Table S2

Cohort	Features
NACC	• In the past four weeks, did the subject have any difficulty or need help with: Paying attention to and understanding a TV program, book, or magazine<br>• In the past four weeks, did the subject have any difficulty or need help with: Remembering appointments, family occasions, holidays, medications<br>• In the past four weeks, did the subject have any difficulty or need help with: Traveling out of the neighborhood, driving, or arranging to take public transportation<br>• Is the subject able to complete the GDS, based on the clinician's best judgment?<br>• Are you basically satisfied with your life?<br>• Have you dropped many of your activities and interests?<br>• Do you feel that your life is empty?<br>• Do you often get bored?<br>• Are you in good spirits most of the time?<br>• Are you afraid that something bad is going to happen to you?<br>• Do you feel happy most of the time?<br>• Do you often feel helpless?<br>• Do you prefer to stay at home, rather than going out and doing new things?<br>• Do you feel you have more problems with memory than most?<br>• Do you think it is wonderful to be alive now?<br>• Do you feel pretty worthless the way you are now?<br>• Do you feel full of energy?<br>• Do you feel that your situation is hopeless?<br>• Do you think that most people are better off than you are?<br>• Total GDS Score<br>• Abrupt onset (re: cognitive status)<br>• Stepwise deterioration (re: cognitive status)<br>• Somatic complaints<br>• Emotional incontinence<br>• History or presence of hypertension<br>• History of stroke<br>• Focal neurological symptoms<br>• Focal neurological signs<br>• Hachinski ischemic score<br>• Cerebrovascular disease contributing to cognitive impairment<br>• Relationship between stroke and cognitive impairment<br>• Imaging evidence<br>• Single strategic infarct<br>• Multiple infarcts<br>• Extensive white matter hyperintensity<br>• Other imaging evidence<br>• Subject taking any medications<br>• Total number of medications reported at each visit<br>• Reported current use of any type of antihypertensive or blood pressure medication<br>• Reported current use of an antihypertensive combination therapy<br>• Reported current use of an angiotensin converting enzyme (ACE) inhibitor<br>• Reported current use of an antiadrenergic agent<br>• Reported current use of a beta-adrenergic blocking agent (beta-blocker)<br>• Reported current use of a calcium channel blocking agent<br>• Reported current use of a diuretic<br>• Reported current use of a vasodilator<br>• Reported current use of an angiotensin II inhibitor<br>• Reported current use of lipid lowering medication<br>• Reported current use of nonsteroidal anti-inflammatory medication<br>• Reported current use of an anticoagulant or antiplatelet agent<br>• Reported current use of an antidepressant<br>• Reported current use of an antipsychotic agent<br>• Reported current use of an FDA-approved medication for Alzheimer's disease symptoms<br>• Reported current use of an antiparkinson agent<br>• Reported current use of estrogen hormone therapy<br>• Reported current use of estrogen + progestin hormone therapy<br>• Reported current use of a diabetes medication<br>• Reported current use of an anxiolytic, sedative, or hypnotic agent<br>• UPDRS normal<br>• Speech<br>• Facial expression<br>• Tremor at rest - face, lips, chin<br>• Tremor at rest - right hand<br>• Tremor at rest - left hand<br>• Tremor at rest - right foot<br>• Tremor at rest - left foot<br>• Action or postural tremor - right hand<br>• Action or postural tremor - left hand<br>• Rigidity - neck<br>• Rigidity - right upper extremity<br>• Rigidity - left upper extremity<br>• Rigidity - right lower extremity<br>• Rigidity - left lower extremity<br>• Finger taps - right hand<br>• Finger taps - left hand<br>• Hand movements - right hand<br>• Hand movements - left hand<br>• Alternating movement - right hand<br>• Alternating movement - left hand<br>• Leg agility - right leg<br>• Leg agility - left leg<br>• Arising from chair<br>• Posture<br>• Gait<br>• Posture stability<br>• body bradykinesia and hypokinesia<br>• subject's height (inches)<br>• subject's weight (lbs)<br>• body mass index (BMI)<br>• Subject blood pressure (sitting), systolic<br>• Subject blood pressure (sitting), diastolic<br>• Subject resting heart rate (pulse)<br>• Without corrective lenses, is the subject's vision functionally normal?<br>• Does the subject usually wear corrective lenses?<br>• If the subject usually wears corrective lenses, is the subject's vision functionally normal with corrective lenses?<br>• Without a hearing aid(s), is the subject's hearing functionally normal?<br>• Does the subject usually wear a hearing aid(s)?<br>• If the subject usually wears a hearing aid(s), is the subject's hearing functionally normal with a hearing aid(s)?<br>• Imaging (MRI scans)

Table S2: **Features from the NACC cohort.** This table shows the list of all the features extracted from the NACC cohort, which were used for model training.

Cohort	Features
AIBL	• Subject's age at visit<br>• Subject's sex<br>• Number of APOE e4 alleles<br>• Total number of story units recalled from this current test administration<br>• Logical Memory IIA - Delayed - Total number of story units recalled<br>• Total MMSE score (using D-L-R-O-W)<br>• Imaging (MRI scans)
NIFD	• Subject's age at visit<br>• Subject's sex<br>• Derived NIH race definitions<br>• Years of education<br>• Digit span forward length<br>• Digit span backward length<br>• Animals - Total number of animals named in 60 seconds<br>• Total MMSE score (using D-L-R-O-W)<br>• MoCA Total Score - corrected for education<br>• Total GDS Score<br>• Imaging (MRI scans)
PPMI	• Subject's age at visit<br>• Subject's sex<br>• Derived NIH race definitions<br>• Years of education<br>• Hispanic/Latino ethnicity<br>• Trail Making Test Part A - Total number of seconds to complete<br>• Trail Making Test Part B - Total number of seconds to complete<br>• MoCA Total Score - corrected for education<br>• Imaging (MRI scans)
OASIS	• Subject's age at visit<br>• Subject's sex<br>• Years of education<br>• Hispanic/Latino ethnicity<br>• Derived NIH race definitions<br>• Total years smoked cigarettes<br>• Pacemaker<br>• Cardiac bypass procedure<br>• Heart attack/cardiac arrest<br>• Congestive heart failure<br>• Atrial fibrillation<br>• Transient ischemic attack (TIA)<br>• Angioplasty/endarterectomy/stent<br>• Other cardiovascular disease<br>• Hypercholesterolemia<br>• Alcohol abuse - clinically significant occurring over a 12-month period manifested in one of the following areas: work, driving, legal, or social<br>• Other abused substances - clinically significant impairment occurring over a 12-month period manifested in one of the following areas: work, driving, legal, or social<br>• brain trauma - chronic deficit<br>• brain trauma - extended unconsciousness<br>• brain trauma - brief unconsciousness<br>• Diabetes<br>• Thyroid disease<br>• Vitamin B12 deficiency<br>• Incontinence - bowel<br>• Active depression in the last two years<br>• Depression episodes more than two years ago<br>• Other psychiatric disorder<br>• Seizures<br>• Hypertension<br>• Stroke<br>• Smoked more than 100 cigarettes in life<br>• Average number of packs smoked per day<br>• Traumatic brain injury (TBI)<br>• Incontinence - urinary<br>• Agitation or aggression severity<br>• Motor disturbance severity<br>• Delusions severity<br>• Disinhibition severity<br>• Hallucinations severity<br>• Depression or dysphoria severity<br>• Nighttime behavior severity<br>• Apathy or indifference severity<br>• Elation or euphoria severity<br>• Anxiety severity<br>• Appetite and eating severity<br>• Irritability or lability severity<br>• Number of APOE e4 alleles<br>• Digit span forward trials correct<br>• Digit span backward trials correct<br>• Digit span forward length<br>• Digit span backward length<br>• Total MMSE score (using D-L-R-O-W)<br>• Trail making test Part A - Total number of seconds to complete<br>• Trail making test Part B - Total number of seconds to complete<br>• Logical memory IIA - Delayed - Total number of story units recalled<br>• Total number of story units recalled from this current test administration<br>• Animals - Total number of animals named in 60 seconds<br>• Boston naming test (30) - Total score<br>• Total GDS score<br>• In the past four weeks, did the subject have any difficulty or need help with: Assembling tax records, business affairs, or other paper<br>• In the past four weeks, did the subject have any difficulty or need help with: Shopping alone for clothes, household necessities, or groceries<br>• In the past four weeks, did the subject have any difficulty or need help with: Writing checks, paying bills, or balancing a checkbook<br>• In the past four weeks, did the subject have any difficulty or need help with: Heating water, making a cup of coffee, turning off the stove<br>• In the past four weeks, did the subject have any difficulty or need help with: Playing a game of skill such as bridge or chess, working on a hobby<br>• In the past four weeks, did the subject have any difficulty or need help with: Traveling out of the neighborhood, driving, or arranging to take public transportation<br>• In the past four weeks, did the subject have any difficulty or need help with: Paying attention to and understanding a TV program, book, or magazine<br>• In the past four weeks, did the subject have any difficulty or need help with: Preparing a balanced meal<br>• In the past four weeks, did the subject have any difficulty or need help with: Keeping track of current events<br>• In the past four weeks, did the subject have any difficulty or need help with: Remembering appointments, family occasions, holidays, medications<br>• Imaging (MRI scans)

Table S3: **Features from the AIBL, NIFD, PPMI, and OASIS cohorts.** This table provides a systematic enumeration of the variables extracted from the AIBL, NIFD, PPMI and OASIS cohorts, illustrating the range of features employed in our analytical model and emphasizing the breadth of the dataset compilation.

Cohort	Features	
LBDSU	• Subject's age at visit<br>• Subject's sex<br>• Derived NIH race definitions<br>• Years of education	• Hispanic/Latino ethnicity<br>• MoCA Total Score - corrected for education<br>• Imaging (MRI scans)
4RTNI	• Subject's sex<br>• Subject's age at visit<br>• Years of education<br>• Hispanic/Latino ethnicity<br>• Derived NIH race definitions<br>• Agitation or aggression severity<br>• Motor disturbance severity<br>• Delusions severity<br>• Disinhibition severity<br>• Hallucinations severity<br>• Depression or dysphoria severity<br>• Nighttime behavior severity<br>• Apathy or indifference severity	• Elation or euphoria severity<br>• Anxiety severity<br>• Appetite and eating severity<br>• Irritability or lability severity<br>• UPDRS normal<br>• Total MMSE score (using D-L-R-O-W)<br>• MoCA Total Score - corrected for education<br>• Trail making test Part A - Total number of seconds to complete<br>• Trail making test Part B - Total number of seconds to complete<br>• Part A - Number of correct lines<br>• Part B - Number of correct lines<br>• Total GDS Score<br>• Imaging (MRI scans)
FHS	• Subject's sex<br>• Subject's age at visit<br>• Hispanic/Latino ethnicity<br>• Race<br>• Derived NIH race definitions<br>• Marital status<br>• Left- or right-handedness<br>• subject's weight (lbs)<br>• subject's height (inches)<br>• Body mass index (BMI)	• Blood pressure (sitting), systolic<br>• Blood pressure (sitting), diastolic<br>• Smoked cigarettes in last 30 days<br>• Total MMSE score (using D-L-R-O-W)<br>• Boston naming test (30) - Total score<br>• History of stroke<br>• Reported current use of a diabetes medication<br>• Reported current use of lipid lowering medication<br>• Imaging (MRI scans)

Table S4: **Features from the LBDSU, 4RTNI and FHS cohorts.** This table enumerates the features collected from the LBDSU, 4RTNI, and FHS cohorts, illustrating the range of features employed in our analytical model and emphasizing the breadth of the dataset compilation. Of note, FHS was used as an external dataset to validate our model's predictive performance.

Cohort	Features
ADNI	• Subject's age at visit<br>• Subject's sex<br>• Years of education<br>• Hispanic/Latino ethnicity<br>• Derived NIH race definitions<br>• Primary language<br>• Marital status<br>• Type of residence<br>• Is the subject left- or right-handed?<br>• Indicator of mother with cognitive impairment<br>• Indicator of father with cognitive impairment<br>• Orientation subscale score - Time<br>• Orientation subscale score - Place<br>• Total years smoked cigarettes<br>• Heart attack/cardiac arrest<br>• Hypertension<br>• History or presence of hypertension<br>• Stroke<br>• History of stroke<br>• Focal neurological symptoms<br>• Focal neurological signs<br>• Hachinski ischemic score<br>• Subject's height (inches)<br>• Subject's weight (lbs)<br>• Subject blood pressure (sitting), systolic<br>• Subject blood pressure (sitting), diastolic<br>• Subject resting heart rate (pulse)<br>• Depression episodes more than two years ago<br>• Are you basically satisfied with your life?<br>• Have you dropped many of your activities and interests?<br>• Do you feel that your life is empty<br>• Do you often get bored?<br>• Are you in good spirits most of the time?<br>• Are you afraid that something bad is going to happen to you?<br>• Do you feel happy most of the time?<br>• Do you often feel helpless?<br>• Do you prefer to stay at home, rather than going out and doing new things?<br>• Do you feel you have more problems with memory than most?<br>• Do you think it is wonderful to be alive now?<br>• Do you feel pretty worthless the way you are now<br>• Do you feel full of energy?<br>• Do you feel that your situation is hopeless?<br>• Do you think that most people are better off than you are?<br>• Abrupt onset (re: cognitive status)<br>• Stepwise deterioration (re: cognitive status)<br>• Somatic complaints<br>• Emotional incontinence<br>• Other psychiatric disorder<br>• Indicator of first-degree family member with cognitive impairment<br>• Alcohol abuse - clinically significant occurring over a 12-month period manifested in one of the following areas: work, driving, legal, or social<br>• Agitation or aggression severity<br>• Motor disturbance severity<br>• Delusions severity<br>• Disinhibition severity<br>• Hallucinations severity<br>• Depression or dysphoria severity<br>• Nighttime behavior severity<br>• Apathy or indifference severity<br>• Elation or euphoria severity<br>• Anxiety severity<br>• Appetite and eating severity<br>• Irritability or lability severity<br>• Number of APOE e4 alleles<br>• Multilingual Naming Test (MINT) - Total score<br>• Digit span forward trials correct<br>• Digit span backward trials correct<br>• Digit span forward length<br>• Digit span backward length<br>• Total MMSE score (using D-L-R-O-W)<br>• Trail making test Part A - Total number of seconds to complete<br>• Trail making test Part B - Total number of seconds to complete<br>• Logical memory IIA - Delayed - Total number of story units recalled<br>• Total number of story units recalled from this current test administration<br>• Animals - Total number of animals named in 60 seconds<br>• MoCA Total Score - corrected for education<br>• MoCA: Visuospatial/executive - Trails<br>• MoCA: Visuospatial/executive - Cube<br>• MoCA: Visuospatial/executive - Clock contour<br>• MoCA: Visuospatial/executive - Clock numbers<br>• MoCA: Visuospatial/executive - Clock hands<br>• MoCA: Language - Naming<br>• MoCA: Attention - Digits<br>• MoCA: Attention - Letter A<br>• MoCA: Attention - Serial 7s<br>• MoCA: Language - Repetition<br>• MoCA: Language - Fluency<br>• MoCA: Abstraction<br>• MoCA: Delayed recall - No cue<br>• MoCA: Orientation - Date<br>• MoCA: Orientation - Month<br>• MoCA: Orientation - Year<br>• MoCA: Orientation - Day<br>• MoCA: Orientation - Place<br>• MoCA: Orientation - City<br>• Boston naming test (30) - Total score<br>• Total GDS score<br>• In the past four weeks, did the subject have any difficulty or need help with: Assembling tax records, business affairs, or other paper<br>• In the past four weeks, did the subject have any difficulty or need help with: Shopping alone for clothes, household necessities, or groceries<br>• In the past four weeks, did the subject have any difficulty or need help with: Writing checks, paying bills, or balancing a checkbook<br>• In the past four weeks, did the subject have any difficulty or need help with: Heating water, making a cup of coffee, turning off the stove<br>• In the past four weeks, did the subject have any difficulty or need help with: Playing a game of skill such as bridge or chess, working on a hobby<br>• In the past four weeks, did the subject have any difficulty or need help with: Traveling out of the neighborhood, driving, or arranging to take public transportation<br>• In the past four weeks, did the subject have any difficulty or need help with: Paying attention to and understanding a TV program, book, or magazine<br>• In the past four weeks, did the subject have any difficulty or need help with: Preparing a balanced meal<br>• In the past four weeks, did the subject have any difficulty or need help with: Keeping track of current events<br>• In the past four weeks, did the subject have any difficulty or need help with: Remembering appointments, family occasions, holidays, medications<br>• Imaging (MRI scans)

Table S5: **Features from the ADNI cohort.** This table shows the list of all the features extracted from the ADNI cohort, which were used for model testing.

Dataset (group)	T1	T2	FLAIR	SWI
NACC	1970	352	318	32
NIFD	633	414	537	3
PPMI	241	N.A.	N.A.	N.A.
AIBL	681	N.A.	334	N.A.
OASIS	662	N.A.	N.A.	N.A.
LBDSU	181	N.A.	N.A.	N.A.
4RTNI	165	119	120	N.A.
ADNI	1055	N.A.	N.A.	N.A.
FHS	115	109	114	N.A.

Table S6: **MRI sequences used for model development.** T1-weighted, T2-weighted, fluid attenuated inversion recovery and susceptibility weighted imaging were included from NACC, NIFD, PPMI, AIBL, OASIS, LDBSU and 4RTNI for model training. A portion of MRIs from the NACC dataset along with MRIs from ADNI, and FHS were reserved for model testing.

Dataset (group)	Balanced Accuracy	Precision	Sensitivity	Specificity	F1 Score	MCC	AUROC	AUPR
NACC								
NC	0.93	0.9	0.92	0.94	0.91	0.85	0.98	0.97
MCI	0.83	0.52	0.80	0.85	0.63	0.56	0.91	0.67
DE	0.94	0.92	0.94	0.93	0.93	0.87	0.99	0.98
AD	0.89	0.84	0.87	0.91	0.86	0.78	0.96	0.93
LBD	0.87	0.43	0.80	0.95	0.56	0.56	0.96	0.68
VD	0.83	0.28	0.74	0.92	0.41	0.42	0.93	0.47
PRD	0.67	0.09	0.35	0.99	0.14	0.17	0.96	0.12
FTD	0.89	0.36	0.90	0.89	0.51	0.53	0.96	0.67
NPH	0.55	0.12	0.11	1.00	0.12	0.12	0.91	0.077
SEF	0.66	0.069	0.42	0.90	0.12	0.13	0.82	0.064
PSY	0.79	0.24	0.71	0.86	0.36	0.36	0.90	0.36
TBI	0.62	0.07	0.26	0.98	0.11	0.12	0.90	0.098
ODE	0.68	0.11	0.46	0.89	0.17	0.18	0.84	0.11
ADNI								
NC	0.83	0.64	0.97	0.69	0.77	0.64	0.94	0.89
MCI	0.76	0.82	0.63	0.88	0.71	0.53	0.87	0.83
DE	0.90	0.64	0.91	0.89	0.75	0.71	0.97	0.88
AD	0.91	0.70	0.89	0.92	0.78	0.74	0.97	0.86
FHS								
NC	0.59	0.35	0.42	0.76	0.38	0.17	0.66	0.33
MCI	0.53	0.40	0.13	0.93	0.20	0.098	0.59	0.34
DE	0.68	0.68	0.68	0.68	0.68	0.36	0.73	0.71
AD	0.65	0.63	0.52	0.78	0.57	0.32	0.72	0.64
LBD	0.52	0.077	0.068	0.96	0.072	0.032	0.62	0.071
VD	0.65	0.18	0.44	0.85	0.26	0.20	0.74	0.30
FTD	0.59	0.016	0.25	0.92	0.03	0.045	0.71	0.028

Table S7: **Model performance.** This table presents the performance metrics of our model across the NACC, ADNI, and FHS datasets. Specifically, the results for the NACC testing dataset are based on the input features outlined in Table S2. For the ADNI and FHS datasets, the results are derived from a restricted set of input features detailed in Table S4, S5. Of note, these results are influenced by the use of a limited selection of input features. Despite this limitation, the model, which was initially trained on the NACC data incorporating a broader feature set, demonstrates the capability to generalize and make predictions on the ADNI and FHS datasets. This indicates the model's robustness and its potential to yield predictions even with significant missing input feature information, albeit with some reduction in performance. Demographic information for each cohort can be found in Tables 1 and S1.

Dataset (group)	Balanced Accuracy		Precision		Sensitivity		Specificity		F1 Score		MCC		AUROC		AUPR	
NACC	Our	CatBoost	Our	CatBoost	Our	CatBoost	Our	CatBoost	Our	CatBoost	Our	CatBoost	Our	CatBoost	Our	CatBoost
NC	0.71	0.74	0.53	0.60	0.87	0.80	0.55	0.69	0.66	0.69	0.41	0.47	0.79	0.84	0.69	0.74
MCI	0.53	0.52	0.34	0.38	0.11	0.05	0.96	0.98	0.17	0.09	0.11	0.09	0.66	0.71	0.26	0.30
DE	0.73	0.75	0.90	0.72	0.51	0.76	0.95	0.75	0.65	0.74	0.52	0.51	0.82	0.86	0.83	0.86
ADNI																
NC	0.66	0.70	0.45	0.55	0.99	0.75	0.33	0.66	0.62	0.63	0.37	0.39	0.81	0.77	0.62	0.59
MCI	0.53	0.56	0.68	0.58	0.11	0.32	0.95	0.79	0.20	0.42	0.13	0.14	0.64	0.63	0.59	0.55
DE	0.77	0.83	0.86	0.55	0.55	0.79	0.98	0.86	0.67	0.65	0.64	0.57	0.95	0.94	0.81	0.80
FHS																
NC	0.69	0.53	0.38	0.26	0.78	0.56	0.60	0.50	0.51	0.35	0.32	0.05	0.73	0.51	0.42	0.22
MCI	0.51	0.51	0.34	0.67	0.03	0.02	0.98	1.00	0.06	0.04	0.03	0.09	0.66	0.55	0.36	0.32
DE	0.65	0.57	0.73	0.58	0.48	0.55	0.82	0.59	0.58	0.56	0.32	0.15	0.74	0.59	0.73	0.65

(a)

Dataset (group)	Balanced Accuracy		Precision		Sensitivity		Specificity		F1 Score		MCC		AUROC		AUPR	
NACC	Our	CatBoost	Our	CatBoost	Our	CatBoost	Our	CatBoost	Our	CatBoost	Our	CatBoost	Our	CatBoost	Our	CatBoost
NC	0.84	0.87	0.69	0.79	0.92	0.87	0.75	0.86	0.79	0.83	0.65	0.72	0.93	0.94	0.88	0.90
MCI	0.65	0.59	0.35	0.45	0.48	0.23	0.82	0.94	0.41	0.31	0.27	0.24	0.75	0.79	0.33	0.39
DE	0.83	0.88	0.90	0.83	0.74	0.91	0.93	0.85	0.81	0.87	0.69	0.76	0.94	0.95	0.93	0.94
ADNI																
NC	0.76	0.74	0.54	0.67	0.99	0.66	0.53	0.82	0.70	0.66	0.52	0.47	0.91	0.87	0.84	0.78
MCI	0.67	0.62	0.75	0.62	0.49	0.51	0.86	0.73	0.59	0.56	0.38	0.25	0.79	0.67	0.73	0.56
DE	0.88	0.86	0.71	0.57	0.82	0.86	0.93	0.86	0.76	0.68	0.71	0.62	0.96	0.94	0.84	0.81
FHS																
NC	0.69	0.52	0.38	0.45	0.78	0.07	0.60	0.97	0.51	0.13	0.32	0.10	0.73	0.52	0.42	0.26
MCI	0.51	0.50	0.34	0.00	0.03	0.00	0.98	1.00	0.06	0.00	0.03	0.00	0.66	0.60	0.36	0.34
DE	0.65	0.52	0.73	0.51	0.48	0.98	0.82	0.06	0.58	0.67	0.32	0.12	0.74	0.60	0.73	0.65

(b)

Table S8: **Model performance comparison with CatBoost.** This table presents the performance comparison between our model and CatBoost across the NACC, ADNI, and FHS datasets on NC, MCI, DE using two different subsets of features. The first subset was composed of common demographics information, as well as MMSE and Boston Naming Test scores. The second subset created on the first subset by incorporating additional neuropsychological measures found in the NACC and ADNI cohorts, such as trail making tests A and B, logical memory IIA delayed recall, MoCA scores, and digit span forward and backward tests. The unavailable features in the ADNI and FHS dataset are imputed for the CatBoost model. (a) Results with the first subset. (b) Results with the second subset. These findings indicate that our model has better generalization capabilities compared to Catboost when applied to external cohorts.

Comparison group	KS 2samp statistic	p-value
MCI	0.09	4.29e-3
DE	0.57	$<$1e-200

Table S9: **Statistical analysis of model alignment with prodromal AD.** Two-sample two-sided Kolmogorov-Smirnov test for goodness of fit statistics for prodromal disease plots indicating statistical significance between cases with AD as an etiological factor compared to those without AD. This comparison was conducted for both the Mild Cognitive Impairment (MCI) group and the dementia group.

**Cohort**	**Kruskal statistic**	**p-value**	**N**
NACC	6921.71	<1.0e-200	8895
ADNI	1518.79	<1.0e-200	2400
FHS	292.04	3.84e-64	1651

(a) **Kruskal-Wallis H-test**

**Comparison**	**p-value**	**N**
**NACC**		
0.0, 0.5	<1.0e-200	5986
0.0, 1.0	<1.0e-200	4984
0.0, 2.0	<1.0e-200	3932
0.0, 3.0	<1.0e-200	3470
0.5, 1.0	<1.0e-200	4652
0.5, 2.0	<1.0e-200	3600
0.5, 3.0	1.36e-105	3138
1.0, 2.0	6.24e-19	2598
1.0, 3.0	3.79e-09	2136
2.0, 3.0	1.00	1084
**ADNI**		
0.0, 0.5	<1.0e-200	2181
0.0, 1.0	<1.0e-200	1090
0.5, 1.0	2.07e-47	1529
**FHS**		
0.0, 0.5	7.34e-01	860
0.0, 1.0	4.50e-48	1217
0.5, 1.0	1.74e-40	1225

(b) **Dunn-Bonferroni posthoc test**

Table S10: **Statistical analysis of model alignment with clinical dementia rating (CDR). a,** Significant Kruskal-Wallis H-test statistics observed in the CDR plots reveal substantial variability among the CDR groups. Lower p-values ($p < 0.0001$) indicate that there is a statistically significant difference in the distribution of dementia probabilities across at least two of the CDR groups. **b,** We performed Dunn-Bonferroni posthoc testing for detailed pairwise comparisons among the CDR groups within each cohort.

**Etiology**	**Cohort**	**Biomarker**	**Statistic**	**p-value**
AD	NACC	Aβ PET	10303.50†	2.04e-25
AD	NACC	Tau PET	935.50†	6.48e-8
AD	NACC	FDG PET	3730.0†	3.00e-15
AD	ADNI	Aβ PET	-12.06‡	9.74e-31
AD	ADNI	Tau PET	5857.50†	4.10e-27
AD	ADNI	FDG PET	14924.0†	5.66e-43
FTD	NACC	MR	30935.50†	1.52e-51
FTD	NACC	FDG PET	1599.50†	2.08e-13
PD	NACC	DaTScan	318.50†	6.26e-06

Table S11: **Statistical analysis of biomarker validation.** Statistics for biomarker validation plots indicating statistical significance between biomarker negative and positive groups across etiologies. † indicates a one-side Mann-Whitney U test; ‡ indicates a one-sided independent samples t-test.

Cohort	Age at death (years) mean ± std	Male gender (percentage)	CDR mean ± std
NACC			
AD [n = 131]	79.28 ± 10.61	74, 56.49%	1.44 ± 0.75
LBD [n = 44]	76.64 ± 8.31	36, 81.82%	1.44 ± 0.66
VD [n = 15]	83.73 ± 10.39	7, 46.67%	1.37 ± 0.86
PRD [n = 5]	562.6 ± 4.96	5, 100%	0.7 ± 0.24
FTD [n = 20]	67.15 ± 8.61	13, 65%	1.82 ± 0.98
NPH [n = 1]	77 ± 0.0	1, 100%	0.5 ± 0.0
SEF [n = 3]	73 ± 24.04	2, 66.67%	0.83 ± 0.23
TBI [n = 1]	87 ± 0.0	1, 100%	2.0 ± 0.0
ODE [n = 11]	68.82 ± 14.99	7, 63.64%	1.45 ± 0.86
p-value	1.428e-08	5.956e-02	1.471e-01
ADNI			
AD [n = 19]	82.05 ± 9.03	13, 68.42%	0.86 ± 0.22
FHS			
AD [n = 55]	91 ± 7.0	18, 32.72%	1.01 ± 0.15
LBD [n = 4]	92.75 ± 2.16	3, 75%	1.0 ± 0.0
VD [n = 5]	93 ± 3.35	4, 80%	1.2 ± 0.4
FTD [n = 2]	79 ± 4.0	1, 50%	1.0 ± 0.0
p-value	1.584e-01	8.227e-02	2.972e-01

Table S12: **Cases with post mortem findings used for model validation.** Model validation was conducted using cases with post-mortem findings from three independent datasets: ADNI, NACC, and FHS. Continuous variables were analyzed using one-way ANOVA, while categorical variables were assessed with χ^2 tests. The p-values derived for each dataset reflect the statistical significance of differences between groups for each column.

Etiology	Neuropath	Mann-Whitney U statistic	p-value
AD	A score	282.5	7.11e-05
AD	B score	571.5	6.07e-06
AD	C score	3916.5	1.73e-06
AD	Cerebral amyloid angiopathy	6938.5	0.01
AD	Arteriolosclerosis	2607.0	0.01
VD	Old microinfarcts	2289.5	0.0001
VD	Arteriolosclerosis	2085.5	0.0002
FTD	FTLD with TDP-43 pathology	252.0	0.0008

Table S13: **Statistical analysis of neuropathological validation.** *Mann-Whitney U* statistics and corresponding p-values for the neuropathological validation plots, which indicate statistical significance between dementia etiologies and neuropathological indicators.

Etiology	Rater	AUC median	AUC IQR	W	p-value
NC	Neurologist	0.930	0.034	0.0	4.88e-04
	AI-augmented Neurologist	0.980	0.011		
MCI	Neurologist	0.699	0.115	0.0	4.88e-04
	AI-augmented Neurologist	0.793	0.063		
DE	Neurologist	0.914	0.094	8.0	2.44e-04
	AI-augmented Neurologist	0.954	0.022		
AD	Neurologist	0.761	0.042	1.0	2.44e-04
	AI-augmented Neurologist	0.866	0.026		
LBD	Neurologist	0.833	0.080	1.0	2.44e-04
	AI-augmented Neurologist	0.949	0.025		
VD	Neurologist	0.613	0.125	0.0	2.44e-04
	AI-augmented Neurologist	0.846	0.043		
PRD	Neurologist	0.517	0.093	0.0	2.44e-04
	AI-augmented Neurologist	0.899	0.015		
FTD	Neurologist	0.708	0.106	0.0	2.44e-04
	AI-augmented Neurologist	0.895	0.043		
NPH	Neurologist	0.719	0.153	0.0	2.44e-04
	AI-augmented Neurologist	0.844	0.075		
SEF	Neurologist	0.517	0.140	0.0	2.44e-04
	AI-augmented Neurologist	0.704	0.075		
PSY	Neurologist	0.613	0.073	0.0	2.44e-04
	AI-augmented Neurologist	0.789	0.021		
TBI	Neurologist	0.497	0.098	0.0	2.44e-04
	AI-augmented Neurologist	0.875	0.023		
ODE	Neurologist	0.516	0.082	0.0	6.10e-03
	AI-augmented Neurologist	0.558	0.042		
AD	Radiologist	0.632	0.104	0.0	7.81e-03
	AI-augmented Radiologist	0.681	0.085		
LBD	Radiologist	0.490	0.068	0.0	7.81e-03
	AI-augmented Radiologist	0.598	0.087		
VD	Radiologist	0.657	0.057	0.0	7.81e-03
	AI-augmented Radiologist	0.730	0.035		
PRD	Radiologist	0.541	0.089	0.0	7.81e-03
	AI-augmented Radiologist	0.918	0.026		
FTD	Radiologist	0.734	0.126	0.0	7.81e-03
	AI-augmented Radiologist	0.826	0.081		
NPH	Radiologist	0.739	0.131	0.0	7.81e-03
	AI-augmented Radiologist	0.848	0.063		
SEF	Radiologist	0.454	0.163	0.0	7.81e-03
	AI-augmented Radiologist	0.495	0.133		
PSY	Radiologist	0.542	0.122	0.0	7.81e-03
	AI-augmented Radiologist	0.660	0.067		
TBI	Radiologist	0.492	0.075	27.0	9.92e-01
	AI-augmented Radiologist	0.459	0.032		
ODE	Radiologist	0.480	0.030	4.0	5.47e-02
	AI-augmented Radiologist	0.505	0.038		

Table S14: **Statistical analysis of AI-augmented clinician AUROCs.** Detailed statistics for individual clinicians' AUROC and AI-augmented clinicians' AUROC. Median consensus confidence score and interquartile range (IQR) are presented. One-tailed Wilcoxon signed-rank test was performed to test for increase in performance for each label without any corrections made for multiple comparisons, and the test W statistic and its associated p-value are presented.

Etiology	Rater	AUC median	AUC IQR	W	p-value
NC	Neurologist	0.790	0.098	1.0	4.88e-04
	AI-augmented Neurologist	0.911	0.023		
MCI	Neurologist	0.301	0.105	1.0	4.88e-04
	AI-augmented Neurologist	0.411	0.074		
DE	Neurologist	0.942	0.051	0.0	2.44e-04
	AI-augmented Neurologist	0.977	0.013		
AD	Neurologist	0.667	0.037	0.0	2.44e-04
	AI-augmented Neurologist	0.830	0.046		
LBD	Neurologist	0.439	0.174	0.0	2.44e-04
	AI-augmented Neurologist	0.740	0.127		
VD	Neurologist	0.225	0.088	0.0	2.44e-04
	AI-augmented Neurologist	0.451	0.084		
PRD	Neurologist	0.081	0.032	0.0	2.44e-04
	AI-augmented Neurologist	0.327	0.051		
FTD	Neurologist	0.344	0.170	0.0	2.44e-04
	AI-augmented Neurologist	0.574	0.238		
NPH	Neurologist	0.321	0.131	13.0	2.12e-02
	AI-augmented Neurologist	0.401	0.130		
SEF	Neurologist	0.145	0.113	0.0	2.44e-04
	AI-augmented Neurologist	0.254	0.112		
PSY	Neurologist	0.265	0.112	1.0	4.88e-04
	AI-augmented Neurologist	0.452	0.054		
TBI	Neurologist	0.071	0.038	0.0	2.44e-04
	AI-augmented Neurologist	0.345	0.078		
ODE	Neurologist	0.113	0.091	9.0	8.06e-03
	AI-augmented Neurologist	0.120	0.106		
AD	Radiologist	0.728	0.112	0.0	7.81e-03
	AI-augmented Radiologist	0.743	0.072		
LBD	Radiologist	0.129	0.037	0.0	7.81e-03
	AI-augmented Radiologist	0.205	0.178		
VD	Radiologist	0.353	0.088	5.0	7.81e-02
	AI-augmented Radiologist	0.360	0.104		
PRD	Radiologist	0.136	0.061	0.0	7.81e-03
	AI-augmented Radiologist	0.460	0.070		
FTD	Radiologist	0.420	0.196	0.0	7.81e-03
	AI-augmented Radiologist	0.606	0.184		
NPH	Radiologist	0.433	0.239	3.0	3.91e-02
	AI-augmented Radiologist	0.423	0.283		
SEF	Radiologist	0.111	0.073	0.0	7.81e-03
	AI-augmented Radiologist	0.145	0.089		
PSY	Radiologist	0.259	0.063	0.0	7.81e-03
	AI-augmented Radiologist	0.392	0.085		
TBI	Radiologist	0.104	0.016	3.0	3.91e-02
	AI-augmented Radiologist	0.115	0.012		
ODE	Radiologist	0.137	0.044	0.0	7.81e-03
	AI-augmented Radiologist	0.153	0.080		

Table S15: **Statistical analysis of AI-augmented clinician APs.** Detailed statistics for individual clinicians' AP and AI-augmented clinicians' AP. Median consensus confidence score and interquartile range (IQR) are presented. One-tailed Wilcoxon signed-rank test was performed to test for increase in performance for each label without any corrections made for multiple comparisons, and the test W statistic and its associated p-value are presented.

Dataset (group)	Balanced Accuracy	Precision	Sensitivity	Specificity	F1 Score	MCC	AUROC	AUPR
NACC								
NC	0.93	0.88	0.92	0.93	0.90	0.85	0.98	0.96
MCI	0.75	0.66	0.55	0.94	0.60	0.53	0.90	0.62
DE	0.93	0.92	0.93	0.94	0.93	0.87	0.98	0.98
AD	0.89	0.83	0.89	0.90	0.86	0.78	0.96	0.93
LBD	0.79	0.74	0.58	0.99	0.65	0.64	0.96	0.70
VD	0.66	0.64	0.34	0.99	0.44	0.45	0.94	0.51
PRD	0.53	1.00	0.059	1.00	0.11	0.24	0.96	0.14
FTD	0.72	0.80	0.45	0.99	0.58	0.58	0.95	0.68
NPH	0.50	NaN	0	1.00	NaN	NaN	0.90	0.11
SEF	0.50	NaN	0	1.00	NaN	NaN	0.83	0.07
PSY	0.54	0.64	0.073	1.00	0.13	0.20	0.90	0.36
TBI	0.50	0	0	1.00	NaN	0	0.89	0.09
ODE	0.50	NaN	0	1.00	NaN	NaN	0.84	0.11
ADNI								
NC	0.83	0.65	0.97	0.70	0.78	0.65	0.93	0.84
MCI	0.67	0.87	0.38	0.95	0.53	0.41	0.84	0.80
DE	0.90	0.64	0.91	0.89	0.75	0.70	0.96	0.86
AD	0.90	0.69	0.88	0.92	0.77	0.72	0.96	0.84
FHS								
NC	0.66	0.34	0.82	0.49	0.48	0.27	0.66	0.32
MCI	0.51	0.60	0.028	0.99	0.053	0.085	0.53	0.31
DE	0.62	0.73	0.39	0.86	0.51	0.28	0.70	0.68
AD	0.62	0.68	0.36	0.88	0.47	0.28	0.70	0.62
LBD	0.50	0	0	0.99	NaN	-0.015	0.58	0.059
VD	0.53	0.70	0.062	1.00	0.11	0.20	0.72	0.28
FTD	0.56	0.14	0.12	1.00	0.13	0.13	0.77	0.061

Table S16: **Model performance without using focal loss.** This table presents the performance metrics of our model trained using binary cross-entropy loss across the NACC, ADNI, and FHS datasets. Comparing the results with Table S7, the model trained with only binary cross-entropy loss shows significantly lower balanced accuracy values for etiologies with high data imbalance indicating the importance of using focal loss to improve the model's ability to accurately classify instances of underrepresented etiologies. The focal loss function, by design, applies a modulating term to the cross-entropy loss in order to focus learning on hard-to-classify examples, which are often found in minority classes. This approach also contributes to the overall robustness of the model by ensuring that it does not become biased towards the majority class.

Label	Rater	Ground Truth Group	Median	IQR	U	p-value
NC	Neurologist	True Positive	92.00	34.29	-44.83	2.77e-33
		True Negative	6.17	9.25		
	Model	True Positive	86.70	22.95	-51.07	1.84e-45
		True Negative	10.60	11.90		
MCI	Neurologist	True Positive	57.25	19.04	-7.02	2.34e-08
		True Negative	22.92	32.92		
	Model	True Positive	56.40	19.40	-5.93	3.35e-06
		True Negative	28.70	31.50		
DE	Neurologist	True Positive	80.92	31.81	-22.43	7.58e-25
		True Negative	7.00	16.15		
	Model	True Positive	76.90	23.28	-23.40	1.28e-24
		True Negative	12.25	28.73		
AD	Neurologist	True Positive	60.25	22.50	-10.00	1.25e-16
		True Negative	26.33	26.96		
	Model	True Positive	65.90	18.90	-15.58	1.30e-25
		True Negative	34.50	31.75		
LBD	Neurologist	True Positive	60.42	19.92	-9.32	5.20e-06
		True Negative	6.00	22.04		
	Model	True Positive	75.20	21.70	-51.02	1.24e-29
		True Negative	20.70	17.00		
VD	Neurologist	True Positive	29.42	15.12	-3.88	1.70e-03
		True Negative	13.67	12.58		
	Model	True Positive	56.70	29.70	-8.52	7.52e-07
		True Negative	28.50	20.30		
PRD	Neurologist	True Positive	1.58	0.79	-2.27	5.30e-02
		True Negative	0.92	0.75		
	Model	True Positive	24.70	16.75	-12.02	1.62e-10
		True Negative	3.40	7.30		
FTD	Neurologist	True Positive	34.08	17.17	-4.96	4.48e-04
		True Negative	7.08	11.00		
	Model	True Positive	74.40	10.65	-9.82	1.07e-07
		True Negative	33.20	31.50		
NPH	Neurologist	True Positive	20.58	22.62	-12.87	1.40e-07
		True Negative	1.08	1.42		
	Model	True Positive	32.90	15.60	-2.58	3.77e-02
		True Negative	14.40	14.00		
SEF	Neurologist	True Positive	2.42	2.75	-1.03	3.31e-01
		True Negative	1.50	2.79		
	Model	True Positive	42.50	8.38	-2.34	4.27e-02
		True Negative	37.35	16.12		
PSY	Neurologist	True Positive	18.42	19.96	-3.59	1.98e-03
		True Negative	7.17	10.75		
	Model	True Positive	54.10	24.00	-5.81	6.08e-06
		True Negative	38.80	25.90		
TBI	Neurologist	True Positive	1.17	0.50	-0.87	4.13e-01
		True Negative	1.00	0.67		
	Model	True Positive	33.40	5.15	-9.78	1.12e-10
		True Negative	20.10	11.20		
ODE	Neurologist	True Positive	5.17	7.08	-0.80	4.48e-01
		True Negative	1.75	4.04		
	Model	True Positive	42.90	12.90	-0.60	5.64e-01
		True Negative	47.10	19.30		

Table S17: **Statistical analysis of confidence scores provided by neurologists and by the model.** Neurologists (n=12) were given 100 randomly selected cases encompassing individual-level demographics, health history, neurological tests, physical as well as neurological examinations, and multi-sequence MRI scans. The neurologists were then tasked with assigning confidence scores for NC, MCI, DE, and the 10 dementia etiologies: AD, LBD, VD, PRD, FTD, NPH, SEF, PSY, TBI, and ODE (see Glossary 1). Neurologists' confidence scores were averaged to produce a single consensus confidence score for each case, for which median values and interquartile ranges (IQR) are presented for each ground truth and label group. P-values and U-statistics for differences in confidence scores for true negative and true positive cases were determined with the two-tailed Brunner-Munzel test, with no corrections made for multiple comparisons.

Label	Rater	Ground Truth Group	Median	IQR	U	p-value
AD	Radiologist	True Positive	12.90	30.90	-0.37	7.15e-01
		True Negative	12.00	16.00		
	Model	True Positive	12.90	30.90	-0.37	7.15e-01
		True Negative	12.00	16.00		
LBD	Radiologist	True Positive	38.90	44.10	-0.09	9.29e-01
		True Negative	36.20	30.65		
	Model	True Positive	38.90	44.10	-0.09	9.29e-01
		True Negative	36.20	30.65		
VD	Radiologist	True Positive	49.60	54.55	2.78	1.29e-02
		True Negative	70.60	37.00		
	Model	True Positive	49.60	54.55	2.78	1.29e-02
		True Negative	70.60	37.00		
PRD	Radiologist	True Positive	55.00	18.85	0.01	9.94e-01
		True Negative	41.50	36.30		
	Model	True Positive	55.00	18.85	0.01	9.94e-01
		True Negative	41.50	36.30		
FTD	Radiologist	True Positive	22.70	17.75	0.38	7.11e-01
		True Negative	22.80	20.70		
	Model	True Positive	22.70	17.75	0.38	7.11e-01
		True Negative	22.80	20.70		
NPH	Radiologist	True Positive	37.20	6.45	-2.08	6.24e-02
		True Negative	30.10	24.20		
	Model	True Positive	37.20	6.45	-2.08	6.24e-02
		True Negative	30.10	24.20		
SEF	Radiologist	True Positive	3.35	4.83	0.39	7.03e-01
		True Negative	4.60	10.10		
	Model	True Positive	3.35	4.83	0.39	7.03e-01
		True Negative	4.60	10.10		
PSY	Radiologist	True Positive	30.50	23.85	1.30	2.08e-01
		True Negative	36.00	38.20		
	Model	True Positive	30.50	23.85	1.30	2.08e-01
		True Negative	36.00	38.20		
TBI	Radiologist	True Positive	12.50	9.65	1.25	2.48e-01
		True Negative	14.90	15.00		
	Model	True Positive	12.50	9.65	1.25	2.48e-01
		True Negative	14.90	15.00		
ODE	Radiologist	True Positive	40.80	10.70	0.13	8.99e-01
		True Negative	38.40	15.80		
	Model	True Positive	40.80	10.70	0.13	8.99e-01
		True Negative	38.40	15.80		

Table S18: **Statistical analysis of confidence scores provided by radiologists and by the model.** Radiologists (n=7) were given 70 randomly selected cases with a confirmed dementia diagnosis encompassing individual-level demographics and multi-sequence MRI scans. The radiologists were tasked with assigning confidence scores for the 10 dementia etiologies. Radiologists' confidence scores were averaged to produce a single consensus confidence score for each case, for which median values and interquartile ranges (IQR) are presented for each ground truth and label group. P-values and U-statistics for differences in confidence scores for true negative and true positive cases were determined with the two-tailed Brunner-Munzel test, with no corrections made for multiple comparisons.

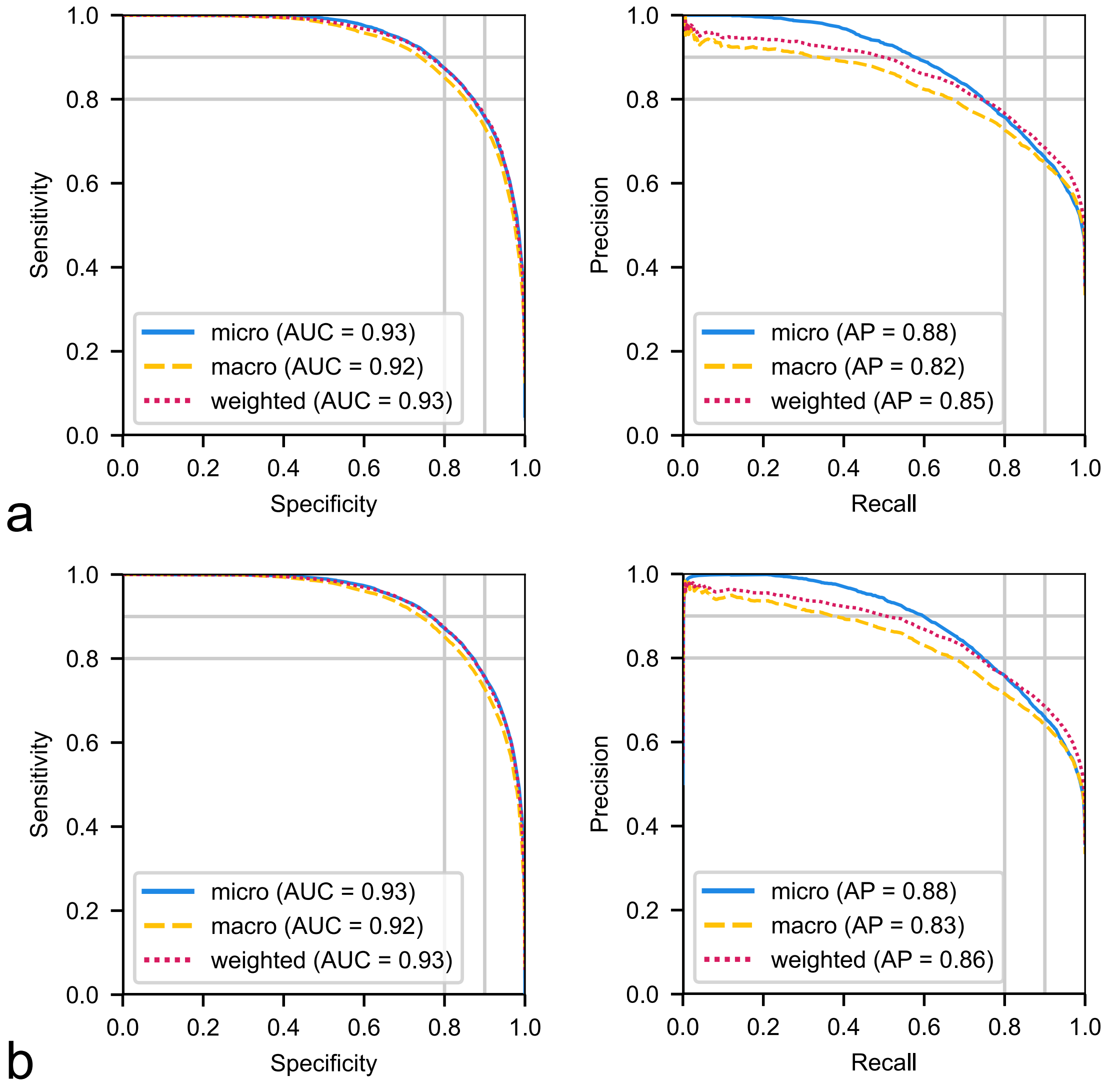

Figure S1: **ROC and PR curves across age groups averaged over the cognitive spectrum in NACC testing, ADNI and FHS.** ROC and PR curves, with their respective micro-average, macro-average, and weighted-average calculations based on the labels for NC, MCI, and DE on (a) individuals under the age of 74 (6188 cases), and (b) individuals over the age of 74 (6188 cases). 74 was the median age of our testing population.

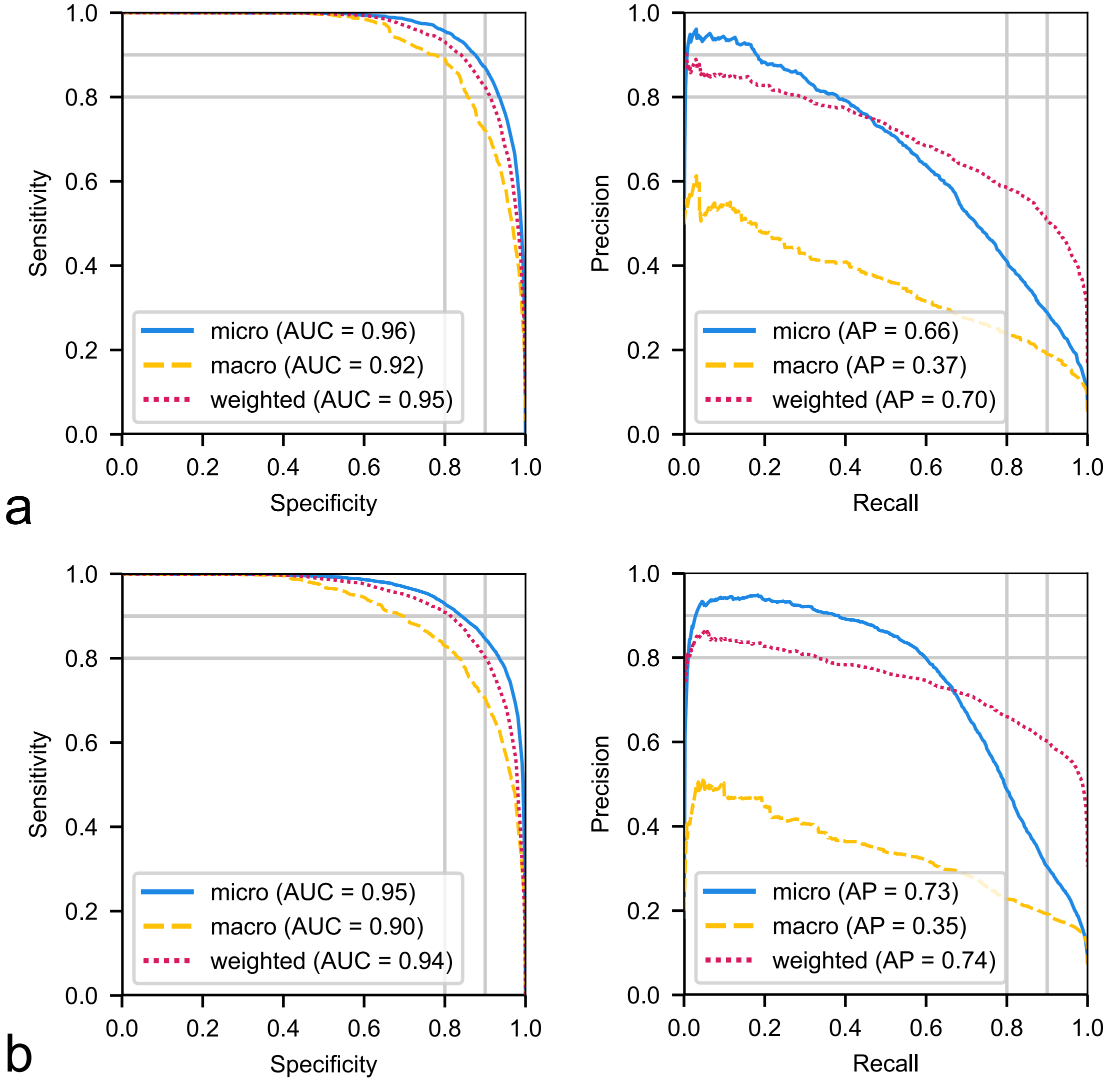

Figure S2: **ROC and PR curves across different age groups averaged over dementia diagnostic labels in NACC testing.** ROC and PR curves, with their respective micro-, macro-, and weighted-average values across the 10 dementia diagnostic labels on (a) individuals under the age of 74 (4550 cases), and (b) individuals over the age of 74 (4345 cases). 74 is the median age of the testing population.

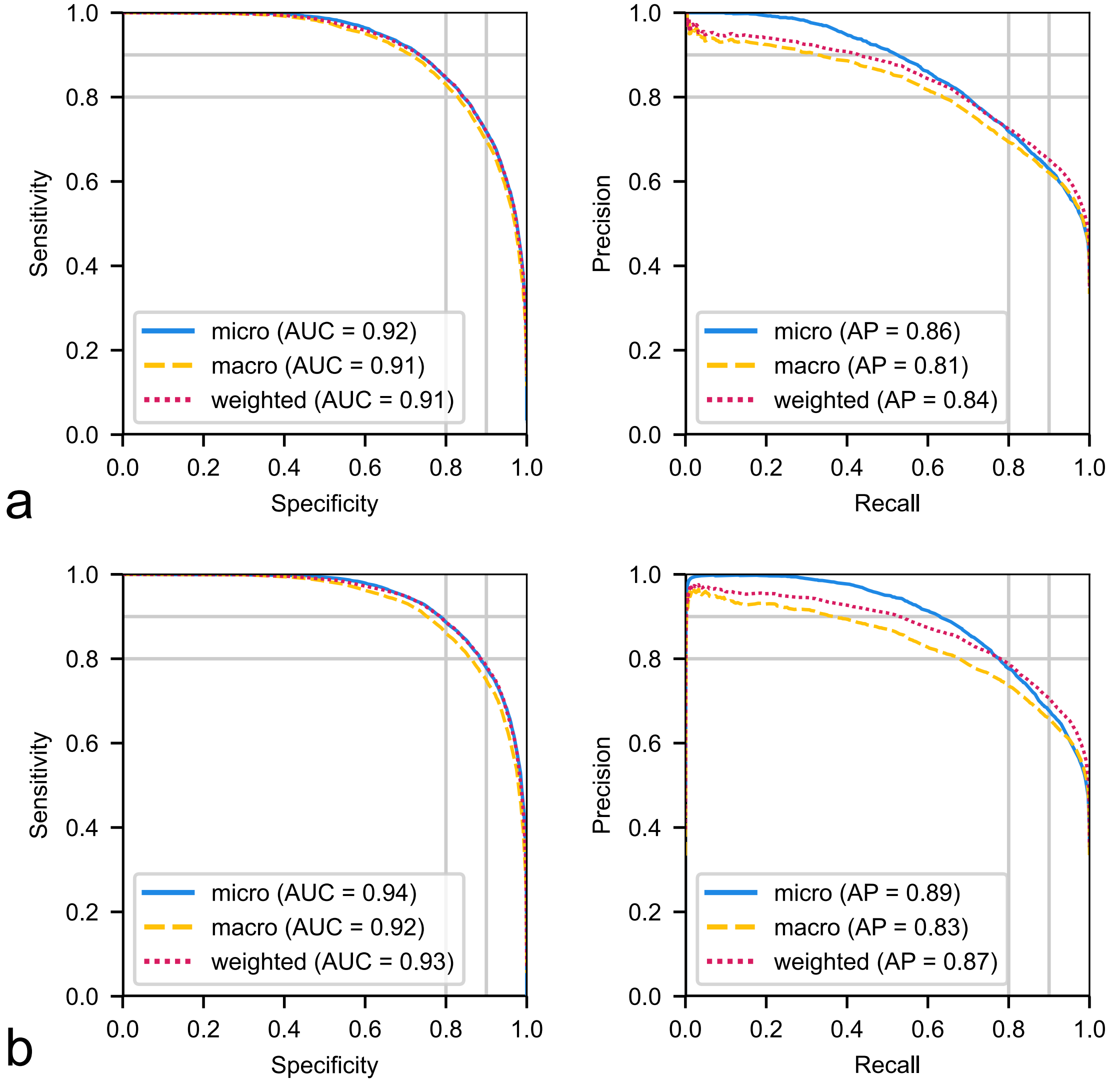

Figure S3: **ROC and PR curves across gender groups averaged over the cognitive spectrum in NACC testing, ADNI and FHS.** ROC and PR curves, with their respective micro-, macro-, and weighted-averages based on the labels for NC, MCI, and DE on (a) individuals identified as male (5809 cases), and (b) individuals identified as female (7141 cases).

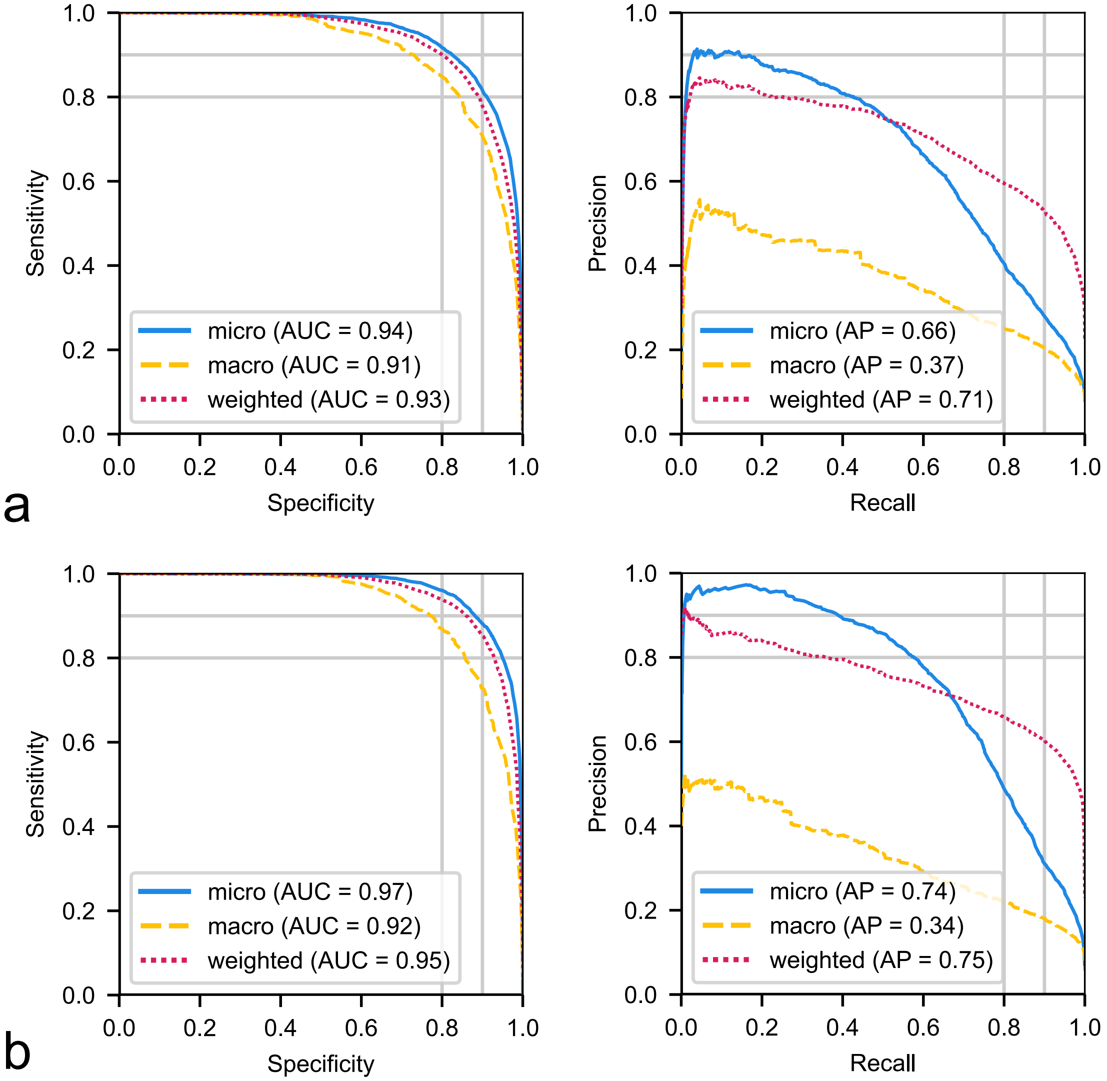

Figure S4: **ROC and PR curves across gender groups averaged over dementia diagnostic labels in NACC testing.** ROC and PR curves, with their respective micro-average, macro-average, and weighted-average calculations across the 10 dementia etiologies on (a) individuals identified as male (3850 cases), and (b) individuals identified as female (5045 cases).

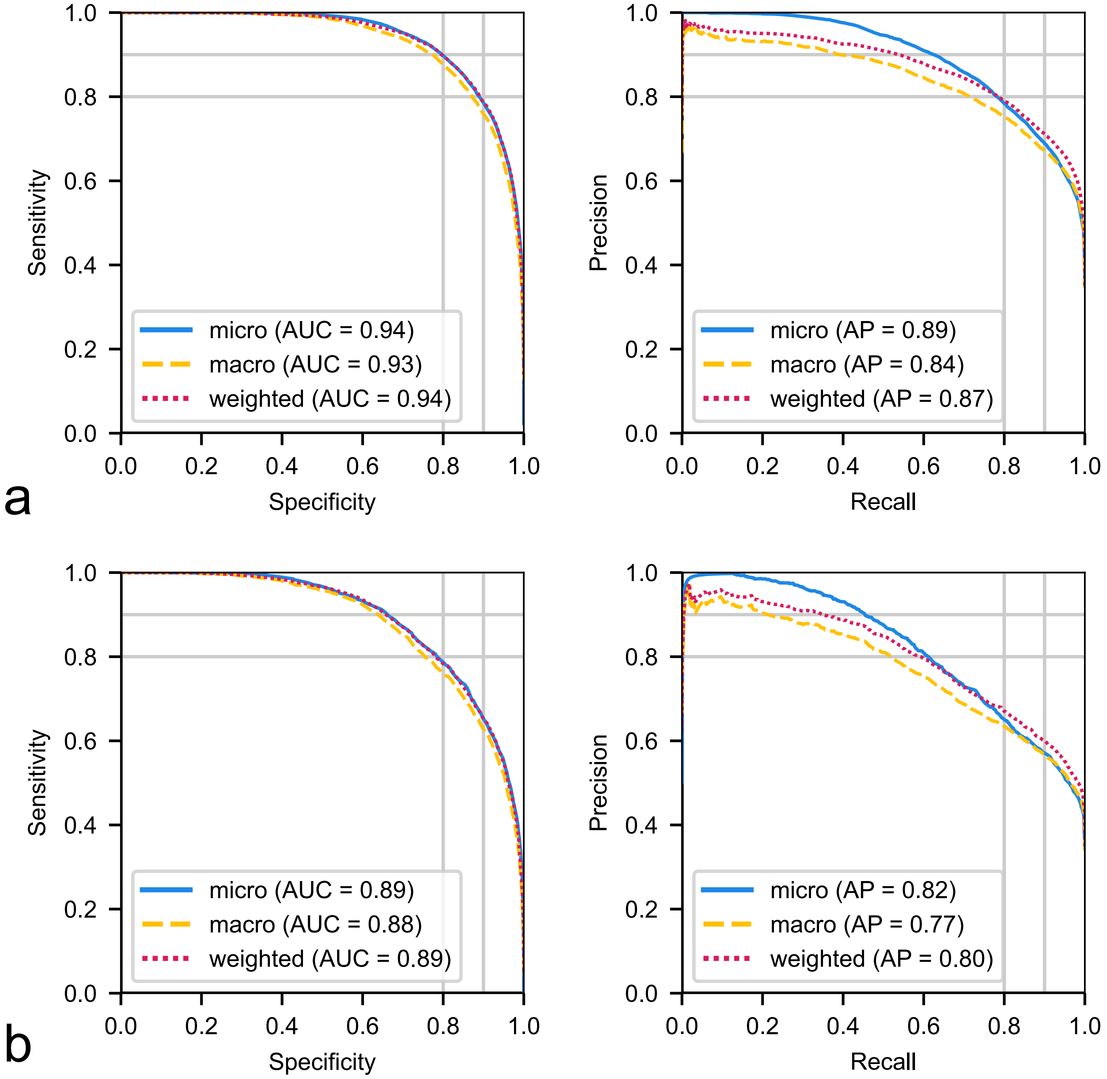

Figure S5: **ROC and PR curves across race groups averaged over the cognitive spectrum in NACC testing, ADNI and FHS.** ROC and PR curves, with their respective micro-, macro-, and weighted-average calculations based on the labels for NC, MCI, and DE on (a) individuals identified as White (10965 cases), and (b) individuals identified as Black or African American, Asian, Native Hawaiian or Pacific Islander, American Indian or Alaska Native, or individuals with multiple races (1985 cases). Only two race categories were chosen due to the relatively low sample size of non-White individuals in these cohorts.

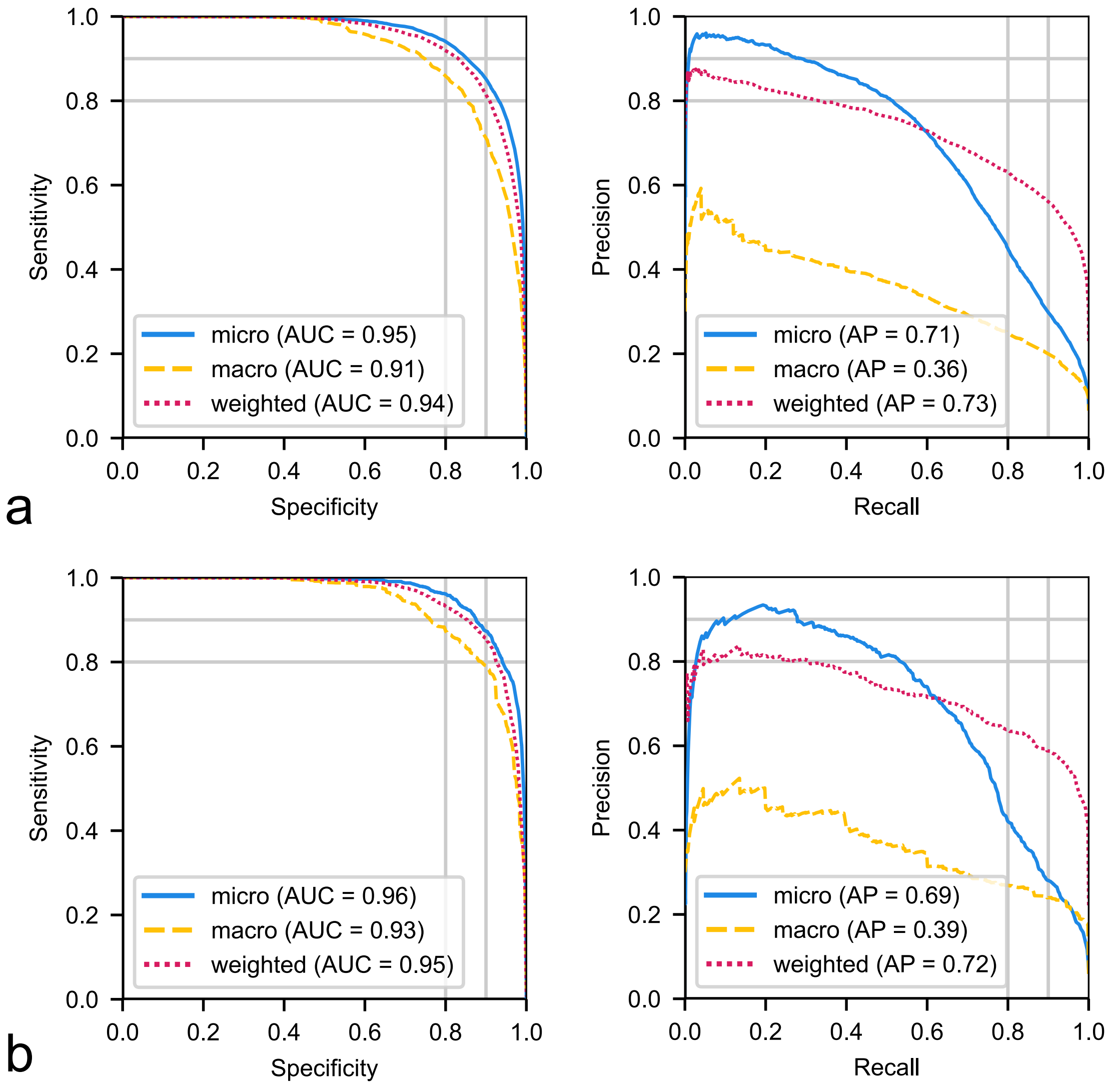

Figure S6: **ROC and PR curves across race groups averaged over dementia diagnostic labels in NACC testing.** ROC and PR curves, with their respective micro-, macro-, and weighted-average values across the 10 dementia etiologies on (a) individuals identified as White (7178 cases), and (b) individuals identified as Black or African American, Asian, Native Hawaiian or Pacific Islander, American Indian or Alaska Native, or individuals with multiple races (1717 cases). Only two race categories were chosen due to the relatively low sample size of non-White individuals in these cohorts.
