# Supplementary material for: AI-based differential diagnosis of dementia etiologies on multimodal data
Source: Nat Med. 2024 Jul 4;30(10):2977–89. doi: 10.1038/s41591-024-03118-z (PMC11485262; doi:10.1038/s41591-024-03118-z)
Supplement: Supplementary file 2 — Reporting Summary [file 41591_2024_3118_MOESM2_ESM.pdf]

nature portfolio

Corresponding author(s): Vijaya B. Kolachalama, PhD

Last updated by author(s): Jun 21, 2024

Reporting Summary

Nature Portfolio wishes to improve the reproducibility of the work that we publish. This form provides structure for consistency and transparency in reporting. For further information on Nature Portfolio policies, see our Editorial Policies and the Editorial Policy Checklist.

Statistics

For all statistical analyses, confirm that the following items are present in the figure legend, table legend, main text, or Methods section.

n/a	Confirmed	
☐	☒	The exact sample size (*n*) for each experimental group/condition, given as a discrete number and unit of measurement
☐	☒	A statement on whether measurements were taken from distinct samples or whether the same sample was measured repeatedly
☐	☒	The statistical test(s) used AND whether they are one- or two-sided<br>*Only common tests should be described solely by name; describe more complex techniques in the Methods section.*
☐	☒	A description of all covariates tested
☐	☒	A description of any assumptions or corrections, such as tests of normality and adjustment for multiple comparisons
☐	☒	A full description of the statistical parameters including central tendency (e.g. means) or other basic estimates (e.g. regression coefficient) AND variation (e.g. standard deviation) or associated estimates of uncertainty (e.g. confidence intervals)
☐	☒	For null hypothesis testing, the test statistic (e.g. *F*, *t*, *r*) with confidence intervals, effect sizes, degrees of freedom and *P* value noted<br>*Give P values as exact values whenever suitable.*
☒	☐	For Bayesian analysis, information on the choice of priors and Markov chain Monte Carlo settings
☒	☐	For hierarchical and complex designs, identification of the appropriate level for tests and full reporting of outcomes
☐	☒	Estimates of effect sizes (e.g. Cohen's *d*, Pearson's *r*), indicating how they were calculated

Our web collection on statistics for biologists contains articles on many of the points above.

Software and code

Policy information about availability of computer code

Data collection	We used REDCap (version 11.1.3) to generate the questionnaire for clinicians. All clinicians reviewed MRIs using 3D Slicer (version 4.10.2) and logged their findings in REDCap (version 11.1.3).
Data analysis	Our software development utilized Python (version 3.11.7) and the models were developed using PyTorch (version 2.1.0). We used several other Python libraries to support data analysis, including pandas (version 1.5.3), scipy (version 1.10.1), tensorboardX (version 2.6.2), torchvision (version 0.15) and scikit-learn (version 1.2.2). Figures were prepared using Canva and Adobe Illustrator. Python scripts as well as help files along with information on the study population are made available on GitHub https://github.com/vkola-lab/nmed2024.

For manuscripts utilizing custom algorithms or software that are central to the research but not yet described in published literature, software must be made available to editors and reviewers. We strongly encourage code deposition in a community repository (e.g. GitHub). See the Nature Portfolio guidelines for submitting code & software for further information.

Data

Policy information about availability of data

All manuscripts must include a data availability statement. This statement should provide the following information, where applicable:

- Accession codes, unique identifiers, or web links for publicly available datasets
- A description of any restrictions on data availability
- For clinical datasets or third party data, please ensure that the statement adheres to our policy

This study includes data from the following nine cohorts: National Alzheimer's Coordinating Center (NACC), Alzheimer's Disease Neuroimaging Initiative (ADNI), Frontotemporal Lobar Degeneration Neuroimaging Initiative (NIFD), Parkinson's Progression Marker Initiative (PPMI), Australian Imaging, Biomarker and Lifestyle Flagship Study of Ageing (AIBL), Open Access Series of Imaging Studies (OASIS), 4 Repeat Tauopathy Neuroimaging Initiative (4RTNI), Lewy Body Dementia Center for Excellence at Stanford University (LBDSU), and Framingham Heart Study (FHS). Data from ADNI, AIBL, NIFD, PPMI and 4RTNI can be downloaded from the LONI website at https://ida.loni.usc.edu. NACC and OASIS data can be downloaded at https://naccdata.org and https://sites.wustl.edu/oasisbrains/, respectively. Finally, data from FHS https://www.framinghamheartstudy.org and LBDSU https://med.stanford.edu/poston-lab/LBD.html can be obtained upon request, subject to institutional approval. We used the Montreal Neuroimaging Institute MNI152 template for image processing purposes, and the template can be downloaded at http://www.bic.mni.mcgill.ca/ServicesAtlases/ICBM152NLin2009.

Human research participants

Policy information about studies involving human research participants and Sex and Gender in Research.

Reporting on sex and gender	The data was obtained from existing cohorts. We reported sex-related information on all the cohorts whenever available. We also performed sex-specific subgroup analysis.
Population characteristics	Participants in the study were 51,269 individuals with a mean age of 73. They included 22,349 males and 28,920 females. The ethnic composition was 40,335 White, 5,840 Black or African American, 1,285 Asian, 276 American Indian or Alaskan Native, 38 Native Hawaiian or Pacific Islander and 1,430 Multiracial. All participants were screened for cognitive impairment, with 19,849 classified as having normal cognition, 9,357 as having mild cognitive impairment (MCI) and 22,063 as having dementia. More details on the diagnostic information can be found in the Methods section.
Recruitment	We did not recruit any participants for this study.
Ethics oversight	The data collection for the Framingham Heart Study and the Lewy Body Dementia Center for Excellence at Stanford University was approved by the respective institutional review boards.

Note that full information on the approval of the study protocol must also be provided in the manuscript.

Field-specific reporting

Please select the one below that is the best fit for your research. If you are not sure, read the appropriate sections before making your selection.

☒ Life sciences ☐ Behavioural & social sciences ☐ Ecological, evolutionary & environmental sciences

For a reference copy of the document with all sections, see nature.com/documents/nr-reporting-summary-flat.pdf

Life sciences study design

All studies must disclose on these points even when the disclosure is negative.

Sample size	We obtained data from all the nine cohorts. No sample size calculation was carried out. We considered all subjects satisfying the inclusion and exclusion criterion described in the manuscript. We included 38,319 participants for training and 12,950 participants for testing the model. More details on the study population can be found in the manuscript.
Data exclusions	We excluded data from the cohorts if the diagnosis information (normal cognition (NC), mild cognitive impairment (MCI), or dementia (DE)) was not available.
Replication	The results can be replicated by following the methods described in the manuscript or by running the code available in our GitHub repository. The data from ADNI, NACC, AIBL, NIFD, PPMI, OASIS, and 4RTNI are open access. Additional data requests are required to access the data from FHS and LBDSU for replicating the findings from our study.
Randomization	When building the deep learning model, the cases were shuffled using a consistent random seed and were split into train, validation and testing sets using stratified sampling at person level.
Blinding	In the comparison of clinicians versus deep learning model performance, clinicians were blinded to the documented clinical diagnoses of the cases presented.

Reporting for specific materials, systems and methods

We require information from authors about some types of materials, experimental systems and methods used in many studies. Here, indicate whether each material, system or method listed is relevant to your study. If you are not sure if a list item applies to your research, read the appropriate section before selecting a response.

Materials & experimental systems

n/a	Involved in the study
☒	☐ Antibodies
☒	☐ Eukaryotic cell lines
☒	☐ Palaeontology and archaeology
☒	☐ Animals and other organisms
☒	☐ Clinical data
☒	☐ Dual use research of concern

Methods

n/a	Involved in the study
☒	☐ ChIP-seq
☒	☐ Flow cytometry
☐	☒ MRI-based neuroimaging

Magnetic resonance imaging

Experimental design

Design type: Resting state structural MRI

Design specifications: We used multiple scan sequences including T1-weighted, T2-weighted, FLAIR and SWI scans whenever available. Detailed descriptions of the scan protocols and design specifications can be obtained from the respective websites of the study cohorts.

Behavioral performance measures: Not applicable

Acquisition

Imaging type(s): Structural

Field strength: 1.5 or 3 Tesla

Sequence & imaging parameters: T1-weighted, T2-weighted, FLAIR, SWI sequences

Area of acquisition: Whole brain

Diffusion MRI: ☐ Used ☒ Not used

Preprocessing

Preprocessing software: The collected imaging data were stored in the NIFTI file format, categorized by participant and the date of their visit. The MRI scans underwent a singular pre-processing step, which involved skull stripping using SynthStrip, a computational tool designed for extracting brain voxels from various image types. MRI scans were linearly registered based on the MNI152 atlas To ensure the purity of the dataset, we excluded calibration, localizer, and 2D scans from the downloaded data before initiating model training.

Normalization: See previous response

Normalization template: MNI152

Noise and artifact removal: All MRI scans were normalized to the range [0,1] to increase the homogeneity of the data.

Volume censoring: No volume censoring was used in this study.

Statistical modeling & inference

Model type and settings: Our model employs the transformer architecture to process diverse diagnostic data, including demographics, medical history, neuroimaging, functional assessments, and neuropsychological test scores. Each data type is first transformed into a fixed-length vector using a tailored approach, creating the initial input layer for the transformer. The transformer then synthesizes these vector inputs, interpreting and converting them into a coherent series of diagnostic predictions, effectively leveraging the complex interplay of varied health-related parameters.

Effect(s) tested: Task- and stimulus-related effects were not tested in this study.

Specify type of analysis: ☒ Whole brain ☐ ROI-based ☐ Both

Statistic type for inference (See Eklund et al. 2016)	We used Shapley analysis to perform feature importance analysis.
Correction	We applied the Kruskal-Wallis H-test for independent samples and subsequently conducted post-hoc Dunn’s testing with Bonferroni correction to evaluate the relationship between clinical dementia rating scores and the model-predicted probabilities.

Models & analysis

n/a	Involved in the study
☒	☐ Functional and/or effective connectivity
☒	☐ Graph analysis
☐	☒ Multivariate modeling or predictive analysis

Multivariate modeling and predictive analysis	We summarized our model results using area under receiver operating characteristic curves (AUROC) and precision-recall curves (AUPR). Also, model accuracy, sensitivity, specificity, F1-score and Matthew's correlation coefficient values were reported.
